# The oxidation state in low-valent beryllium and magnesium compounds†

**DOI:** 10.1039/d2sc01401g

**Published:** 2022-05-09

**Authors:** Martí Gimferrer, Sergi Danés, Eva Vos, Cem B. Yildiz, Inés Corral, Anukul Jana, Pedro Salvador, Diego M. Andrada

**Affiliations:** Institut de Química Computacional i Catàlisi, Departament de Química, Universitat de Girona c/M. Aurelia Capmany 69 17003 Girona Spain pedro.salvador@udg.edu; General and Inorganic Chemistry Department, University of Saarland, Campus C4.1 66123 Saarbruecken Germany diego.andrada@uni-saarland.de; Departamento de Química, Universidad Autónoma de Madrid c/Francisco Tomás y Valiente 7 28049 Cantoblanco Madrid Spain ines.corral@uam.es; Department of Medicinal and Aromatic Plants, Aksaray University Hacilar harmani 2 68100 Aksaray Turkey; Tata Institute of Fundamental Research Hyderabad, Gopanpally Hyderabad-500046 Telangana India ajana@tifrh.res.in

## Abstract

Low-valent group 2 (E = Be and Mg) stabilized compounds have been long synthetically pursued. Here we discuss the electronic structure of a series of Lewis base-stabilized Be and Mg compounds. Despite the accepted zero(0) oxidation state nature of the group 2 elements of some recent experimentally accomplished species, the analysis of multireference wavefunctions provides compelling evidence for a strong diradical character with an oxidation state of +2. Thus, we elaborate on the distinction between a description as a donor–acceptor interaction L(0) ⇆ E(0) ⇄ L(0) and the internally oxidized situation, better interpreted as a diradical L(−1) → E(+2) ← L(−1) species. The experimentally accomplished examples rely on the strengthened bonds by increasing the π-acidity of the ligand; avoiding this interaction could lead to an unprecedented low-oxidation state.

## Abstract 2

This article examines the electronic structure of group 2 (E = Be and Mg) ligand stabilized compounds. We elaborate on the donor–acceptor interaction L(0) ⇆ E(0) ⇆ L(0) and diradical L(−1) → E(+2) ← L(−1) pictures to assess the oxidation state of the metal.

## Graphical abstract

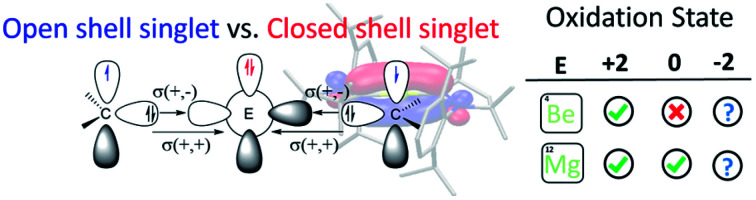

## Introduction

The scope of the concept of oxidation states in main group compounds has remarkably expanded in the last two decades.^1–3^ Stable singlet carbenes featuring non-oxidative electron-pair donation such as *N*-heterocyclic carbenes (NHCs)^4^ and cyclic(alkyl)(amino) carbenes (cAACs)^5,6^ have been crucial to preparatively access unique low oxidation states. For the description of chemical bonding, donor–acceptor interactions have been invoked, traditionally connected to transition metals.^7^ Thus, the formal electron deficiency of the central atom is alleviated by σ-donation from the ligand, which is counterbalanced by a somewhat weaker π-backdonation.^7,8^ While the chemistry of low-valent p-block compounds has substantially benefitted from this coordinative bonding concept, the s-block chemistry has lagged behind.^1^

**Scheme 1:**
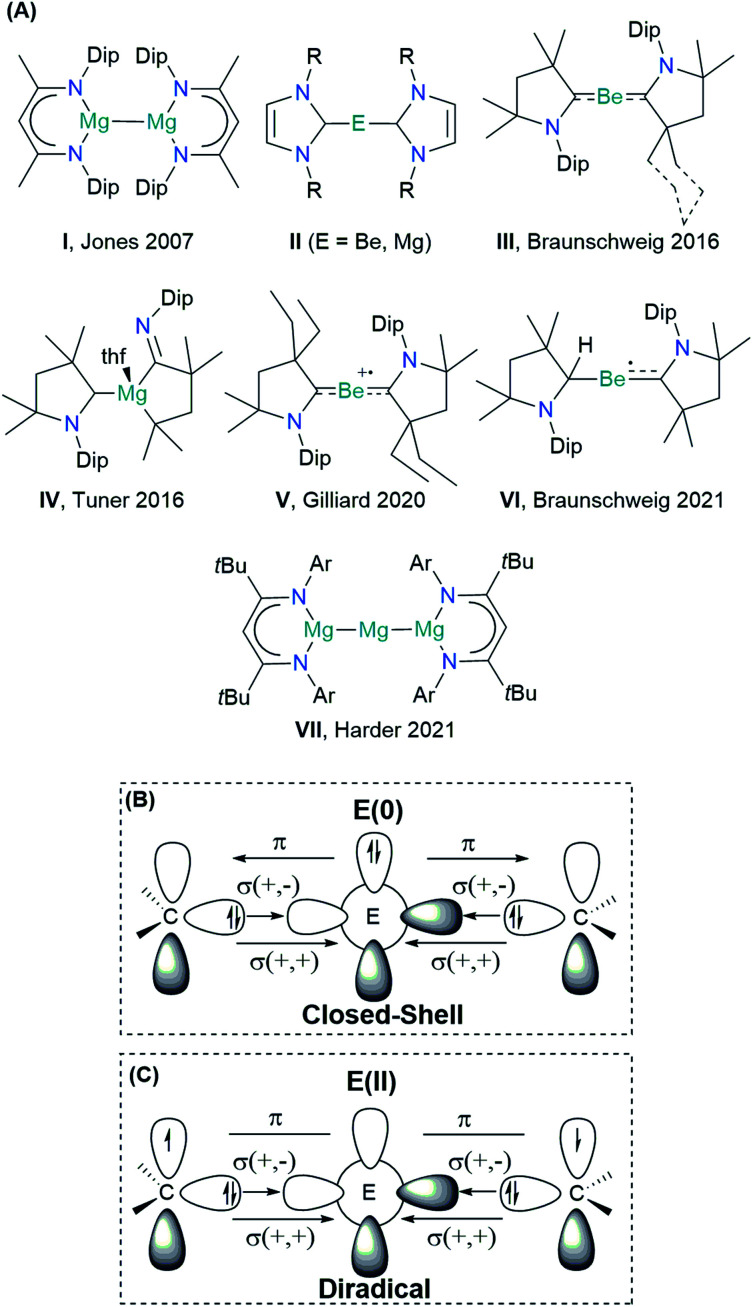
(A) Schematic view of the s-block low-valent main group compounds experimentally achieved: Dip = 2,6-di*iso*propyl-phenyl; R = H, methyl, phenyl; Ar = 2,6-(3-pentyl)-phenyl. Schematic view of the orbital interactions in E(0)L_2_ (L = NHC and cAAC): (B) donor–acceptor interaction in the singlet closed-shell electronic state, and (C) electron-sharing interaction in the open-shell singlet electronic state (diradical). Notation “+,+” and “+,−” stand for the in-phase and out-of-phase combination of lone-pair orbitals.

The Group 2 chemistry has been long dominated by the +2 oxidation state, given the strong propensity of these elements to lose the valence electrons. Significant progress in the obtention of species with different oxidation states has been reported by Jones and co-workers on the Mg(+1) dimer compound I (Scheme 1), containing Mg^I^–Mg^I^ bonds stabilized by guanidinate or β-diketiminate (NacNac) ligands.^9^ Over the years, the Jones compound has evolved from curiosity into a highly selective reducing agent.^9,10^ Although species with a Be^I^–Be^I^ single bond have been computationally predicted, stable molecules featuring this bonding motif remain unrealized so far.^11–14^ Zero-valent Be(0) or Mg(0) compounds II were also elusive until recently. In 2016, Braunschweig and co-workers reported the seminal isolation of dicoordinated neutral Be(0)(cAAC)_2_ complexes III.^15^ The unusual bonding situation has been rationalized in terms of donor–acceptor interactions between cAAC ligands acting as σ-donors to empty s-type orbitals of a Be(0) atom, which would have available p-type electrons to furnish a significantly strong π-backdonation towards the ligands, cAAC ← Be → cAAC (Scheme 1B).

This bonding scheme provides access to a stable beryllium radical cation V and a neutral species VI, with beryllium in the formal oxidation state of +1.^16,17^ Attempts to prepare the Mg(0) congener were unsuccessful, leading instead to the ligand activation product IV.^18^ The reaction outcome has been ascribed to the formation of the highly reactive Mg(0)(cAAC)_2_ species, followed by ligand rearrangement. Only recently, large charge transfer has been recognized from Mg to the cAAC ligands.^19^

The quest for Mg(0) compound has been recently fulfilled by Harder and co-workers, using an extraordinarily bulky ligand (BDI* = HC{C(*t*Bu)N[2,6-(3-pentyl)-phenyl]}_2_) to stabilize the Mg(+2) precursor.^20^ The reduction with sodium powder furnished a Mg(0) compound {[(BDI*)Mg^−^][Na^+^]}_2_, which upon heating yielded a three-magnesium atom cluster VII (recall Scheme 1). Notably, the bonding situation of the latter differs from that of the previous species, as the stability is driven by two Mg–Mg electron-sharing bonds rather than a donor–acceptor interaction.^21^

The oxidation state assessment of these species is connected to the molecular orbital theory picture.^22^ The donor–acceptor interaction in a closed-shell singlet configuration, similar to that in the Dewar–Chatt–Duncanson (DCD) model, assumes an electronic structure preorganization of E(0) from the ground state ^1^S (ns^2^np^0^) into the doubly excited singlet state ^1^D (ns^0^np^2^) to interact with the σ-donor/π-acceptor ligands (Scheme 1B). Within this description, applying the ionic approximation to the σ- and π-type bonds could indeed lead to the zero oxidation state picture of the alkali earth metal. Note, however, that Be (*Χ*_P_ = 1.57) and Mg (*Χ*_P_ = 1.31) are much less electronegative than C (*Χ*_P_ = 2.55).^23^ Moreover, while the energy for such electron promotion is accessible for transition metals, the experimental gas-phase values for Be and Mg are as high as 178.3 and 399.9 kcal mol^−1^, respectively.^24^ A plausible alternative scenario can be postulated, whereupon bonding of the ligands, the metal centre oxidizes, and its electron pair ends up at the ligands, forming a diradical(oid) species (Scheme 1C). The interaction between the unpaired electrons would be significant in a closed-shell description, leading to the three-centre two-electron system (3c-2e). However, a broken-symmetry solution would suggest a diradical(oid) species, where the paring between the electrons is lower than optimal. A relatively small singlet–triplet gap (Δ*E*_S–T_) is a key indicator for observing the diradical character.^25^

However, distinguishing between these two pictures using single-reference methods is not straightforward, if not impossible, as the incomplete description of the spin polarization can mislead the wavefunction analysis.^26^ Previous investigations have pointed out the multi-reference character of related systems such as germanium Ge(cAAC)_2_ and zinc Zn(cAAC)_2_ counterparts.^27,28^ Herein, we pinpoint the subtle features of prominent low-valent Be- and Mg-based compounds through quantum chemistry calculations.

## Results and discussion

As an outset, we included NHC and cAAC ligands where the flanking groups have different stereoelectronic properties, *i.e.* methyl (Me) and 2,6-di*iso*propyl-phenyl (Dip). Both the singlet and triplet states of the systems were optimized at the B3LYP level of theory. Broken symmetry (BS) solutions, such as the open-shell singlet (OSS), lower in energy were found for the systems stabilized by cAAC ligands. In fact, the closed-shell B3LYP solution for Be-cAAC^Dip^ is not stable. Thus, the ground state is singlet in all cases, either closed-shell or open-shell. The relative energies of the singlet and triplet states at the B3LYP-D3(BJ)/def2-TZVPP level are relatively close, ranging from 7.9 to 2.6 kcal mol^−1^ for Be-NHC^Dip^ and Be-cAAC^Me^, respectively and from 13.3 to 2.4 kcal mol^−1^ for Mg-NHC^Dip^ and Mg-cAAC^Me^, respectively. The triplet state in Mg-cAAC^Dip^ lies 9.1 kcal mol^−1^ below the closed-shell singlet, but the ground state is of OSS nature. Similar observations have been obtained with different functionals (Tables S1–S3†).

**Fig. 1:**
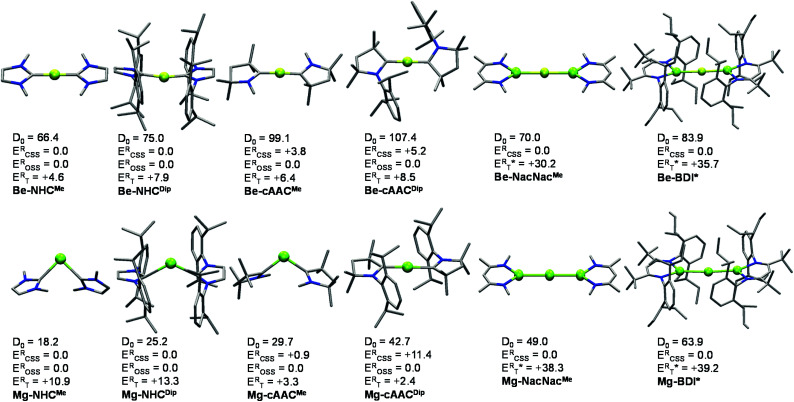
Optimized ground state geometries (B3LYP-D3(BJ)/def2-SVP), dissociation energies (*D*_0_) (B3LYP-D3(BJ)/def2-TZVPP//B3LYP-D3(BJ)/def2-SVP) considering the EL_2_ → E(0) + 2L(0) dissociation, relative electronic energy for the closed shell singlet (E^R^_CSS_), open-shell singlet (E^R^_OSS_) and triplet (E^R^_T_) at the B3LYP/def2-TZVPP level. *Vertical *E*_T_ values. Energies are in kcal mol^−1^. Hydrogen atoms were omitted for clarity.

Fig. 1 displays the ground-state geometries of the studied compounds, together with their dissociation energies (*D*_0_) and singlet/triplet energy differences, and Table 1 shows the numerical values of their main geometrical parameters. The Be–C bond lengths vary slightly with the nature of the ligand, *i.e.* from 1.634 to 1.648 Å, in good agreement with previous studies.^11–15,19^ These values fall in the expected bond lengths of single and double bonds (1.77 and 1.57 Å, respectively).^29^ Moreover, the bond angles are almost collinear for all cases 167.4–179.9°, favouring a strong delocalization on the C–Be–C π-system. On the other hand, the Mg–C bond lengths are shorter than those reported by Couchman *et al.* for Mg_2_(NHC)_2_ and Mg_2_(NHC)_4_ systems.^12^ Note, however, that the computed values are, in fact, longer than expected for a single bond Mg–C (2.14 Å). Only in the case of the Mg-cAAC^Dip^ compound, the coordination distance is within the single and double bond (1.99 Å).^29^ The series of Mg shows an appreciable coordination change, as Mg-cAAC^Dip^ exhibits an almost collinear C–Mg–C angle of 178.9°, while the others possess a rather acute angle (from 90.1° to 119.3°). These structural features have already been described for the Ga^+^(NHC^Dip^)_2_ analogue.^30^ Similarly, the tilted coordination mode of MgL_2_ (L = NHC^Me^, NHC^Dip^ and cAAC^Me^) can be rationalized with a different bonding situation. Here, the two electrons of Mg are not promoted from the s orbital to the p orbital; instead the ligands donate into the p-orbital of Mg, with a backdonation from the occupied s-orbital into the carbene empty orbitals takes place.

**Table 1:** Geometrical parameters (B3LYP), adiabatic singlet-triplet gap (Δ*E*_S–T_), partial atomic charges, *Q*(E), (E = Mg or Be), E-C/Mg bond orders (BO_E–L_),^a^ fragment and inter-fragment local spin (<S^2^>_f_ and <S^2^>_f–f’_), EOS results and global reliability index (R[%]) of the studied compounds in their ground-state at the CASSCF/cc-pVDZ//B3LYP-D3(BJ)/def2-SVP level

E–L_2_ system	Ground state	Δ*E*_S–T_	*d*_E–L_ [Å]	BA_L–E–L_ [°]	*Q*(E)	BO_E-L_	<S^2^>_E_	<S^2^>_L_	<S^2^>_L1–L2_	Be/Mg OS	L OS	*R* (%)
Be–H^c^	CSS	145.7	1.331	180.0	1.39	0.52	0.04	0.02	0.00	+2	−1	100
Be-NHC^Me^	CSS	5.3	1.639	179.9	1.10	0.56	0.06	0.14	−0.11	+2	−1	74.0
Be-NHC^Dip^	CSS	8.0	1.648	167.4	1.12	0.54	0.08	0.22	−0.18	+2	−1	73.8
Be-cAAC^Me^	OSS	8.3	1.634	176.4	1.17	0.56	0.07	0.30	−0.26	+2	−1	77.8
Be-cAAC^Dip^	OSS	8.6	1.644	177.8	1.19	0.54	0.08	0.39	−0.35	+2	−1	78.9
Be-NacNac^Me^^c^	CSS	26.2	2.541	180.0	−0.90	0.61	0.07	0.06	−0.02	−2	+1	86.7
Be-BDI*^b^^c^	CSS	35.7	2.489	177.9	−0.42	0.51	—	—	—	−2	+1	73.0
Mg–H^c^	CSS	123.5	1.699	180.0	1.32	0.58	0.05	0.03	−0.01	+2	−1	100
Mg-NHC^Me^	CSS	9.9	2.300	90.1	0.25	0.27	0.10	0.07	−0.02	0	0	100
Mg-NHC^Dip^	CSS	23.3	2.347	119.3	0.61	0.25	0.09	0.07	−0.02	0	0	82.7
Mg-cAAC^Me^	OSS	5.3	2.174	107.8	0.60	0.35	0.12	0.10	−0.04	0	0	80.0
Mg-cAAC^Dip^	OSS	1.0	2.040	178.9	1.34	0.45	0.08	0.68	−0.64	+2	−1	82.4
Mg-NacNac^Me^^c^	CSS	33.6	2.917	180.0	0.06	0.76	0.12	0.10	−0.02	0	0	59.0
Mg-BDI*^b^^c^	CSS	39.2	2.800	175.4	0.37	0.52	—	—	—	0	0	59.9

a Improved definition of bond orders for correlated wavefunctions from ref. 40.

b Evaluated at the B3LYP-D3(BJ)/def2-SVP level.

c Vertical singlet–triplet gap.

Fig. 1 also summarizes the calculated homolytic dissociation energies (*D*_0_) from the ground state EL_2_ into E(0) (^1^S ground state) and two neutral ligands (EL_2_ → E(0) + 2L(0)). The consistency at different levels of theory is presented in Tables S5–S7 in the ESI.† The *D*_0_ values indicate that the Mg–NHC^Me^ complex (18.2 kcal mol^−1^) is much less stable than the Be–NHC^Me^ analogue (66.4 kcal mol^−1^). The Be-cAAC^Dip^ is the most stable of the series (107.4 kcal mol^−1^) and is about 60 kcal mol^−1^ more strongly bonded than Mg-cAAC^Dip^ (42.7 kcal mol^−1^). Comparing the homolytic with the heterolytic bond dissociation energies (EL_2_ → E(+2) + 2L(−1), Table S5†), the ionic fragments are much less favoured than the neutral ones in all cases, in agreement with the donor–acceptor picture shown in Scheme 1B. Note that this holds true for Mg-cAAC^Dip^ even when the OSS solution lies 11.4 kcal mol^−1^ below the closed-shell one.

Further insight into the bonding situation is often obtained by means of EDA^31,32^ calculations in conjunction with the NOCV (Natural Orbitals for Chemical Valence) method on the ground state (often BS) KS-DFT descriptions.^33–35^ Details about the method and recent examples have been reported elsewhere,^36^ with a discussion of the nature of the energy components.^37,38^ The method allows the preselection of the electronic structure description outlined as donor–acceptor with E(0) (Scheme 1B) or diradical(oid) with E(+2) (Scheme 1C), using fragment reference states. The best representation is assumed to be the one that provides the lowest orbital relaxation, measured by using the lowest absolute orbital term values. To illustrate this, we have computed the EDAs for the Be-cAAC^Dip^ and Mg-cAAC^Dip^ systems. The values are summarized in Table S8.† The principal bonding picture that emerges from EDA analysis would feature E(0) with two neutral cAAC ligands. The orbital energy terms are −231.4 and −193.0 kcal mol^−1^, for Be-cAAC^Dip^ and Mg-cAAC^Dip^, respectively. Compared with the E(+2) situation, the orbital relaxation leads to higher orbital interactions, −443.8 and −289.8 kcal mol^−1^, for Be-cAAC^Dip^ and Mg-cAAC^Dip^, respectively.

Such energy-based assignation is in contrast with the valence state derived from the effective oxidation state analysis (*vide infra*), and also from that previously suggested by Ponec *et al.*^39^ Both pictures can be reconciled by focusing on the electron flow associated with the orbital interactions rather than focusing on the energy costs. Indeed, the EDA-NOCV approach provides this information as the eigenvalues of the deformation densities. In the π interaction channel, using Be(0) in the ^1^D reference state (Fig. S40†), 0.75α and 0.74β electrons are transferred from the starting electron pair of the Be p_*z*_ orbital to the π-type symmetry ligand orbital. On the contrary, using the Be(+2) reference (Fig. S41†), the electron flow from the ligands to the empty Be p_*z*_ orbital is just 0.20α and 0.22β electrons. Note that the final result is similar in both cases: one ends with 0.49e and the other with 0.42e on the Be p_*z*_ orbital. However, the latter fragmentation leads to an overall smaller electron flow. Thus, one may argue that the reference state for which a smaller electron flow among fragments is found, is the most appropriate reference state. However, this is in contrast with the accepted criterion of choosing the reference states according to the minimum deformation energy required to form a molecule.^2,13,41^ Hence the dichotomy is: should we use the energy or density criterion? one should recall that in the (revised) definition of the oxidation state from the IUPAC there is no mention of energetics, but it is essentially an electron counting problem based on “winner-takes-it-all” principle.^42^

Then, why does a smaller electron flow associated with the Be p_*z*_ orbital have a more significant energy cost? The reason can be inferred again from the NOCV analysis. While the aforementioned electron flow to the Be p_*z*_ empty orbital is just 0.20α and 0.22β electrons, the total electron displacement of this channel is *ca.* 1.8*e*. Therefore, over 75% of the electron flow is associated with the internal reorganization of the fragment density, which certainly has an important energetic impact, but has no influence on the oxidation state.

This conundrum adds up to another related issue of EDA that some of us have recently exposed: EDA cannot distinguish an electron-sharing interaction from a spin-polarized one (diradicaloid).^26^ This problem pops out whenever the closed-shell solution is unstable, which is precisely the case for most systems considered here. For all these reasons, we do not consider the combination of KS-DFT and the energy-based EDA criterion as a reliable approach to ascertain the proper valence state of Be and Mg in these systems.

Alternatively, we resort to multireference CASSCF wave functions to tackle the electronic structure of these systems. This permits to consider the bonding situation for all systems on equal footing (*i.e.*, including those with CSS and OSS ground-states according to KS-DFT methods). The results obtained for all species for their KS-DFT optimized structures are shown in Table 1. Notably, CASSCF wave functions on SS-CASPT2 optimized geometries for the smallest systems E-NHC^Me^ and E-cAAC^Me^ show no significant differences from CASSCF wave functions calculated on DFT geometries, validating the CASSCF//DFT approach used for the largest compounds (see details in the ESI†). The Δ*E*_S–T_ values obtained at CASSCF and CASPT2 levels of theory are in rather good agreement with those obtained with the B3LYP method (see Fig. 1). However, for Be–NHC^Me^, CASPT2 predicts the triplet state to be more stable than the singlet state, by −1.3 kcal mol^−1^, while DFT and CASSCF estimate the triplet state about 5 kcal mol^−1^ above the singlet.

**Fig. 2:**
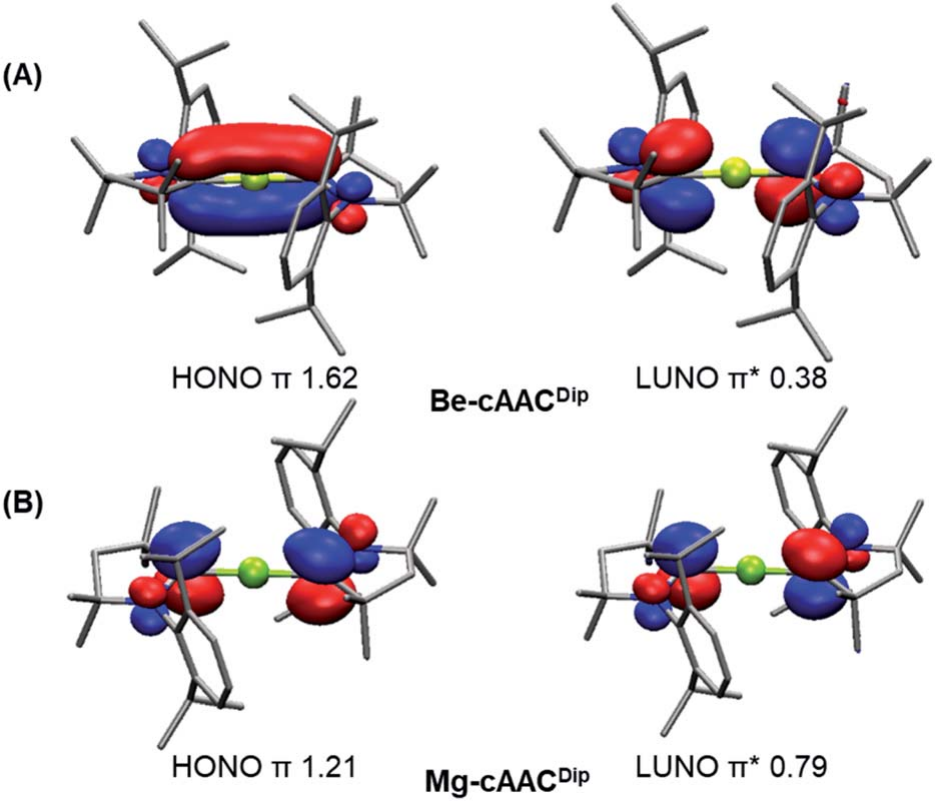
Frontier natural orbitals and occupancies for the Be-cAAC^Dip^ (A) and Mg-cAAC^Dip^ (B) systems at CASSCF/cc-pVDZ//B3LYP-D3(BJ)/def2-SVP. Isocontour value of 0.05. Hydrogen atoms were omitted for clarity.

The CASSCF natural orbitals (NOs) and their occupations in EL_2_ complexes already hint about the bonding situation (Figures S1–S24). These complexes with an acute bond angle present the HONO (Highest Occupied Natural Orbital) and LUNO (Lowest Unoccupied Natural Orbital) localized at the E atom with marked s-type and p-type character, respectively; in agreement with its low partial charge (*vide infra*). Instead, the frontier NOs of the linear complexes resemble the allyl π-system. Thus, the HONO is described as a π-system with in-phase combination π-(+,+,+), while the LUNO is the out-of-phase combination of the extremes π*-(+,•,−) of the C–E–C p-type orbital lobes. The NO corresponding to the π-(+,−,+) combination has negligible occupation. Fig. 2 depicts the orbitals of Be-cAAC^Dip^ and Mg-cAAC^Dip^ species. The occupancies reveal that the HONO has significantly less than two π-electrons, namely 1.62e (Be-cAAC^Dip^) and 1.21e (Mg-cAAC^Dip^). Note that Mg has a weak contribution in the HONO as a consequence of the poor overlap, which also justifies the geometry change throughout the series. In addition, the LUNO carries a significant occupation, *i.e.* 0.38e (Be-cAAC^Dip^) and 0.79e (Mg-cAAC^Dip^). The LUNO occupation varies in the range of 0.13e to 0.38e in the series of BeL_2_.

Different population analyses lead to contradictory results for the partial charge of the E centre (see Tables S9 and S10†). Classical Mulliken or Löwdin schemes yield a quite substantial negative charge on E, which is rather odd considering that they are less electronegative than the C atom. On the contrary, NPA and real-space methods such as QTAIM or TFVC lead to positive charges on E, over +1*e* in the case of Be. These results confirm our above hypothesis that π electrons are much more localized at the ligands than the metal centre. Note that the more π-acidic the character of the ligand, the higher the (positive) partial charge on E. As a consequence, the complexes can also exhibit a different degree of diradical character.

On the other hand, the E–C bond orders (BO) for the NHC- and cAAC-substituted systems indicate the binding degree of the ligand to the central element. In particular, the obtained BO values range from 0.25 (Mg-NHC^Dip^) to 0.56 (Be-cAAC^Me^). These values are well complemented with the corresponding dissociation energies (especially for the Mg-based compounds) and also with the occupation of the HONO (the more it deviates from 2, the smaller the BO).

Noteworthily, Ponec *et al.* also analysed a small collinear model Be-cAAC system in light of the domain-averaged Fermi Hole (DAFH) analysis at the CASSCF level of theory.^39^ By dissecting the σ and π bonding between the fragments, they found evidence for a 3c-2e π bond involving both ligands and Be, with contributions of 0.95e from each ligand and 0.14e from Be. That is, the contribution of Be to the π bonding is residual, which puts into question the alleged Be(0) valence state of these systems according to the authors.

In this context, the cAAC-substituted compounds could be better interpreted as diradical(oid)s species. The global diradical(oid) character is typically quantified from the occupation numbers of the NOs.^43,44^ However, in some systems several NOs with significant occupations are involved, so the usual underlying 2c-2e model is insufficient to describe the diradical character.

Instead, we have used the local spin analysis (LSA), which quantifies the presence of local spin on atoms/fragments and their couplings from correlated wavefunctions even in the singlet state (*i.e.* with no spin density) (see Table 1). In LSA, the <S^2^> value is dissected in atomic and diatomic contributions, which can be further grouped into fragment contributions (*i.e.* Mg/Be atom and each of the two ligands).^45^ For the NHC-coordinated systems, both the <S^2^>_NHC_ and <S^2^>_E_ values are below 0.15 in all cases except Be-NHC^Dip^ (<S^2^>_NHC_ = 0.22). Interestingly, in the cAAC-based compounds the <S^2^>_cAAC_ values increase from 0.10 (Mg-cAAC^Me^) to 0.68 (Mg-cAAC^Dip^), and from 0.30 (Be-cAAC^Me^) to 0.39 (Be-cAAC^Dip^). The < S^2^>_E_ values also remain below 0.12 in all cases, ruling out the presence of unpaired electrons in the central atom. The bonding picture thus points towards two antiferromagnetically coupled unpaired spins, each one located at the π-system of the cAAC ligand. This coupling is supported by the <S^2^>_cAAC-cAAC_ values (see the ESI† for details), being −0.64 (very close to the ideal value −0.75, see the ESI†) for Mg-cAAC^Dip^. In the case of the experimentally known Be-cAAC^Dip^, the <S^2^>_cAAC_ and <S^2^>_cAAC-cAAC_ values are 0.39 and −0.35, respectively, indicating the marked diradical character. In the diradical(oid) scenario, the valence state of the E atom would be E(+2).

A more unambiguous look at the formal valence state or OS of the E centre and the ligands is given by the effective oxidation state (EOS) analysis,^46^ a wavefunction analysis tool specifically devised for this purpose. EOS analysis relies on Mayer's effective fragment orbitals (EFOs) and their occupations, obtained in this case for the E atom and each of the two ligands.^47,48^ The EFOs are sorted by decreasing occupation number and individual electrons (electron pairs for closed-shell systems) are assigned to them until the total number of electrons is reached. The last occupied and first unoccupied orbitals form the frontier EFOs, and from their relative occupations a reliability index (*R*) can be derived, measuring to which extent the formal OS model matches the actual electron distribution (for further details see the ESI†).

**Fig. 3:**
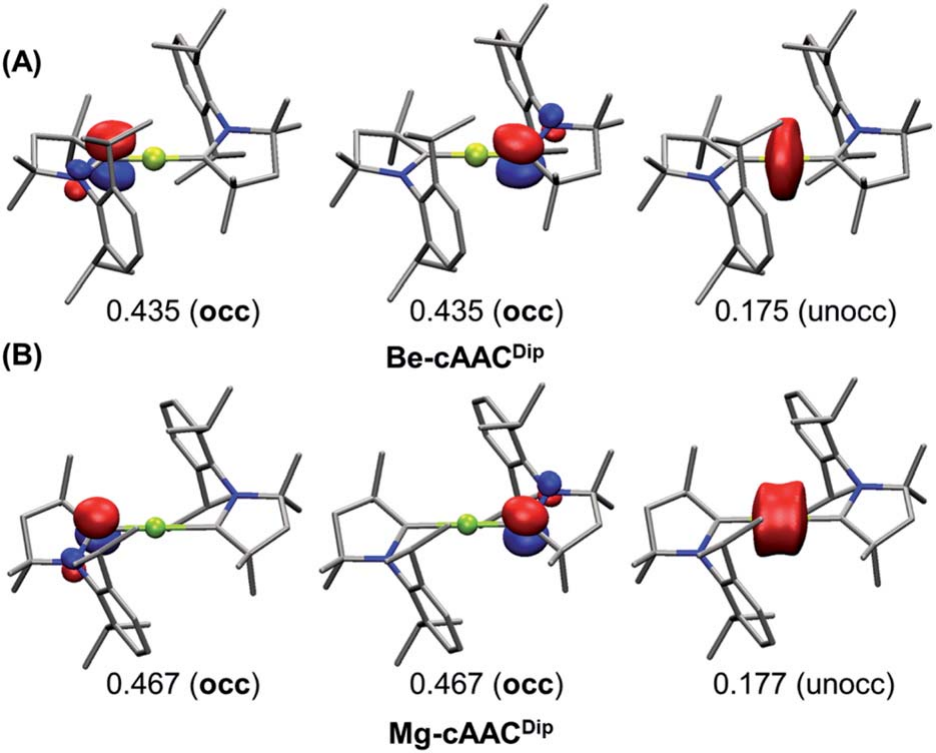
Frontier EFOs with their corresponding gross occupancies for the Be-cAAC^Dip^ (A) and Mg-cAAC^Dip^ (B) systems (singlet spin state) obtained at the CASSCF/cc-pVDZ//B3LYP-D3(BJ)/def2-SVP level. Isocontour value: 0.1. Hydrogen atoms were omitted for clarity.

The results of EOS analysis applied to the ground-state CASSCF wavefunctions are also shown in Table 1. For comparison, we have included BeH_2_ (Be–H) and MgH_2_ (Mg–H) as genuine E(+2) species. The real-space TFVC atomic definition was used throughout. In the case of near collinear systems (C–E–C angle >160°), the OS of the central E is +2, in line with the discussion above and also in agreement with the study by Ponec *et al.*^39^Fig. 3 illustrates the situation. The last occupied EFOs of Be-cAAC^Dip^ and Mg-cAAC^Dip^ correspond to π-type orbitals located on the cAAC ligands with occupancies of 0.435 and 0.467, respectively. When frontier EFOs are degenerated in occupation, EOS analysis advocates for homolytic assignation of the last electron pair, leading to the formal picture shown in Scheme 1C. In any case, the occupation of the last unoccupied EFO on E is so small (see Fig. 3) that the E(+2) assignation is unambiguous.

On the contrary, in the bent Mg system, the EOS scheme clearly points towards a genuine Mg(0) compound, even for a system like Mg-cAAC^Me^ with incipient diradical character.

It is fair to note that EOS analysis can be applied using different underlying atomic definitions, which can impact the occupations of the EFOs and therefore the OS assignation itself. Indeed, using EOS in the framework of Mulliken or Löwdin analyses leads to Be(0) assignment in Be–NHC^Me^ and Be-cAAC^Me^ systems (see Table S9†). However, more reliable NAO or QTAIM schemes yield essentially the same results as those reported in Table 1. Still, the EOS procedure is shown to be much more robust than the partial atomic charges. Note, for instance, the unambiguous Mg(0) picture obtained for Mg–NHC^Me^ or Mg-cAAC^Me^ systems across all atomic definitions, while the partial atomic charge in Mg varies from −0.75e to +0.60e. We have also applied EOS on the B3LYP ground-state description of these systems (see Table S10†). Other than the particular case of Be–NHC^Me^ in combination with Löwdin analysis, which again yields Be(0), the OS assignation is fully consistent with that derived from CASSCF wavefunctions.

So far we have consistently shown that the equilibrium structures of some of these systems exhibit diradical character and are best described as E(+2). The dissociation energies, however, clearly point towards the homolytic dissociation into E(0) + 2L(0) (see Fig. 1). This situation is reminiscent of the simplest LiH diatomic molecule, where at the equilibrium bond distance the best description is Li(+1)/H(−1) but the dissociation is homolytic.

**Fig. 4:**
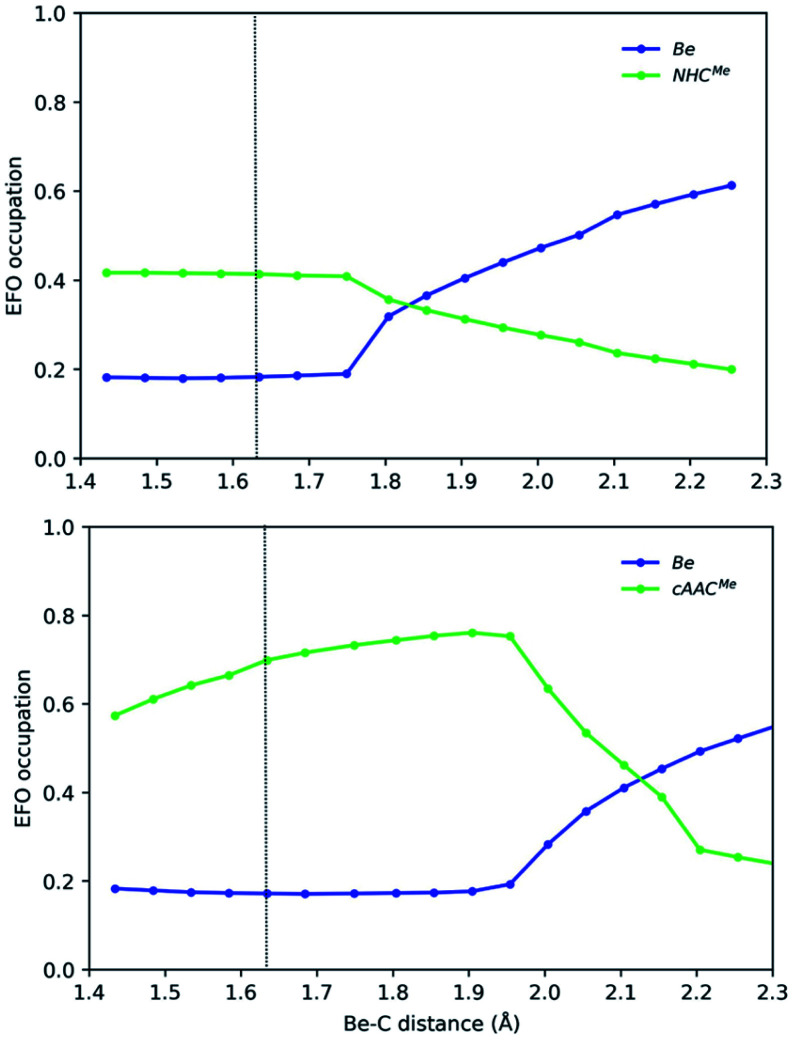
Gross occupations of the frontier EFOs for Be-NHC^Me^ (top) and Be-cAAC^Me^ (bottom) along the Be–C distance at the B3LYP-D3(BJ)/def2-TZVPP//B3LYP-D3(BJ)/def2-SVP level of theory. The dotted line indicates the equilibrium structure.

**Fig. 5:**
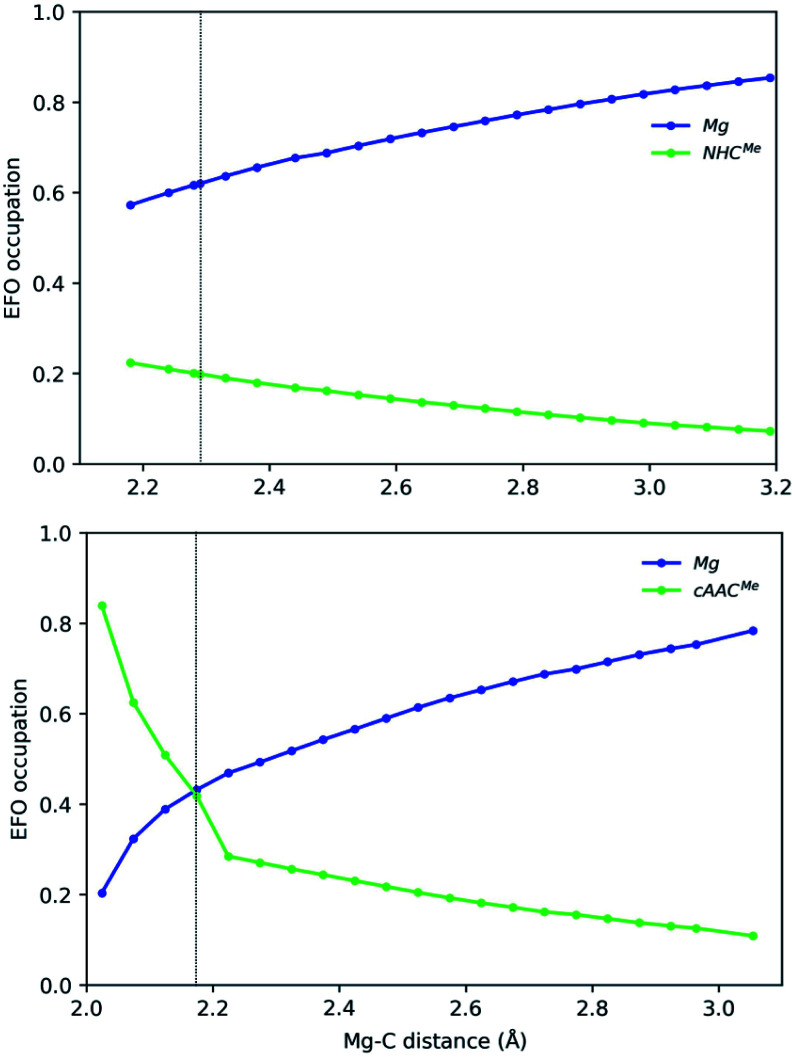
Gross occupations of the frontier EFOs for Mg-NHC^Me^ (top) and Mg-cAAC^Me^ (bottom) along the Mg–C distance at the B3LYP-D3(BJ)/def2-TZVPP//B3LYP-D3(BJ)/def2-SVP level of theory. The dotted line indicates the equilibrium structure.

We have thus monitored the OS of representative EL_2_ species along the symmetric E–L dissociation profile. Fig. 4 and 5 show the gross occupation of the frontier EFOs of E and L with the increase of the E–L distance. In the case of Be–NHC^Me^, the coordination is essentially collinear at equilibrium. Therefore, the EFO occupation of the ligand is higher than that of Be, leading to a Be(+2) picture. As the Be–C distance increases, the occupation of the ligand's EFO gradually decreases, while that of Be increases. The lowest energy dissociation profile proceeds first in a collinear configuration until a Be–C distance of *ca* 1.7 Å, when the C–Be–C angle bends so that the Be atom dissociates perpendicularly to the interatomic C–C axis. From this point on, the occupation of Be 2s-type EFO rapidly increases and the change of formal OS from Be(+2) to Be(0) occurs at a Be–C distance of *ca.* 1.85 Å.

In Be-cAAC^Me^, the BS solution is lower in energy at the equilibrium geometry. The spin polarization is mostly notorious on the ligands' frontier EFOs, which have α and β occupations much larger than those of the Be centre. The dissociation starts in a collinear fashion. The diradical character increases with the Be–C distance, reaching its maximum (<S^2^> = 0.60) at *ca.* 1.95 Å, thus resulting in a much marked Be(+2) character. Beyond this point, the C–Be–C angle bends so that once again the Be atom dissociates perpendicularly to the interatomic C–C axis. In the process the diradical character rapidly decreases, inducing a charge transfer from the ligands to Be's 2s-type EFO until the formal OS changes at around 2.1 Å, where the open-shell solution merges into the closed-shell one. This again shows that a stable angular geometry is a key to achieving genuine low valent species.

In the case of the Mg-based species, the equilibrium structure already points towards a Mg(0) species, so that the increase of the Mg–C distance further increases monotonically the gap in the EFO occupations in favour of the Mg moiety in closed-shell dissociation profiles (see Fig. 5). Coincidentally, in Mg-cAAC^Me^, the ground state is still of OSS nature after spin-contamination correction, and the occupations of Mg and cAAC frontier EFOs are almost equal. When using the CASSCF wavefunction at this geometry, the situation is more clear, resulting in an Mg(0) description with *R*(%) = 80 as shown in Table 1.

**Fig. 6:**
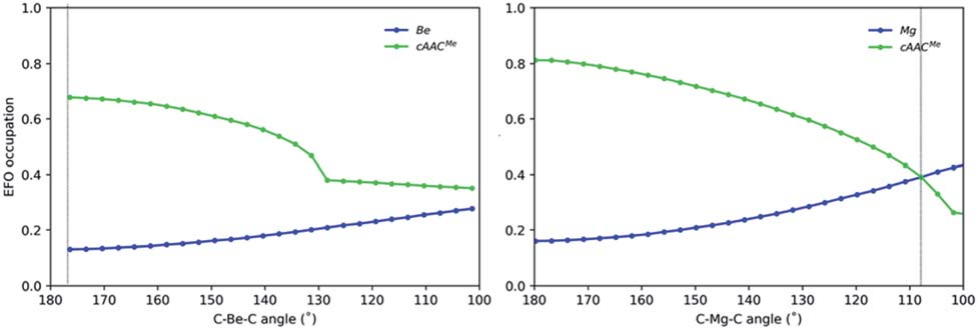
Gross occupations of the frontier EFOs for Be-cAAC^Me^ and Mg-cAAC^Me^ along the C–E–C angle at the B3LYP-D3(BJ)/def2-TZVPP//B3LYP-D3(BJ)/def2-SVP level of theory. The dotted line indicates the equilibrium structure.

Since the OS of the central E atom is clearly influenced by the C–E–C angle, we also analysed the EOS performance along the C–E–C bond angle for Be-cAAC^Me^ and Mg-cAAC^Me^ species at the B3LYP level of theory (Fig. 6). The occupation of the frontier EFO on E monotonically increases as the C–E–C angle deviates from collinearity. In the case of Be, the EFO occupation remains always below 0.3 and that of the ligand remains always larger, even for closed C–Be–C angles (up to *ca.* 130°) where the CS solution prevails. However, in the case of Mg-cAAC^Me^ one can observe a crossing point at around 110° where the occupation of the Mg EFO becomes large enough to yield a Mg(0) picture. This occurs even before the closed-shell regime is reached, and in line with the CASSCF results.

At this point we can safely state that the +2 oxidation state is a consequence of the strong electron π-accepting properties of the ligands. It is interesting to contrast this analysis with the strong σ-donor and weak π-acceptor monoanionic β-diketiminate magnesium ligands. Optimizing the experimentally accomplished Mg-BDI* leads to an almost collinear structure with an Mg–Mg–Mg angle of 175.4°. Structures resulting from reducing the steric encumber hold the same structural features with a bond angle of 180°. However, the bond lengths are sharply increased from 2.800 Å (Mg-BDI*) to 2.917 Å (Mg-NacNac). No open-shell singlet solutions were found for these compounds, and large vertical singlet–triplet gaps were obtained at both DFT and CASSCF levels (>25 kcal mol^−1^). As expected, the ligand interaction with the central element is explained by the σ-type natural orbital (NO) with an occupancy close to 1.90 (see Fig. S17 and S19†). The p- and π*-type natural orbitals present occupancies lower than 0.1, a fingerprint of dynamic correlation. In the triplet state, one electron from the σ-type NO needs to be transferred to a π-type NO from the central element. Besides, the bond dissociation energies 49.0 and 63.9 kcal mol^−1^ suggest stable compounds for NacNac and BDI* derivatives, respectively, in agreement with the large Mg–Mg BOs (0.76 and 0.52). Note that Mg-BDI* has a lower BO despite the higher dissociation energy, due to the dispersion interaction between the ligands. In line with these findings, EOS analysis also yields a relatively straightforward Mg(0) assignation (see Table S4 and Fig. S38†).

This concept can be used to take beryllium to even lower oxidation states. Be-NacNac and Be-BDI* are predicted to be stable towards the dissociation, with 70.0 and 83.9 kcal mol^−1^, respectively. The description of the electronic structures shows no appreciable diradical character, with singlet–triplet gaps of 30.2 and 35.7 kcal mol^−1^. Given the higher electronegativity of Be with respect to Mg, the partial charges at Be are strongly negative −0.90 and −0.42 au. Formally, these molecules bear a beryllium atom with an oxidation state of −2, which is further corroborated by EOS analysis (see Table S4 and Fig. S39†).

## Conclusions

In summary, we re-examine the features of the structure, chemical bonding, and stability of the low-valent group 2 compounds. In contrast to the accepted understanding, beryllium still remains in the +2 oxidation state territory. The strong σ-donor stabilized approach produces an internal electronic rearrangement furnishing diradical(oid) species with two unpaired electrons on the ligands. Magnesium analogues might present oxidation state zero when the ligands are not too π-acidic, but the chemical bond is too weak to consider these molecules thermally stable. Nonetheless, the effective oxidation state analysis suggests that the strongly Mg-based ligands are key to accessing genuine low-valent compounds. Our study does not only give more insight into the peculiar features of the molecules considered, but also suggest a promising novel type of beryllium −2 oxidation state. The presented results indicate that many more are yet to come to the fore from these combinations.

## Data availability

Data available in article ESI.†

## Author contributions

M. G., S. D., E. V., and C. Y. performed the calculations. A. J., I. C., P. S., and D. M. A. acquired funding and contributed methodologies. M. G., S. D. P. S., and D. M. A. prepared the manuscript and the ESI.† All authors contributed to the interpretation of the computed data and the writing and editing of the manuscript.

## Conflicts of interest

There are no conflicts to declare.

## Supplementary Material

SC-013-D2SC01401G-s001
