# Supplementary material for: The oxidation state in low-valent beryllium and magnesium compounds
Source: Chem Sci. 2022 May 9;13(22):6583–91. doi: 10.1039/d2sc01401g (PMC9172369; doi:10.1039/d2sc01401g)
Supplement: SC-013-D2SC01401G-s001 [file SC-013-D2SC01401G-s001.pdf]

Electronic Supplementary Material (ESI) for Chemical Science.
This journal is © The Royal Society of Chemistry 2022

Supporting Information

for

The Oxidation State in Low-Valent Beryllium and Magnesium Compounds

Martí Gimferrer,[a],+ Sergi Danés,[b],+ Eva Vos,[c] Cem B. Yildiz,[d] Inés Corral,*,[c] Anukul Jana,*,[e] Pedro Salvador,*,[a] and Diego M. Andrada*,[b]

[a] Institut de Química Computacional i Catàlisi, Departament de Química, Universitat de Girona, c/ M. Aurelia Capmany 69,17003, Girona, Spain.

[b] General and Inorganic Chemistry Department, University of Saarland, Campus C4.1, 66123 Saarbruecken, Germany.

[c] Departamento de Química, Universidad Autónoma de Madrid, C/ Francisco Tomus y Valiente 7, 28049 Cantoblanco Madrid, Spain.

[d] Department of Medicinal and Aromatic Plants, Aksaray University, Hacilar harmani 2, 68100 Aksaray, Turkey.

[e] Tata Institute of Fundamental Research Hyderabad, Gopanpally, Hyderabad-500046, Telangana, India.

Table of Contents

Computational Details……………………………………………………………………………………S2-S3
Table S1. KS-DFT ground-state multiplicities and singlet-triplet gaps of the systems studied ……………………S4
Table S2 and S3. KS-DFT relative energies (spin-corrected and not) of the systems studied ………………S5-S6
Table S4. Geometric and Electronic parameters for the singlet and triplet spin states at the B3LYP/def2-TZVPP level ……………………………………………………………………………………S7
Table S5-S7. Dissociation energies using different fragmentation patterns……………………………S8-S9
Figures S1-S24. Natural orbitals shapes and occupancies of the systems studied (CASSCF)………………S10-S33
Figures S25-S34. Molecular orbitals (CASSCF) and most relevant CI vector values ……………………S34-S43
Figures S35-S39. Most relevant effective fragment orbitals and occupancies ……………………………S44-S48
Table S8. EDA-NOCV results ……………………………………………………………………………S49
Figures S40-S43. Deformation densities……………………………………………………………S50-S53
Tables S9-S10. Central element atomic charges and EOS results from benchmarked atomic definitions...S54-S55
Table S11. Geometric parameters and relative energies for the singlet and triplet spin states at the SS-CASPT2/cc-pVDZ level……………………………………………………………………………S56
Figures S44-S51. Molecular orbitals (CASSCF) occupancies and the most relevant CI vector values of the systems studied at SS-CASPT2……………………………………………………………………S57-S64
Figures S52. Potential energy surfaces (dissociation of the E-C bond) of **Be-cAAC^{Me}**, **Be-NHC^{Me}**, **Mg-cAAC^{Me}** and **Mg-NHC^{Me}**……………………………………………………………………………S65
Figures S53. Potential energy surfaces (E-C-E bending) of **Be-cAAC^{Me}** and **Mg-cAAC^{Me}** ……………………S65
References……………………………………………………………………………………………S66

Computational Details

Benchmark KS-DFT calculations

We evaluated both geometries and electronic energies of all species using a series of KS-DFT functionals, namely BP86,[1] B3LYP,[2] PBE0,[3] M06-2X,[4] and wB97xD.[5] The geometries have been evaluated at the KS-DFT/def2-SVP level, meanwhile electronic energies were evaluated using the def2-TZVPP basis set.[6] Dispersion corrections were included with the Grimme formalism D3,[7] and the Becke-Jonson damping function, when possible.[8] The stationary points were located with the Berny algorithm[9] using redundant internal coordinates. Analytical Hessians were computed to determine the nature of stationary points.[10] For the singlet open-shell solutions, we corrected the energies by using the (vertical) triplet spin-state solution and applying the spin-contamination correction proposed by Yamaguchi.[11] The ground-state and the adiabatic singlet-triplet gaps obtained at each level are compiled in Table S1. Relative energies of all species at each stable spin-state multiplicity compiled in Tables S2-S3. All the aforementioned calculations have been performed with the Gaussian16.C01 package.[12]

CASSCF calculations

We performed single-point multiconfigurational calculations by the means of Complete Active Space Self-Consistent Field (CASSCF) method in combination to the correlated consistent double-zeta basis set cc-pVDZ.[13] The geometries used were obtained at the B3LYP-D3(BJ)/def2-SVP level of theory. The chemically relevant orbitals have been selected and included within the active space. In particular, the active space size consists on10 electrons distributed within 10 active orbitals for the NHC-substituted compounds, or 10 electrons within 8 active orbitals for the cAAC-substituted compounds, or 4 electrons within 8 active orbitals for the NacNac-substituted ones, or 4 electrons within 10 active orbitals for **BeH_2** and **MgH_2**. The shape and symmetry of the active-space natural orbitals, together with its non-integer occupation, can be found in Figures S1-S17. The CASSCF calculations has been performed with the pySCF1.7 package.[14]

Chemical bonding descriptors calculations

Atom/fragment charges, bond orders, spin-resolved effective fragment orbitals (EFOs), effective oxidation states (EOS) and local spin analysis (LSA) have been obtained with the APOST-3D program,[15] using the topological fuzzy voronoi cells (TFVC) 3D-space atomic definition and a 40 x 146 atomic grid for numerical integration.
The energy decomposition analysis (EDA) with the natural orbitals for chemical valence (NOCV) calculations on the optimized B3LYP-D3(BJ)/def2-SVP geometries were carried out with ADF 2019.103.[16] The basis sets for all elements have triple-ζ quality augmented by two sets of polarizations functions. The level of theory used were B3LYP-D3(BJ)/TZ2P.[17] Scalar relativistic effects have been incorporated by applying the zeroth-order regular approximation (ZORA).[18]

SS-CASPT2 calculations

For the smallest systems (**Mg-NHC^{Me}**, **Mg-cAAC^{Me}**, **Be-NHC^{Me}** and **Be-cAAC^{Me}**), we have performed SS-CASPT2[19,20]/cc-pVDZ geometry optimizations using the cc-pVDZ-jkfit auxiliar basis set. The active spaces employed were the same as the ones used for the CASSCF//B3LYP-D3(BJ) calculations: (10,10) for the systems carrying NHC^{Me} ligands and (10,8) for the one with cAAC^{Me} ligands. The natural orbitals included in the active space with the corresponding occupation numbers and the wavefunction CI coefficients are collected in Figures S44-S51. All the SS-CASPT2 calculations were carried out using BAGEL program[21].

Local Spin Analysis (LSA)

Let us analyze in the light of LSA a collinear system of three fragments depicted in Scheme S1. In the case of a singlet spin-state having one electron perfectly localized on each ligand, we would expect local spin values of $<S^2>_f$ = 3/4 for each fragment and $<S^2>_f$ = 0 for the central moiety. The fact that the spins are anti-ferromagnetically coupled is captured by the inter-fragment spin-coupling contribution of $<S^2>_{f-f'}$ =-3/4. The same situation in triplet state would lead to the same local spin values, but in this case the inter-fragment contribution would amount $<S^2>_{f-f'}$ =+1/4, indicating parallel alignment of the spins (see Scheme S1b).

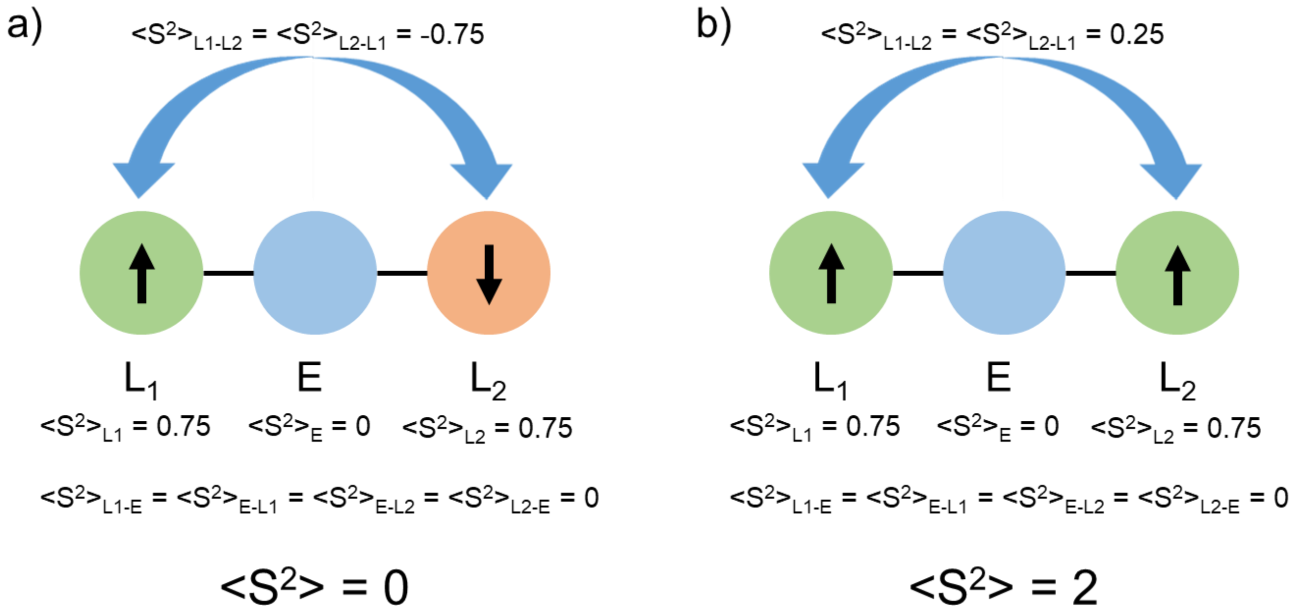

Scheme S1. Representation of the E-L_2 system local spin decomposition in an ideal singlet (a) and triplet (b) state diradical(oid)s with unpaired spins localized on the L fragments.

Table S1. Ground state multiplicity and adiabatic (spin-corrected) singlet-triplet (S-T) gap obtained using the selected KS-DFT functionals.

	Ground State Multiplicity					ΔE_{S-T} Gap (kcal/mol)				
System	BP86	B3LYP	PBE0	M06-2X	wB97xD	BP86	B3LYP	PBE0	M06-2X	wB97xD
Mg-NHC^{Me}	CSS	CSS	CSS	CSS	CSS	8.9	10.9	7.8	7.5	12.1
Mg-NHC^{Dip}	CSS	CSS	CSS	CSS	CSS	9.8	13.3	11.1	0.9	14.2
Mg-cAAC^{Me}	CSS	OSS	OSS	T	OSS	5.1	3.3	3.2	-0.9	2.5
Mg-cAAC^{Dip}	OSS	OSS	OSS	T	OSS	3.7	2.4	2.2	-0.7	1.5
Be-NHC^{Me}	CSS	CSS	CSS	T	CSS	4.7	4.6	2.0	-3.4	2.1
Be-NHC^{Dip}	CSS	CSS	OSS	T	OSS	9.8	7.9	5.6	-5.5	9.0
Be-cAAC^{Me}	CSS	OSS	OSS	OSS	OSS	6.7	6.4	5.4	1.3	6.6
Be-cAAC^{Dip}	CSS	OSS	OSS	OSS	OSS	8.4	8.5	8.1	4.0	6.9
Be-NacNac^{Me}	CSS	CSS	CSS	CSS	CSS	27.1[b]	30.2[b]	29.5[b]	33.8[b]	30.5[b]
Be-BDI*	CSS	CSS	CSS	CSS	CSS	31.8[b]	35.7[b]	32.5[b]	29.3[b]	34.7[b]
Mg-NacNac^{Me}	CSS	CSS	CSS	CSS	CSS	31.0[b]	38.3[b]	34.7[b]	37.6[b]	36.6[b]
Mg-BDI*	CSS	CSS	CSS	CSS	CSS	32.6[b]	39.2[b]	36.2[b]	47.8[b]	41.0[b]

[a] CSS = closed-shell singlet, OSS = open-shell singlet, T = Triplet. [b] vertical singlet-triplet gap.

Table S2. Relative spin-corrected electronic energies for the CSS, OSS and T spin-states obtained using the selected KS-DFT functionals.

	Relative energies to the ground-state multiplicity (kcal/mol)				
System	BP86	B3LYP	PBE0	M06-2X	wB97xD
Mg-NHC^{Me}	0.0 (CSS)<br>8.9 (T)	0.0 (CSS)<br>10.9 (T)	0.0 (CSS)<br>7.8 (T)	0.0 (CSS)<br>7.5 (T)	0.0 (CSS)<br>12.1 (T)
Mg-NHC^{Dip}	0.0 (CSS)<br>9.8 (T)	0.0 (CSS)<br>13.3 (T)	0.0 (CSS)<br>11.1 (T)	0.0 (CSS)<br>0.9 (T)	0.0 (CSS)<br>14.2 (T)
Mg-cAAC^{Me}	0.0 (CSS)<br>5.1 (T)	0.9 (CSS)<br>0.0 (OSS)<br>3.3 (T)	3.1 (CSS)<br>0.0 (OSS)<br>3.2 (T)	1.9 (CSS)<br>0.0 (OSS)<br>-0.9 (T)	5.6 (CSS)<br>0.0 (OSS)<br>2.5 (T)
Mg-cAAC^{Dip}	3.7 (CSS)<br>0.0 (OSS)<br>3.7 (T)	11.4 (CSS)<br>0.0 (OSS)<br>2.4 (T)	14.0 (CSS)<br>0.0 (OSS)<br>2.2 (T)	15.6 (CSS)<br>0.0 (OSS)<br>-0.7 (T)	22.0 (CSS)<br>0.0 (OSS)<br>1.5 (T)
Be-NHC^{Me}	0.0 (CSS)<br>4.7 (T)	0.0 (CSS)<br>4.6 (T)	0.0 (CSS)<br>2.0 (T)	0.0 (CSS)<br>-3.4 (T)	0.0 (CSS)<br>2.1 (T)
Be-NHC^{Dip}	0.0 (CSS)<br>9.8 (T)	0.0 (CSS)<br>7.9 (T)	0.4 (CSS)<br>0.0 (OSS)<br>5.6 (T)	2.2 (CSS)<br>0.0 (OSS)<br>-5.5 (T)	5.5 (CSS)<br>0.0 (OSS)<br>9.0 (T)
Be-cAAC^{Me}	0.0 (CSS)<br>6.7 (T)	3.8 (CSS)<br>0.0 (OSS)<br>6.4 (T)	5.7 (CSS)<br>0.0 (OSS)<br>5.4 (T)	6.3 (CSS)<br>0.0 (OSS)<br>1.3 (T)	9.9 (CSS)<br>0.0 (OSS)<br>6.6 (T)
Be-cAAC^{Dip}	0.0 (CSS)<br>8.4 (T)	5.2 (CSS)<br>0.0 (OSS)<br>8.5 (T)	7.1 (CSS)<br>0.0 (OSS)<br>8.1 (T)	8.3 (CSS)<br>0.0 (OSS)<br>4.0 (T)	11.5 (CSS)<br>0.0 (OSS)<br>6.9 (T)
Be-NacNac^{Me}[b]	0.0 (CSS)<br>27.1 (T)	0.0 (CSS)<br>30.2 (T)	0.0 (CSS)<br>29.5 (T)	0.0 (CSS)<br>33.8 (T)	0.0 (CSS)<br>30.5 (T)
Be-BDI*[b]	0.0 (CSS)<br>31.8 (T)	0.0 (CSS)<br>35.7 (T)	0.0 (CSS)<br>32.5 (T)	0.0 (CSS)<br>29.3 (T)	0.0 (CSS)<br>34.7 (T)
Mg-NacNac^{Me}[b]	0.0 (CSS)<br>31.0 (T)	0.0 (CSS)<br>38.3 (T)	0.0 (CSS)<br>34.7 (T)	0.0 (CSS)<br>37.6 (T)	0.0 (CSS)<br>36.6 (T)
Mg-BDI*[b]	0.0 (CSS)<br>32.6 (T)	0.0 (CSS)<br>39.2 (T)	0.0 (CSS)<br>36.2 (T)	0.0 (CSS)<br>47.8 (T)	0.0 (CSS)<br>41.0 (T)

[a] CSS = closed-shell singlet, OSS = open-shell singlet, T = Triplet. [b] vertical singlet-triplet gap.

Table S3. Relative spin-uncorrected electronic energies for the CSS, OSS and T spin-states obtained using the selected KS-DFT functionals.

	Relative energies to the ground-state multiplicity (kcal/mol)				
System	BP86	B3LYP	PBE0	M06-2X	wB97xD
Mg-NHC^{Me}	0.0 (CSS)<br>8.9 (T)	0.0 (CSS)<br>10.9 (T)	0.0 (CSS)<br>7.8 (T)	0.0 (CSS)<br>7.5 (T)	0.0 (CSS)<br>12.1 (T)
Mg-NHC^{Dip}	0.0 (CSS)<br>9.8 (T)	0.0 (CSS)<br>13.3 (T)	0.0 (CSS)<br>11.1 (T)	0.0 (CSS)<br>0.9 (T)	0.0 (CSS)<br>14.2 (T)
Mg-cAAC^{Me}	0.0 (CSS)<br>5.1 (T)	0.0 (CSS)<br>0.0 (OSS)<br>2.4 (T)	0.6 (CSS)<br>0.0 (OSS)<br>0.7 (T)	1.9 (CSS)<br>0.0 (OSS)<br>-0.9 (T)	3.7 (CSS)<br>0.0 (OSS)<br>0.6 (T)
Mg-cAAC^{Dip}	2.3 (CSS)<br>0.0 (OSS)<br>2.3 (T)	10.3 (CSS)<br>0.0 (OSS)<br>1.3 (T)	13.1 (CSS)<br>0.0 (OSS)<br>1.2 (T)	15.9 (CSS)<br>0.0 (OSS)<br>-0.4 (T)	21.3 (CSS)<br>0.0 (OSS)<br>0.8 (T)
Be-NHC^{Me}	0.0 (CSS)<br>4.7 (T)	0.0 (CSS)<br>4.6 (T)	0.0 (CSS)<br>2.0 (T)	0.0 (CSS)<br>-3.4 (T)	0.0 (CSS)<br>2.1 (T)
Be-NHC^{Dip}	0.0 (CSS)<br>9.8 (T)	0.0 (CSS)<br>7.9 (T)	0.0 (CSS)<br>0.0 (OSS)<br>5.1 (T)	0.9 (CSS)<br>0.0 (OSS)<br>-6.8 (T)	1.7 (CSS)<br>0.0 (OSS)<br>5.1 (T)
Be-cAAC^{Me}	0.0 (CSS)<br>6.7 (T)	0.9 (CSS)<br>0.0 (OSS)<br>3.5 (T)	2.1 (CSS)<br>0.0 (OSS)<br>1.7 (T)	3.5 (CSS)<br>0.0 (OSS)<br>-1.4 (T)	5.1 (CSS)<br>0.0 (OSS)<br>1.8 (T)
Be-cAAC^{Dip}	0.0 (CSS)<br>8.4 (T)	1.8 (CSS)<br>0.0 (OSS)<br>5.1 (T)	3.4 (CSS)<br>0.0 (OSS)<br>4.3 (T)	5.5 (CSS)<br>0.0 (OSS)<br>1.3 (T)	7.8 (CSS)<br>0.0 (OSS)<br>3.2 (T)
Be-NacNac^{Me}[b]	0.0 (CSS)<br>27.1 (T)	0.0 (CSS)<br>30.2 (T)	0.0 (CSS)<br>29.5 (T)	0.0 (CSS)<br>33.8 (T)	0.0 (CSS)<br>30.5 (T)
Be-BDI*[b]	0.0 (CSS)<br>31.8 (T)	0.0 (CSS)<br>35.7 (T)	0.0 (CSS)<br>32.5 (T)	0.0 (CSS)<br>29.3 (T)	0.0 (CSS)<br>34.7 (T)
Mg-NacNac^{Me}[b]	0.0 (CSS)<br>31.0 (T)	0.0 (CSS)<br>38.3 (T)	0.0 (CSS)<br>34.7 (T)	0.0 (CSS)<br>37.6 (T)	0.0 (CSS)<br>36.6 (T)
Mg-BDI*[b]	0.0 (CSS)<br>32.6 (T)	0.0 (CSS)<br>39.2 (T)	0.0 (CSS)<br>36.2 (T)	0.0 (CSS)<br>47.8 (T)	0.0 (CSS)<br>41.0 (T)

[a] CSS = closed-shell singlet, OSS = open-shell singlet, T = Triplet. [b] vertical singlet-triplet gap.

Table S4. Geometrical parameters, central E atomic charges, Q(E), (E = Mg or Be), E-C and E-Mg bond orders ($BO_{E\text{-}L}$), fragment and inter-fragment local spin ($\langle S^2\rangle_f$ and $\langle S^2\rangle_{f1\text{-}f2}$), EOS results and global reliability index (R (%)) of the studied compounds in its ground state multiplicity and triplet state (in parenthesis).[a]

E-L2 system	$d_{E\text{-}L}$ [Å]	$BA_{L\text{-}E\text{-}L}$ [°]	Q(E)	$BO_{E\text{-}L}$	$\langle S^2\rangle_E$	$\langle S^2\rangle_L$	$\langle S^2\rangle_{L1\text{-}L2}$	E OS	L OS	R (%)
Mg-NHC^{Me}	2.300 (2.191)	90.1 (103.4)	0.25 (0.55)	0.27 (0.34)	0.10 (1.01)	0.07 (0.35)	-0.02 (0.02)	0 (0)	0 (0)	100 (56.5)
Mg-NHC^{Dip}	2.347 (2.154)	119.3 (145.8)	0.61 (1.02)	0.25 (0.40)	0.09 (0.44)	0.07 (0.59)	-0.02 (0.11)	0 (+2)	0 (-1)[b]	82.7 (100)
Mg-cAAC^{Me}	2.174 (2.060)	107.8 (169.5)	0.60 (1.28)	0.35 (0.51)	0.12 (0.14)	0.10 (0.72)	-0.04 (0.20)	0 (+2)	0 (-1)	80.0 (100)
Mg-cAAC^{Dip}	2.040 (2.044)	178.9 (179.1)	1.34 (1.36)	0.45 (0.44)	0.08 (0.08)	0.68 (0.76)	-0.64 (0.22)	+2 (+2)	-1 (-1)	82.4 (100)
Mg-NacNac^{Me}	2.917	180.0	0.06	0.76	0.12	0.10	-0.02	0	0	59.0
Mg-BDI*	2.800	175.4	0.37	0.52	-	-	-	0	0	59.9
Be-NHC^{Me}	1.639 (1.649)	179.9 (180.0)	1.10 (1.08)	0.56 (0.54)	0.06 (0.33)	0.14 (0.66)	-0.11 (0.12)	+2 (+2)	-1 (-1)	74.0 (100)
Be-NHC^{Dip}	1.648 (1.658)	167.4 (178.9)	1.12 (1.16)	0.54 (0.52)	0.08 (0.25)	0.22 (0.68)	-0.18 (0.15)	+2 (+2)	-1 (-1)	73.8 (100)
Be-cAAC^{Me}	1.634 (1.644)	176.4 (176.0)	1.17 (1.25)	0.56 (0.53)	0.07 (0.16)	0.30 (0.70)	-0.26 (0.18)	+2 (+2)	-1 (-1)	77.8 (100)
Be-cAAC^{Dip}	1.644 (1.645)	177.8 (174.3)	1.19 (1.26)	0.54 (0.51)	0.08 (0.12)	0.39 (0.73)	-0.35 (0.20)	+2 (+2)	-1 (-1)	78.9 (100)
Be-NacNac^{Me}	2.541	180.0	-0.90	0.61	0.07	0.06	-0.02	-2	+1	86.7
Be-BDI*	2.489	177.9	-0.42	0.51	-	-	-	-2	+1	73.0

[a] Charges, bond orders and EOS obtained at the CASSCF/cc-pVDZ//B3LYP-D3(BJ)/def2-SVP level (*BDI systems evaluated with B3LYP-D3(BJ)/def2-SVP) using the TFVC atomic definition. [b] Two pseudo-degenerated EFOs (in occupancy), one from each ligand.

Table S5. Dissociation energy (D_0) values (in kcal/mol) of the studied compounds in two plausible fragmentation patters obtained at the B3LYP-D3(BJ)/def2-TZVPP//B3LYP-D3(BJ)/def2-SVP level.[a] Gas-phase, [b] using THF as solvent.

E-L2 system	D_0 (E(0) + 2L(0))[a]	D_0 (E(+2) + 2L(-1))[a]	D_0 (E(0) + 2L(0))[b]	D_0 (E(+2) + 2L(-1))[b]
Mg-NHC^{Me}	-18.2	-643.8	-11.0	-198.6
Mg-NHC^{Dip}	-25.2	-578.7	-22.5	-175.2
Mg-cAAC^{Me}	-29.7	-616.1	-21.7	-179.6
Mg-cAAC^{Dip}	-42.7	-604.1	-37.0	-190.9
Mg-NacNac^{Me}	-49.0	-572.3	-45.5	-166.1
Mg-BDI*	-63.9	-572.7	-59.5	-174.2
Be-NHC^{Me}	-66.4	-796.3	-61.5	-317.4
Be-NHC^{Dip}	-75.0	-732.9	-69.5	-290.5
Be-cAAC^{Me}	-99.1	-789.9	-91.6	-317.8
Be-cAAC^{Dip}	-107.4	-773.3	-100.3	-322.4
Be-NacNac^{Me}	-70.0	-697.7	-64.8	-253.6
Be-BDI*	-83.9	-697.1	-79.0	-262.0

Table S6. Dissociation energy (D_0) values (in kcal/mol) of the studied compounds in two plausible fragmentation patters obtained at the M062X/def2-TZVPP//M062X/def2-SVP level.[a] Gas-phase, [b] using THF as solvent.

E-L2 system	D_0 (E(0) + 2L(0))[a]	D_0 (E(+2) + 2L(-1))[a]	D_0 (E(0) + 2L(0))[b]	D_0 (E(+2) + 2L(-1))[b]
Mg-NHC^{Me}	-15.5	-629.6	-8.6	-189.7
Mg-NHC^{Dip}	-18.6	-577.8	-16.3	-163.8
Mg-cAAC^{Me}	-28.3	-608.2	-24.9	-174.0
Mg-cAAC^{Dip}	-39.0	-598.8	-33.8	-182.5
Mg-NacNac^{Me}	-46.1	-566.4	-43.7	-158.6
Mg-BDI*	-48.7	-560.0	-44.6	-159.0
Be-NHC^{Me}	-62.6	-780.3	-55.9	-304.7
Be-NHC^{Dip}	-69.9	-732.7	-64.0	-279.1
Be-cAAC^{Me}	-94.3	-777.8	-86.4	-303.2
Be-cAAC^{Dip}	-102.5	-765.9	-95.2	-311.6
Be-NacNac^{Me}	-66.9	-690.9	-62.6	-245.1
Be-BDI*	-68.3	-683.3	-63.1	-245.2

Table S7. Dissociation energy (D_0) values (in kcal/mol) of the studied compounds in two plausible fragmentation patters obtained at the wB97xD/def2-TZVPP//wB97xD/def2-SVP level.[a] Gas-phase, [b] using THF as solvent.

E-L2 system	D_0 (E(0) + 2L(0))[a]	D_0 (E(+2) + 2L(-1))[a]	D_0 (E(0) + 2L(0))[b]	D_0 (E(+2) + 2L(-1))[b]
Mg-NHC^{Me}	-15.9	-613.2	-9.7	-174.4
Mg-NHC^{Dip}	-22.6	-587.5	-21.2	-185.2
Mg-cAAC^{Me}	-29.6	-598.1	-27.4	-165.2
Mg-cAAC^{Dip}	-44.9	-592.7	-38.2	-175.2
Mg-NacNac^{Me}	-55.1	-556.1	-51.3	-148.7
Mg-BDI*	-71.6	-561.8	-67.4	-159.4
Be-NHC^{Me}	-55.3	-760.2	-51.0	-287.0
Be-NHC^{Dip}	-65.7	-738.2	-59.9	-295.2
Be-cAAC^{Me}	-92.8	-768.9	-84.8	-293.9
Be-cAAC^{Dip}	-103.9	-759.2	-96.1	-304.4
Be-NacNac^{Me}	-72.8	-681.3	-67.2	-236.0
Be-BDI*	-89.0	-686.8	-84.7	-248.0

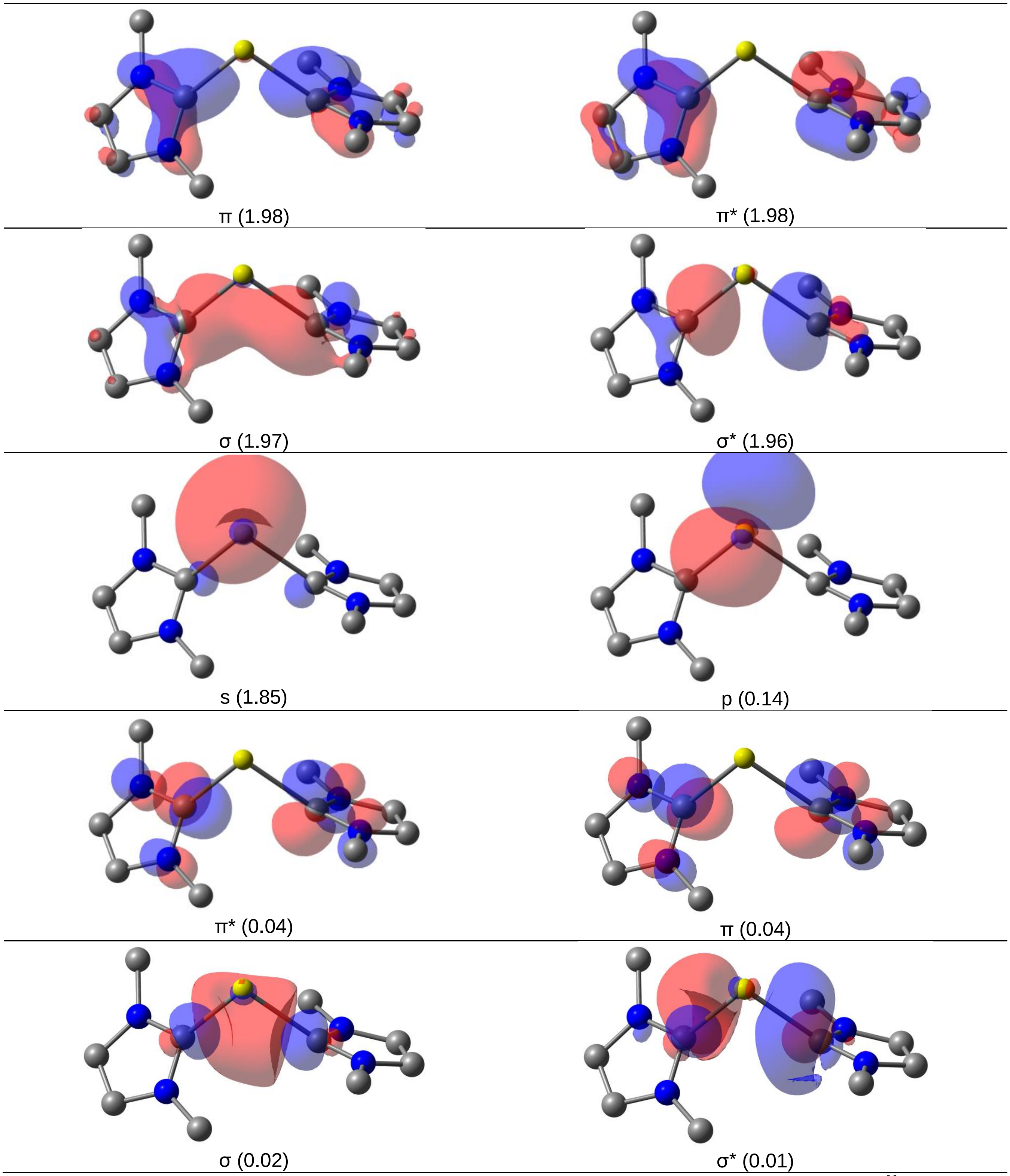

Figure S1. Active-space natural orbitals with its occupancies (in e) for the singlet spin-state **Mg-NHC^{Me}** obtained at the CASSCF(10,10)/cc-pVDZ//B3LYP-D3(BJ)/def2-SVP level of theory. Isocontour value of 0.05 a.u. Hydrogen atoms were omitted for clarity.

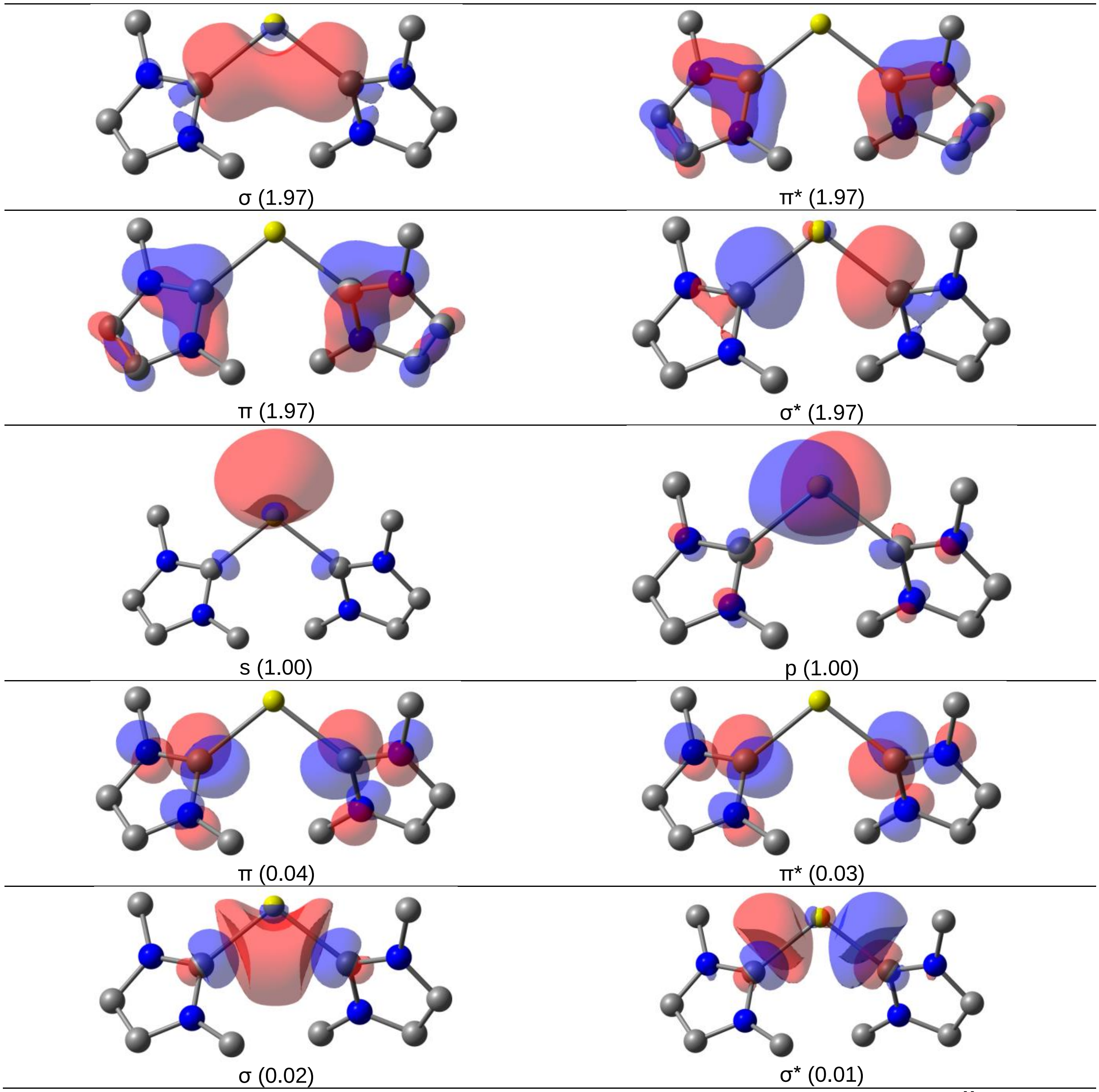

Figure S2. Active-space natural orbitals with its occupancies (in e) for the triplet spin-state **Mg-NHC$^{\mathbf{Me}}$** obtained at the CASSCF(10,10)/cc-pVDZ//B3LYP-D3(BJ)/def2-SVP level of theory. Isocontour value of 0.05 a.u. Hydrogen atoms were omitted for clarity.

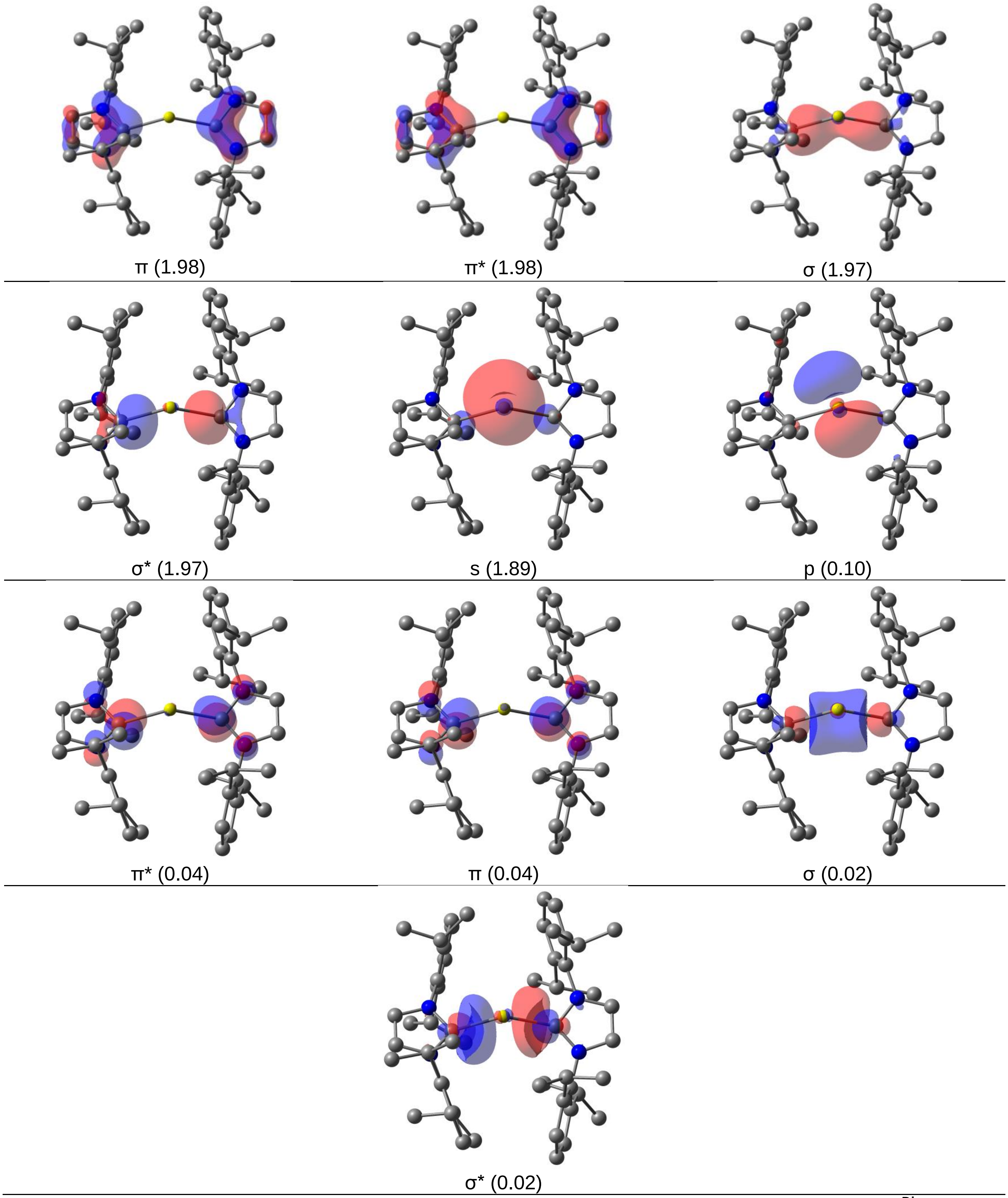

Figure S3. Active-space natural orbitals with its occupancies (in e) for the singlet spin-state **Mg-NHC^{Dip}** obtained at the CASSCF(10,10)/cc-pVDZ//B3LYP-D3(BJ)/def2-SVP level of theory. Isocontour value of 0.05 a.u. Hydrogen atoms were omitted for clarity.

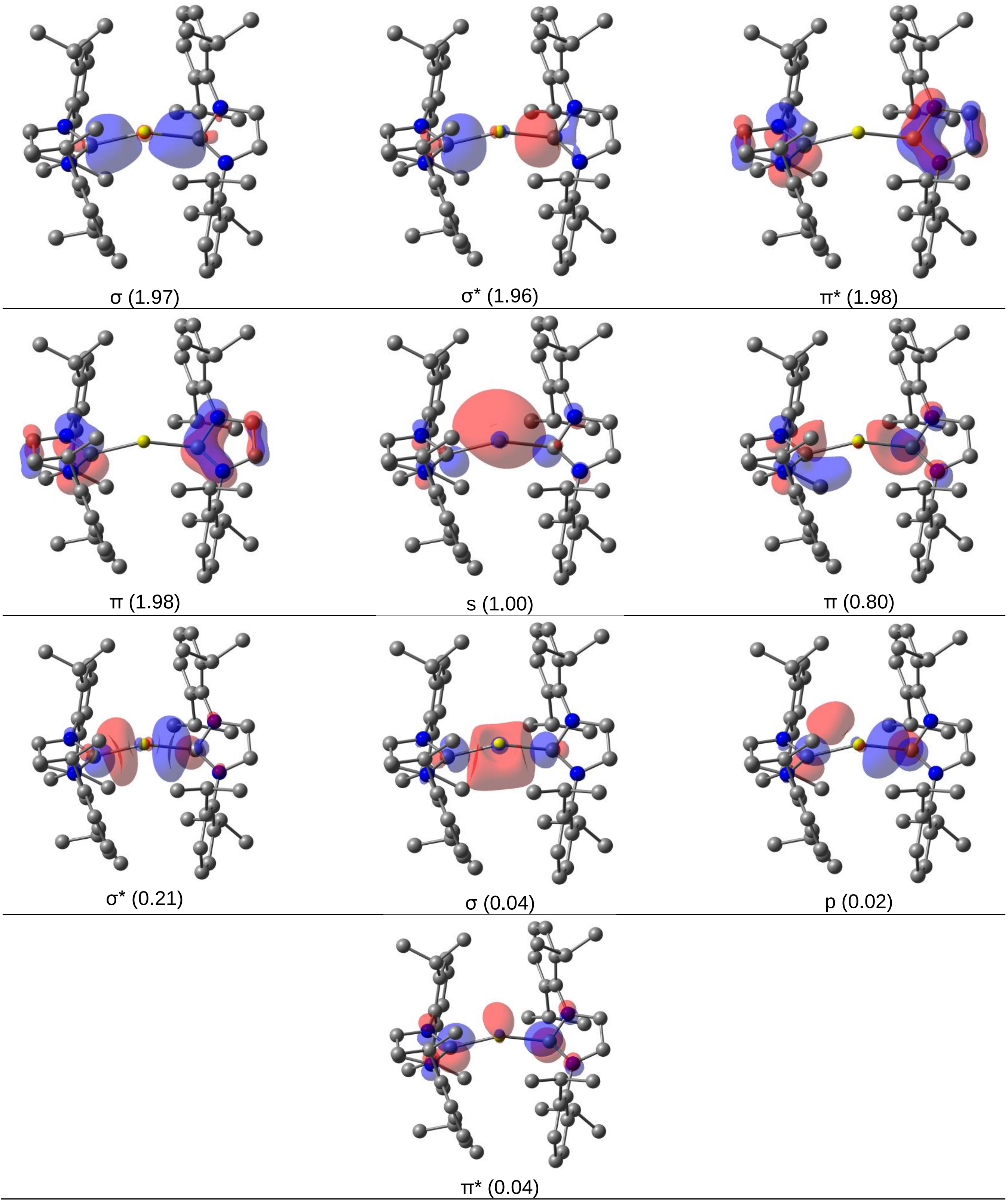

Figure S4. Active-space natural orbitals with its occupancies (in e) for the triplet spin-state **Mg-NHC^{Dip}** obtained at the CASSCF(10,10)/cc-pVDZ//B3LYP-D3(BJ)/def2-SVP level of theory. Isocontour value of 0.05 a.u. Hydrogen atoms were omitted for clarity.

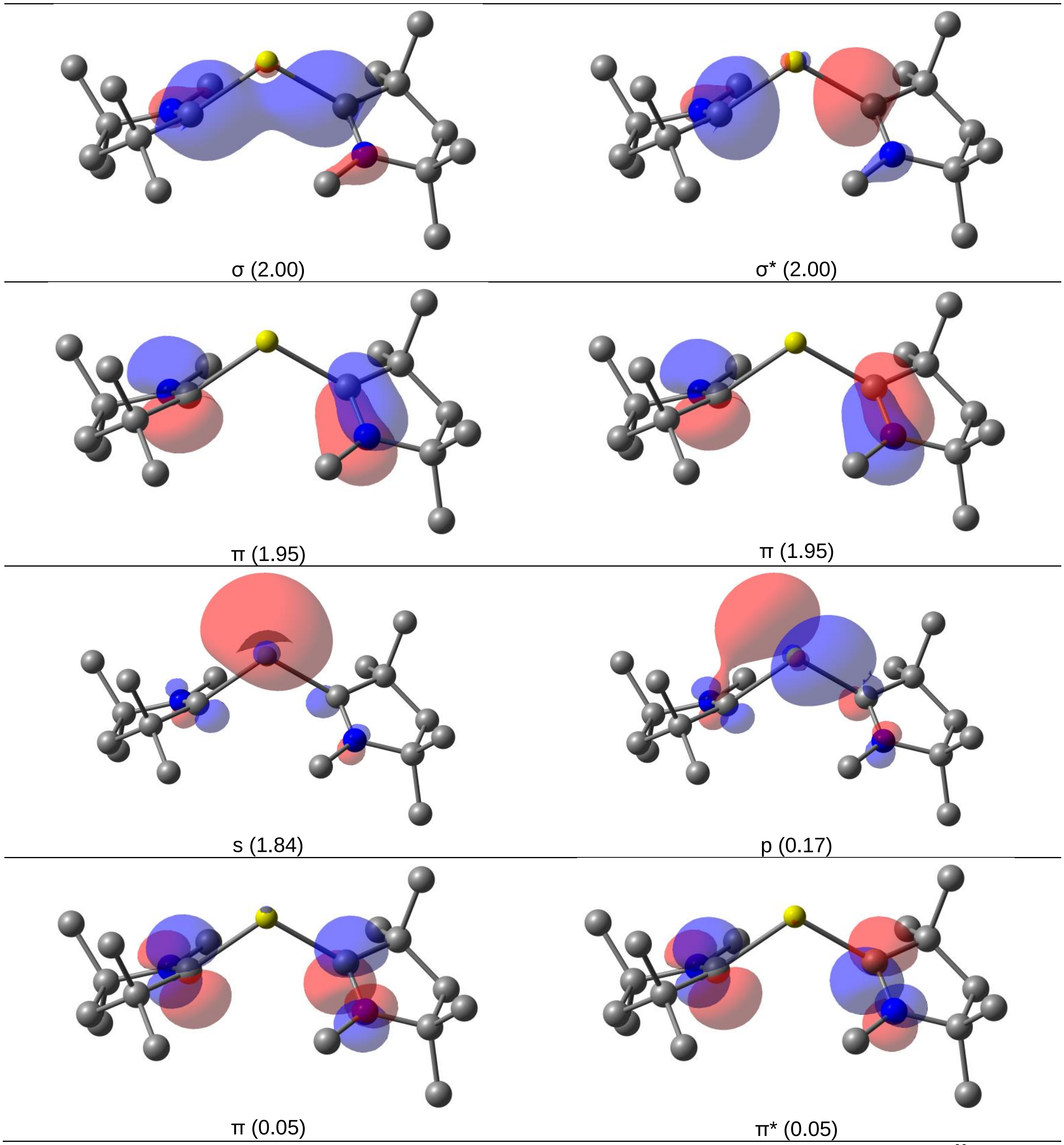

Figure S5. Active-space natural orbitals with its occupancies (in e) for the singlet spin-state **Mg-cAAC^{Me}** obtained at the CASSCF(10,8)/cc-pVDZ//B3LYP-D3(BJ)/def2-SVP level of theory. Isocontour value of 0.05 a.u. Hydrogen atoms were omitted for clarity.

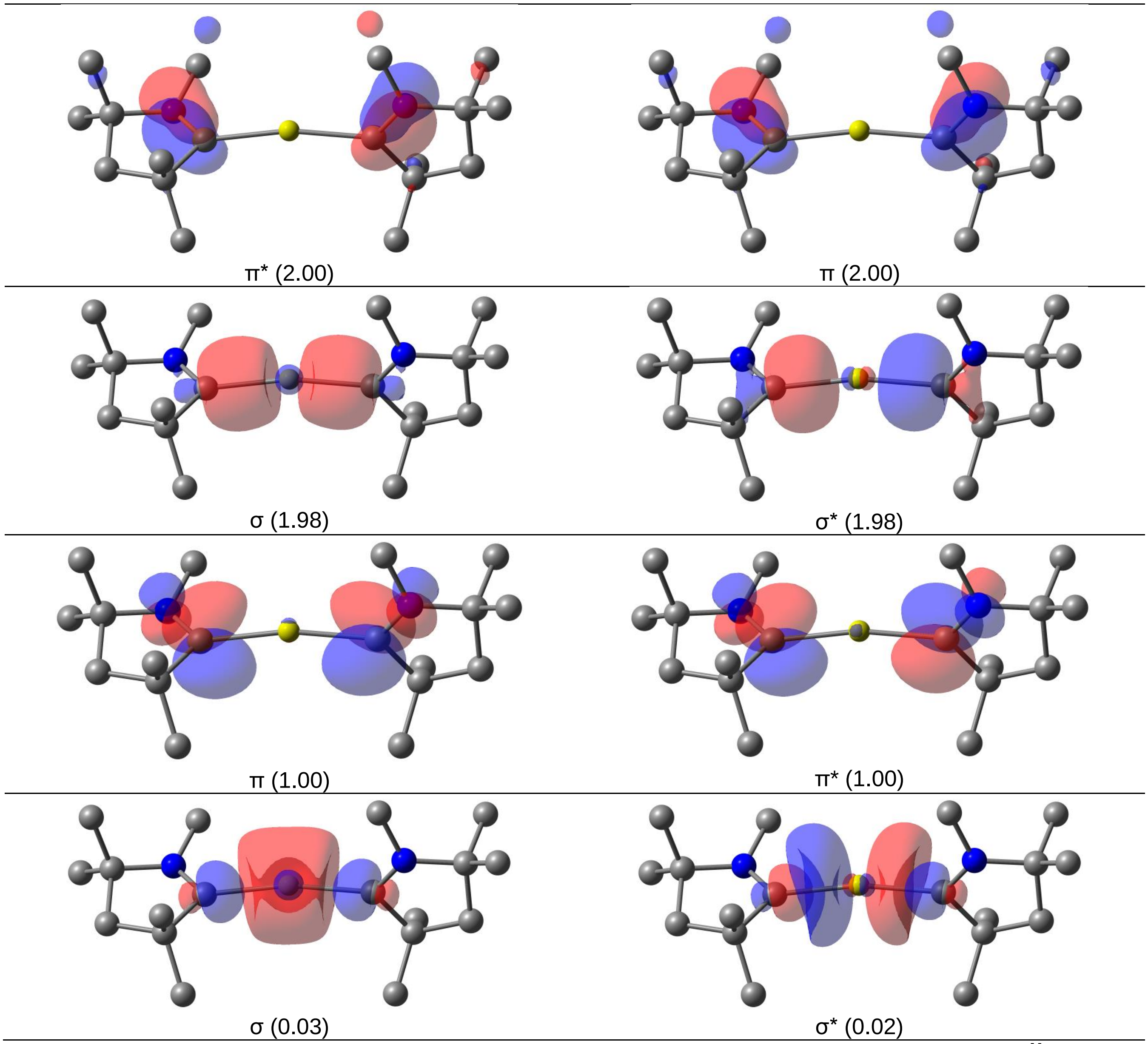

Figure S6. Active-space natural orbitals with its occupancies (in e) for the triplet spin-state **Mg-cAAC^{Me}** obtained at the CASSCF(10,8)/cc-pVDZ//B3LYP-D3(BJ)/def2-SVP level of theory. Isocontour value of 0.05 a.u. Hydrogen atoms were omitted for clarity.

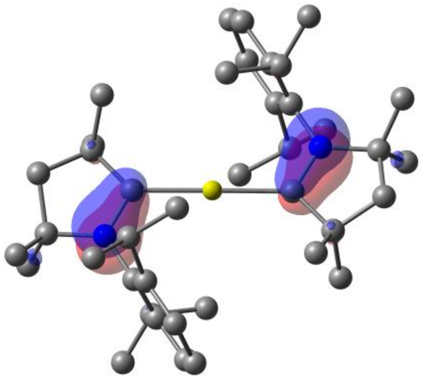

π (2.00)

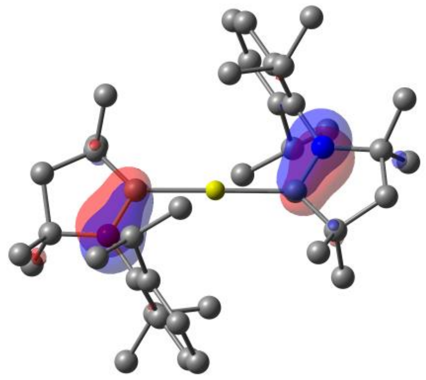

π* (2.00)

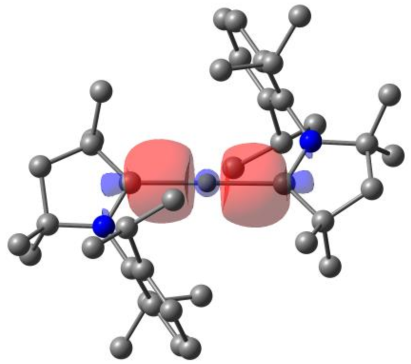

σ (1.98)

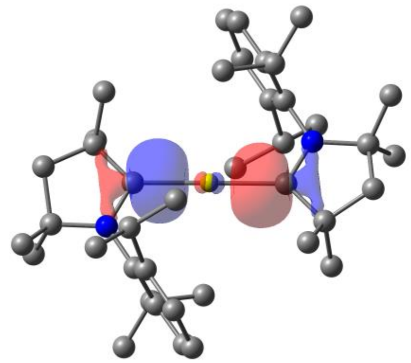

σ* (1.98)

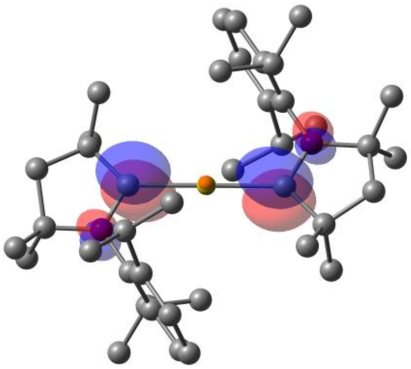

π (1.21)

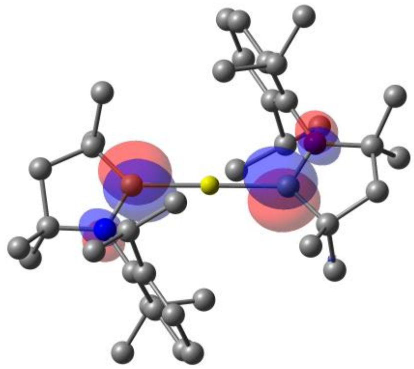

π* (0.79)

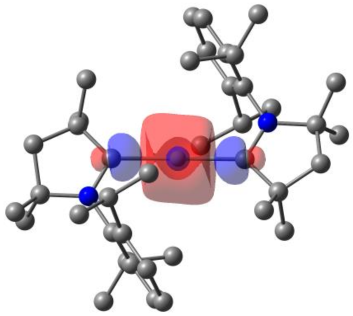

σ (0.03)

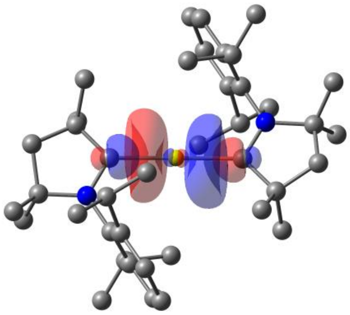

σ* (0.01)

Figure S7. Active-space natural orbitals with its occupancies (in e) for the singlet spin-state **Mg-cAAC^{Dip}** obtained at the CASSCF(10,8)/cc-pVDZ//B3LYP-D3(BJ)/def2-SVP level of theory. Isocontour value of 0.05 a.u. Hydrogen atoms were omitted for clarity.

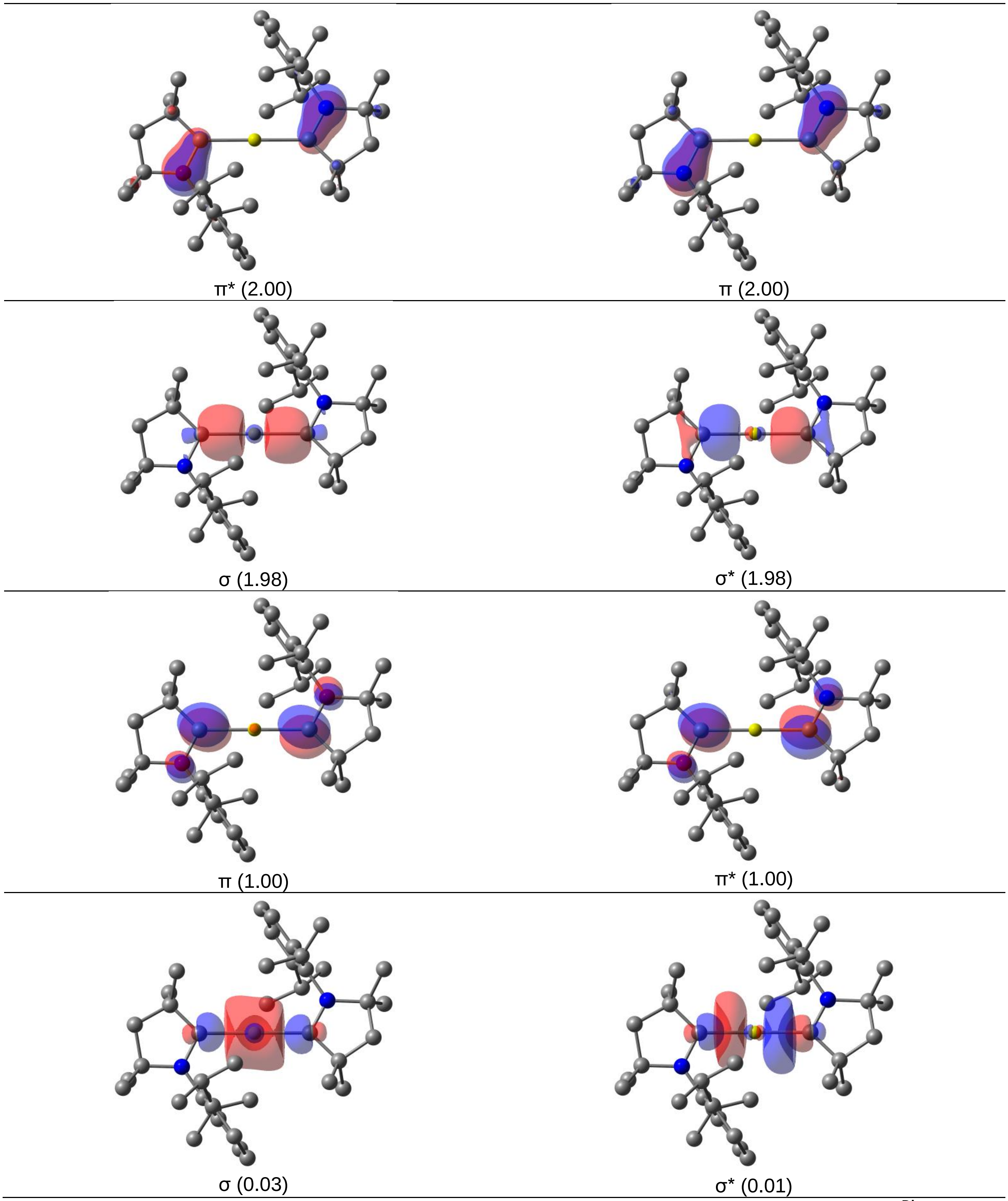

Figure S8. Active-space natural orbitals with its occupancies (in e) for the triplet spin-state **Mg-cAAC**$^{\mathbf{Dip}}$ obtained at the CASSCF(10,8)/cc-pVDZ//B3LYP-D3(BJ)/def2-SVP level of theory. Isocontour value of 0.05 a.u. Hydrogen atoms were omitted for clarity.

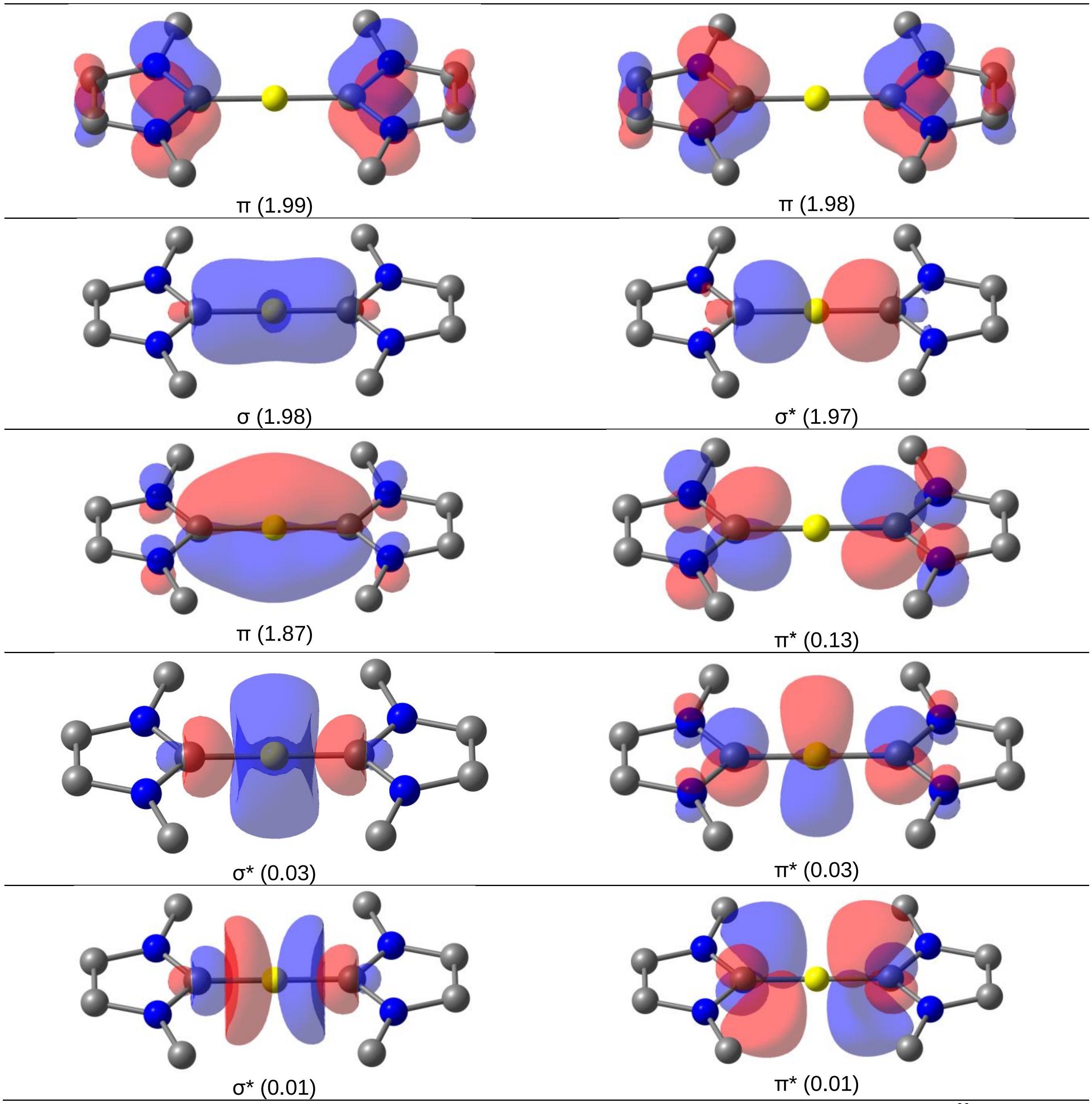

Figure S9. Active-space natural orbitals with its occupancies (in e) for the singlet spin-state **Be-NHC^{Me}** obtained at the CASSCF(10,10)/cc-pVDZ//B3LYP-D3(BJ)/def2-SVP level of theory. Isocontour value of 0.05 a.u. Hydrogen atoms were omitted for clarity.

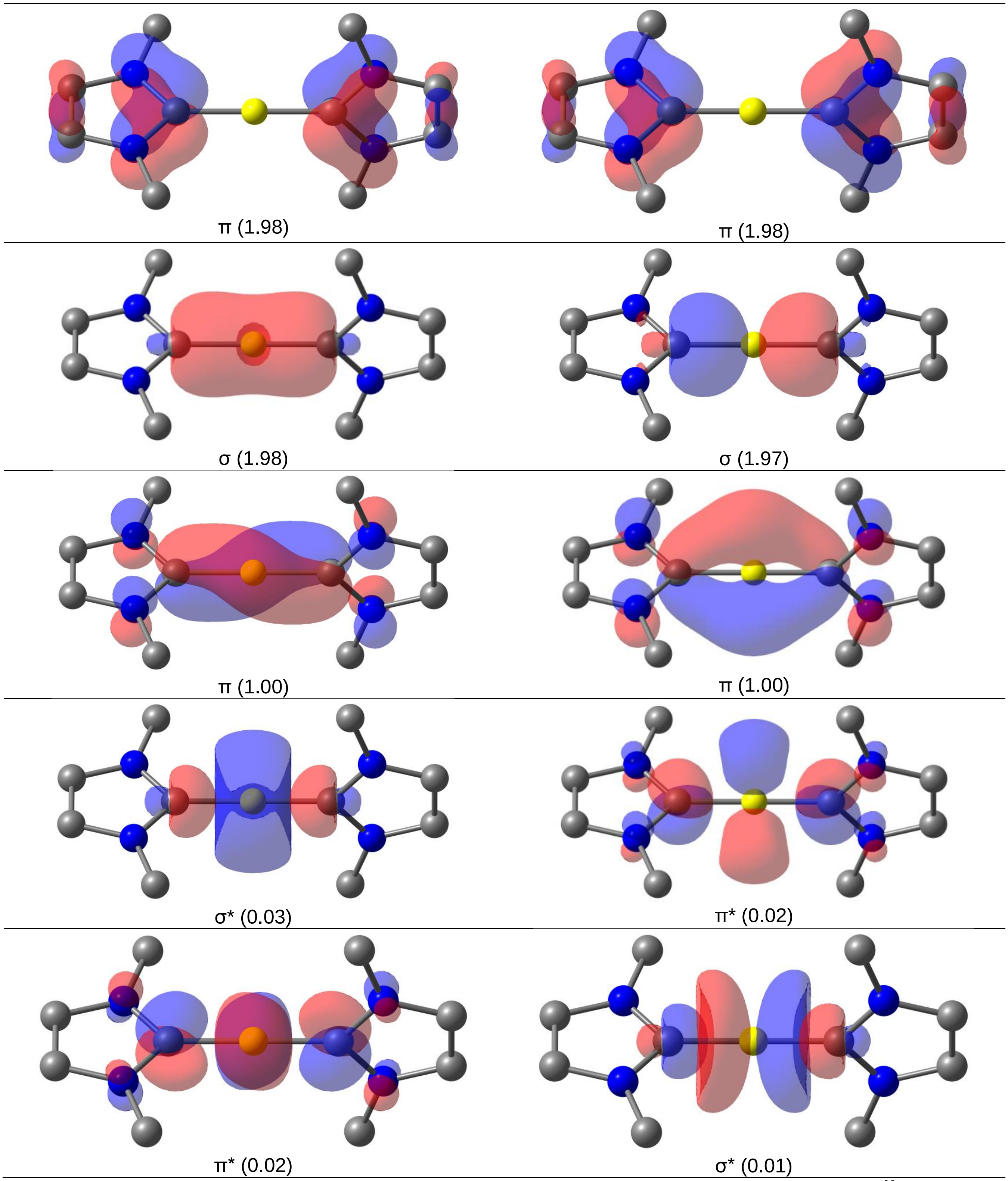

Figure S10. Active-space natural orbitals with its occupancies (in e) for the triplet spin-state **Be-NHC^{Me}** obtained at the CASSCF(10,10)/cc-pVDZ//B3LYP-D3(BJ)/def2-SVP level of theory. Isocontour value of 0.05 a.u. Hydrogen atoms were omitted for clarity.

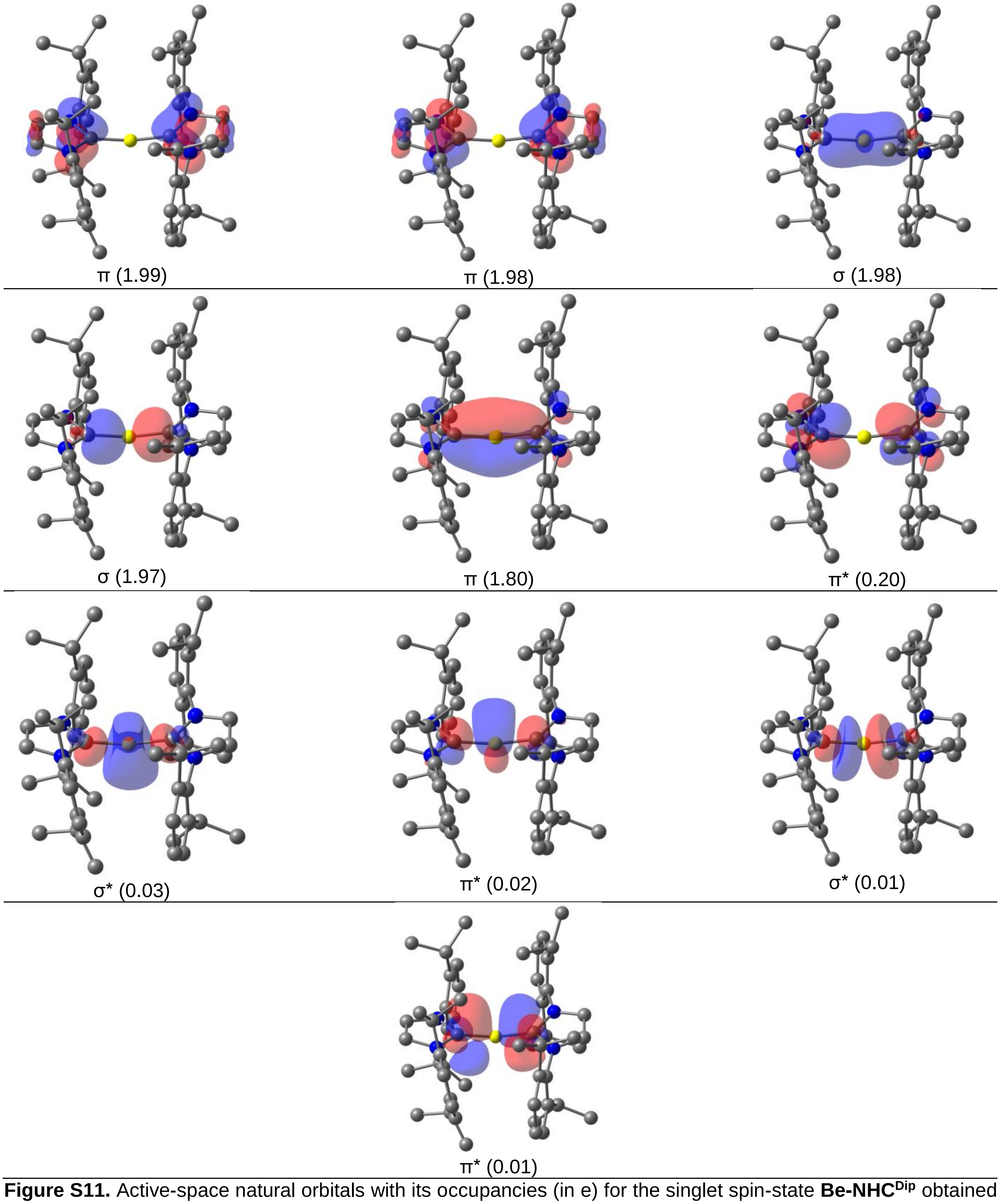

Figure S11. Active-space natural orbitals with its occupancies (in e) for the singlet spin-state **Be-NHC$^{\mathbf{Dip}}$** obtained at the CASSCF(10,10)/cc-pVDZ//B3LYP-D3(BJ)/def2-SVP level of theory. Isocontour value of 0.05 a.u. Hydrogen atoms were omitted for clarity.

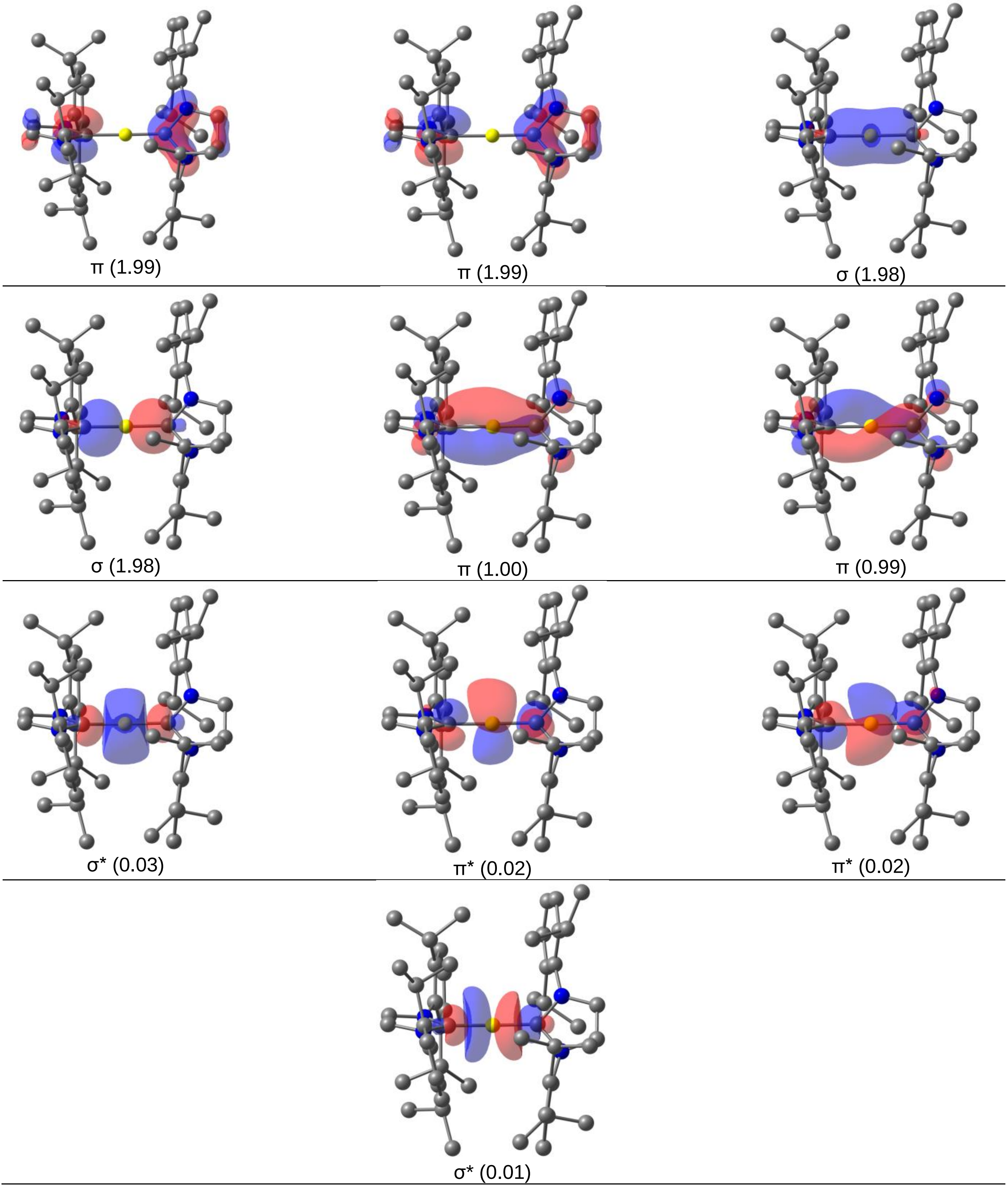

Figure S12. Active-space natural orbitals with its occupancies (in e) for the triplet spin-state **Be-NHC^{Dip}** obtained at the CASSCF(10,10)/cc-pVDZ//B3LYP-D3(BJ)/def2-SVP level of theory. Isocontour value of 0.05 a.u. Hydrogen atoms were omitted for clarity.

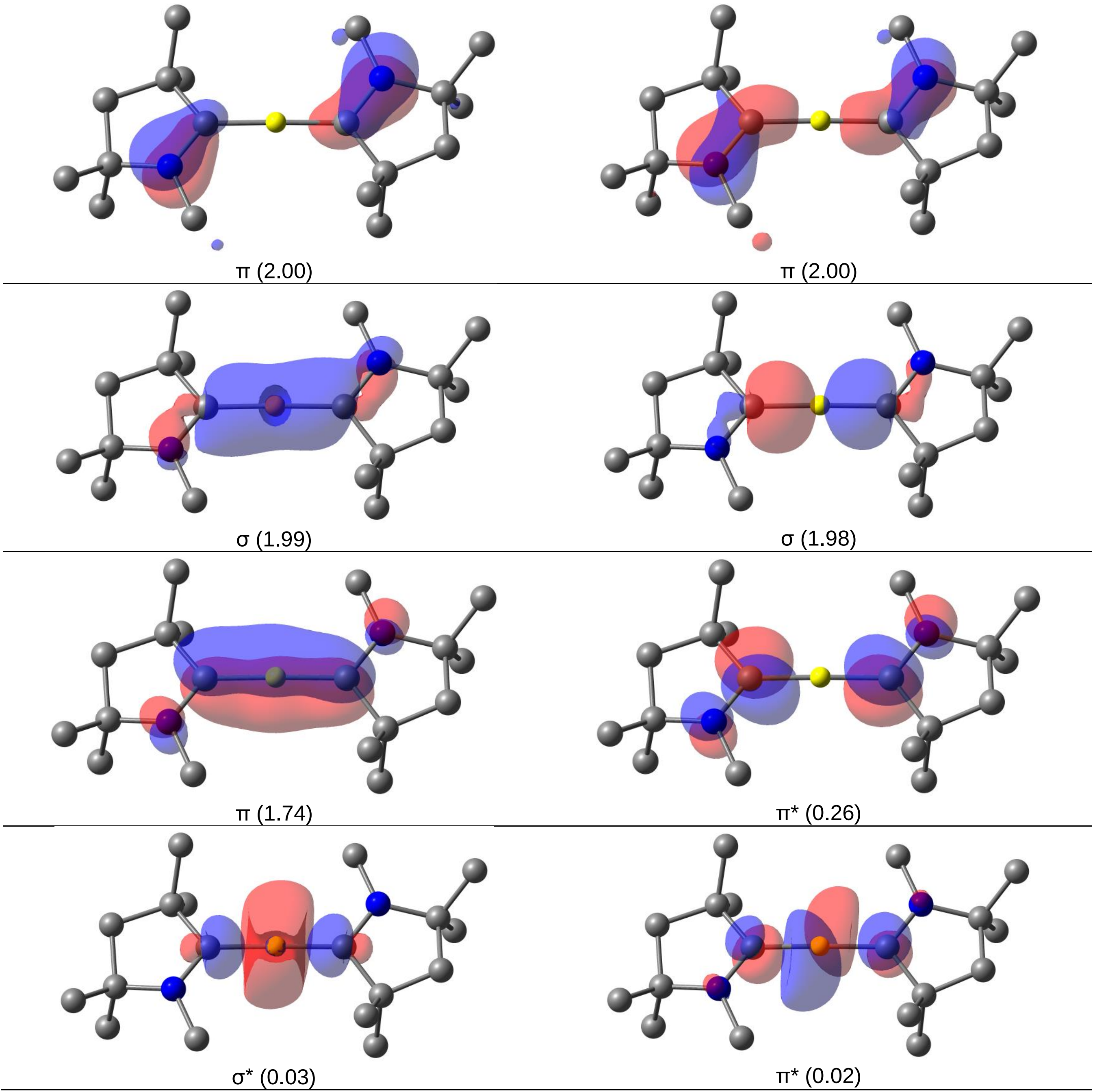

Figure S13. Active-space natural orbitals with its occupancies (in e) for the singlet spin-state **Be-cAAC$^{\mathbf{Me}}$** obtained at the CASSCF(10,8)/cc-pVDZ//B3LYP-D3(BJ)/def2-SVP level of theory. Isocontour value of 0.05 a.u. Hydrogen atoms were omitted for clarity.

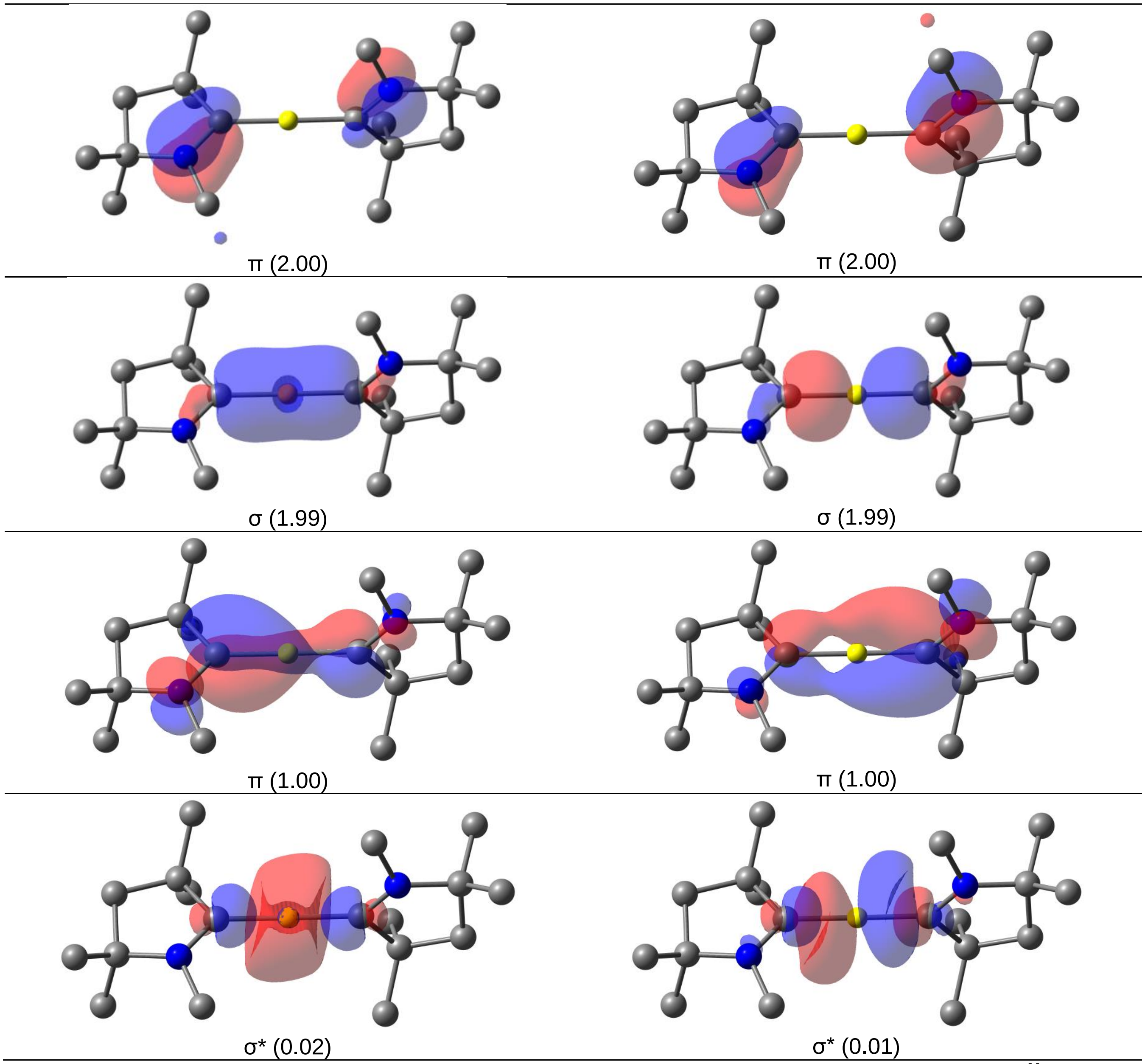

Figure S14. Active-space natural orbitals with its occupancies (in e) for the triplet spin-state **Be-cAAC$^{\mathbf{Me}}$** obtained at the CASSCF(10,8)/cc-pVDZ//B3LYP-D3(BJ)/def2-SVP level of theory. Isocontour value of 0.05 a.u. Hydrogen atoms were omitted for clarity.

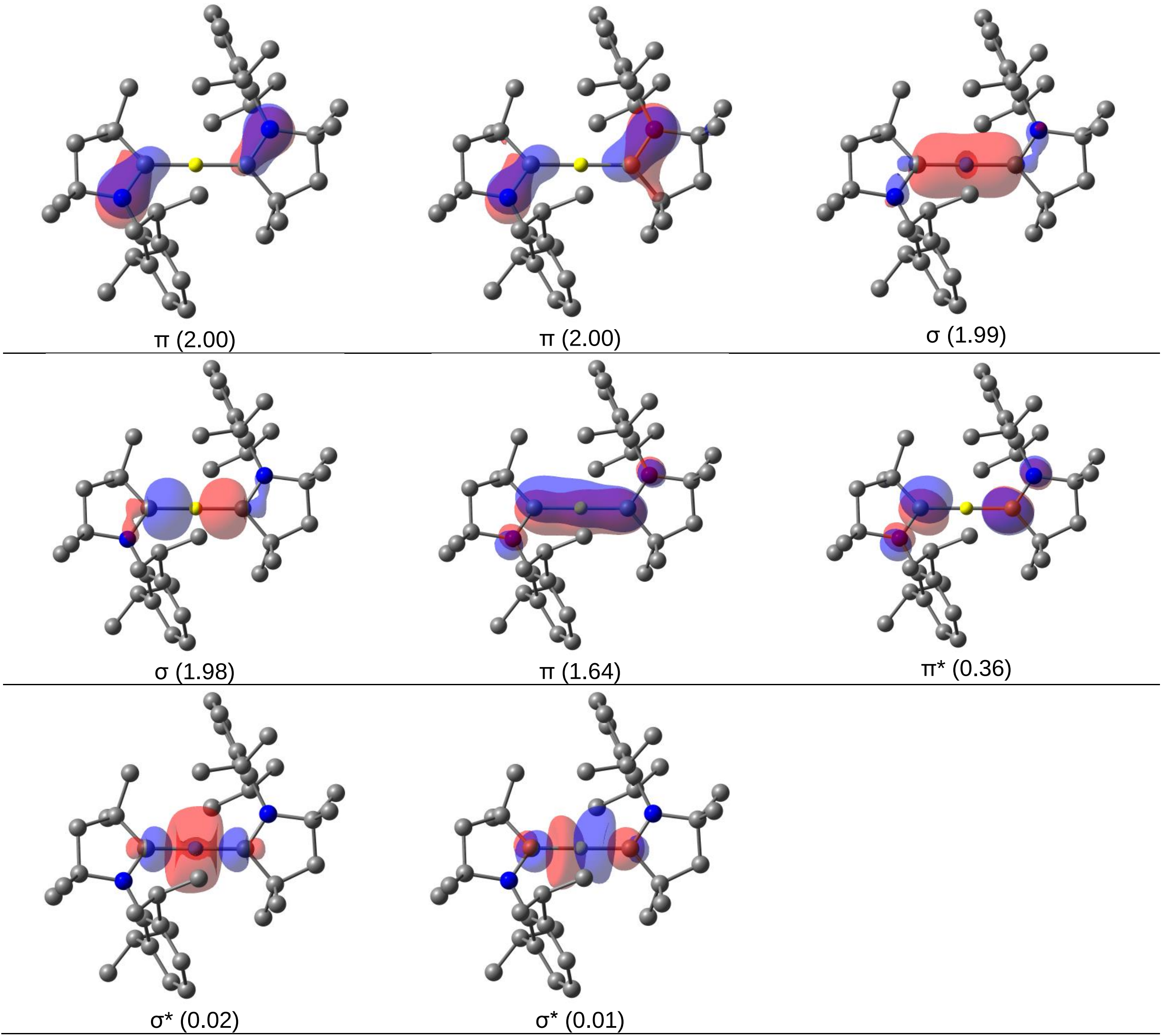

Figure S15. Active-space natural orbitals with its occupancies (in e) for the singlet spin-state **Be-cAAC$^{\mathbf{Dip}}$** obtained at the CASSCF(10,8)/cc-pVDZ//B3LYP-D3(BJ)/def2-SVP level of theory. Isocontour value of 0.05 a.u. Hydrogen atoms were omitted for clarity.

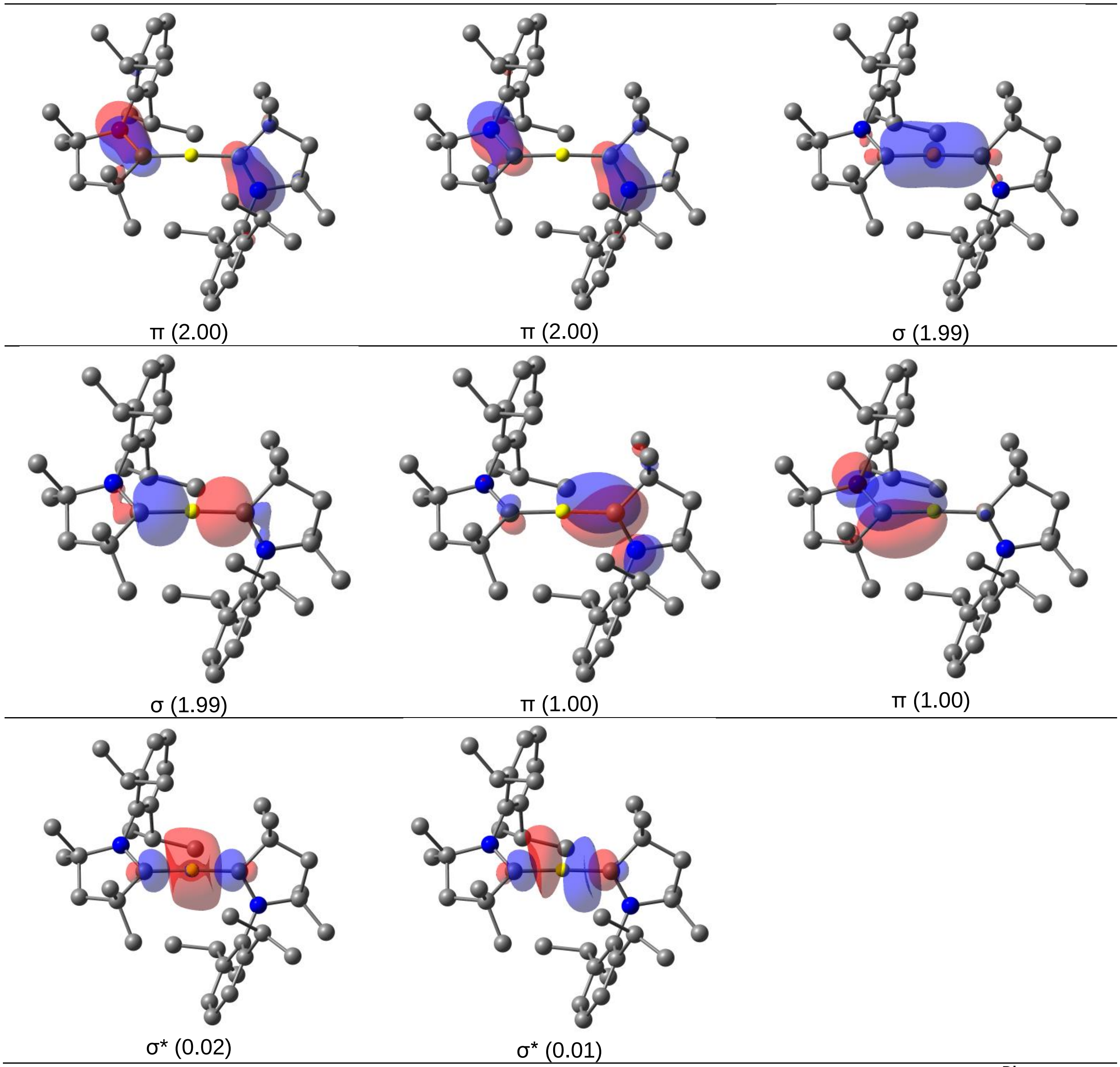

Figure S16. Active-space natural orbitals with its occupancies (in e) for the triplet spin-state **Be-cAAC$^{\mathbf{Dip}}$** obtained at the CASSCF(10,8)/cc-pVDZ//B3LYP-D3(BJ)/def2-SVP level of theory. Isocontour value of 0.05 a.u. Hydrogen atoms were omitted for clarity.

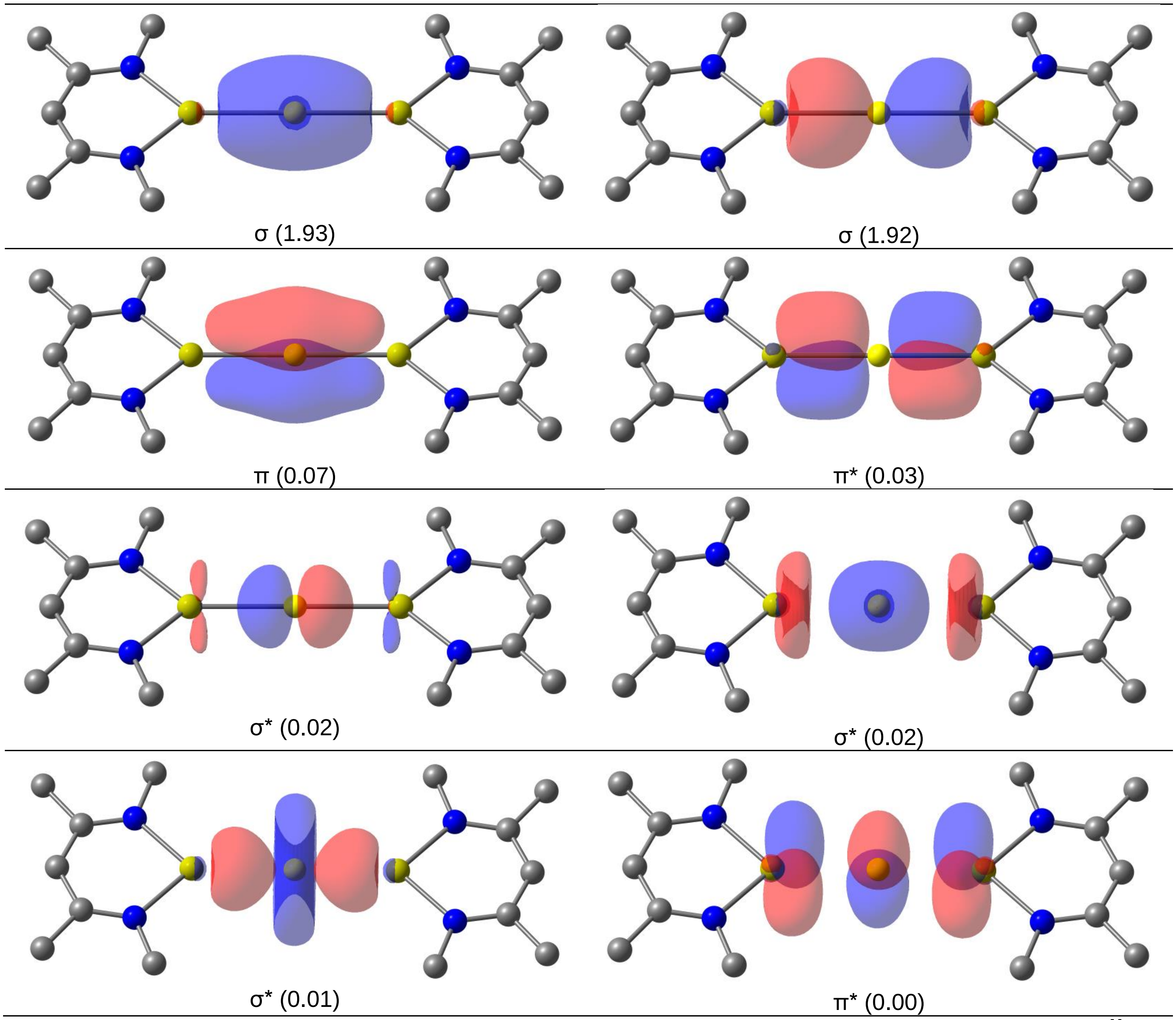

Figure S17. Active-space natural orbitals with its occupancies (in e) for the singlet spin-state **Be-NacNac^{Me}** obtained at the CASSCF(4,8)/cc-pVDZ//B3LYP-D3(BJ)/def2-SVP level of theory. Isocontour value of 0.05 a.u. Hydrogen atoms were omitted for clarity.

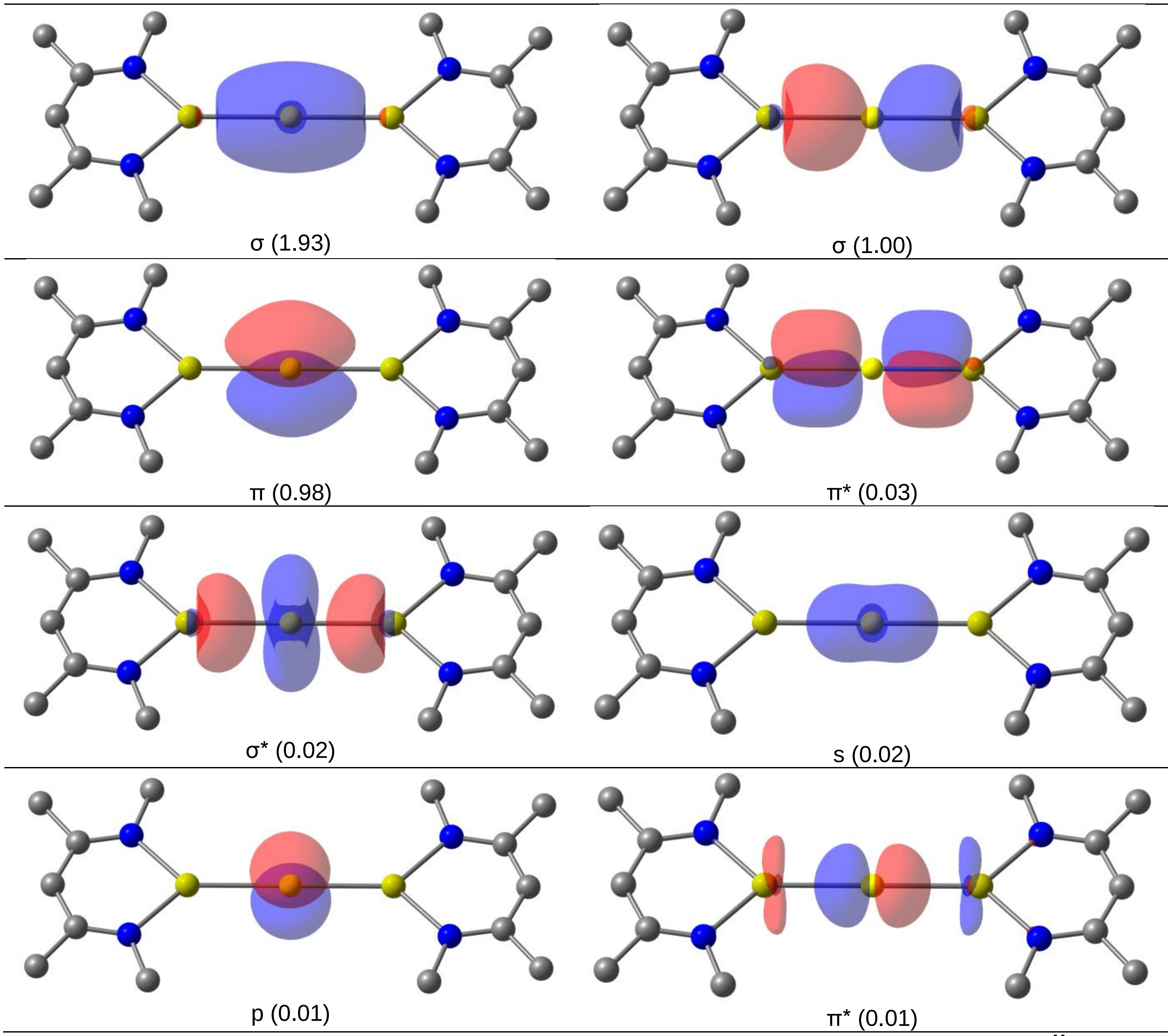

Figure S18. Active-space natural orbitals with its occupancies (in e) for the triplet spin-state **Be-NacNac$^{\mathbf{Me}}$** obtained at the CASSCF(4,8)/cc-pVDZ//B3LYP-D3(BJ)/def2-SVP level of theory. Isocontour value of 0.05 a.u. Hydrogen atoms were omitted for clarity.

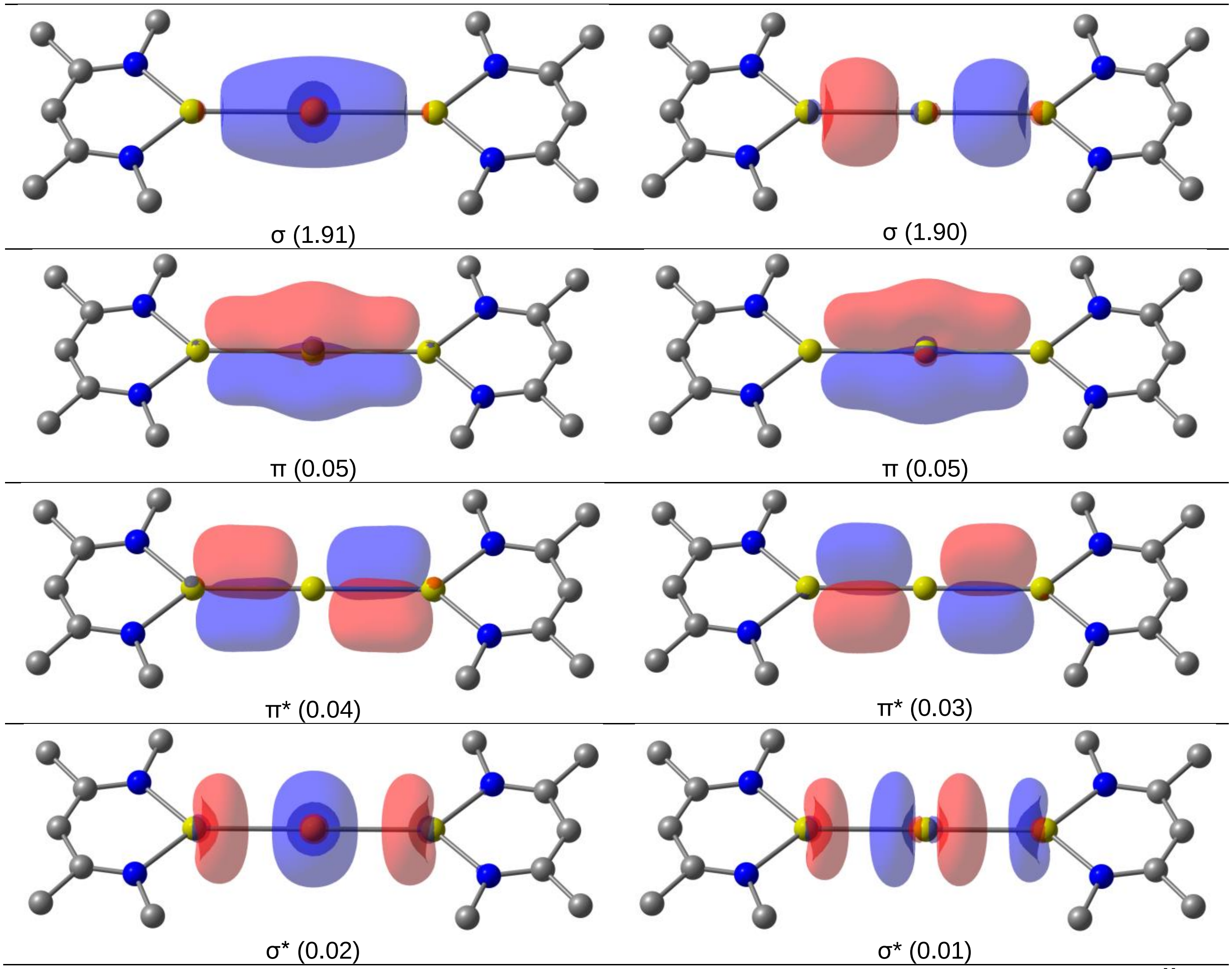

Figure S19. Active-space natural orbitals with its occupancies (in e) for the singlet spin-state **Mg-NacNac^{Me}** obtained at the CASSCF(4,8)/cc-pVDZ//B3LYP-D3(BJ)/def2-SVP level of theory. Isocontour value of 0.05 a.u. Hydrogen atoms were omitted for clarity.

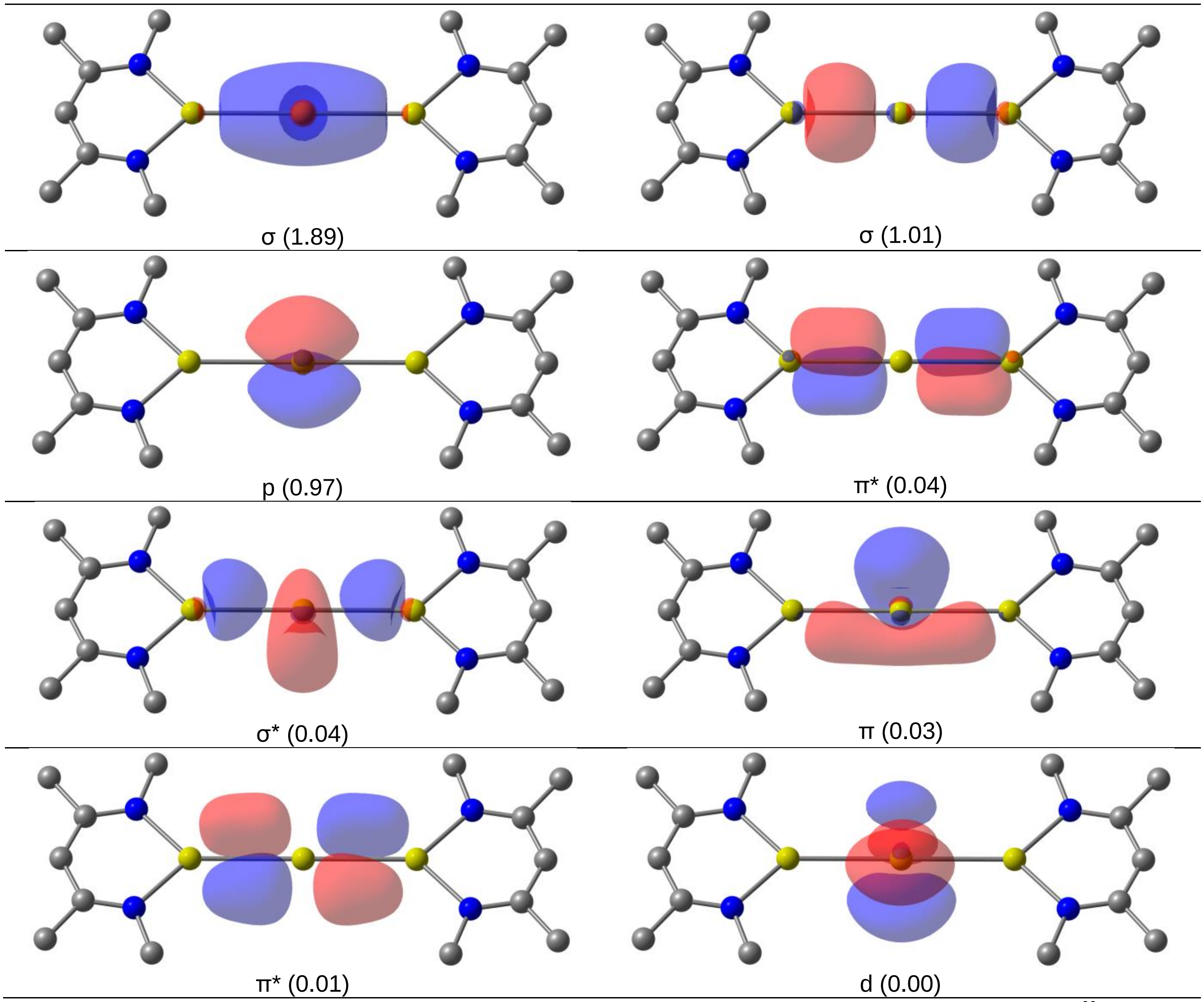

Figure S20. Active-space natural orbitals with its occupancies (in e) for the triplet spin-state **Mg-NacNac^{Me}** obtained at the CASSCF(4,8)/cc-pVDZ//B3LYP-D3(BJ)/def2-SVP level of theory. Isocontour value of 0.05 a.u. Hydrogen atoms were omitted for clarity.

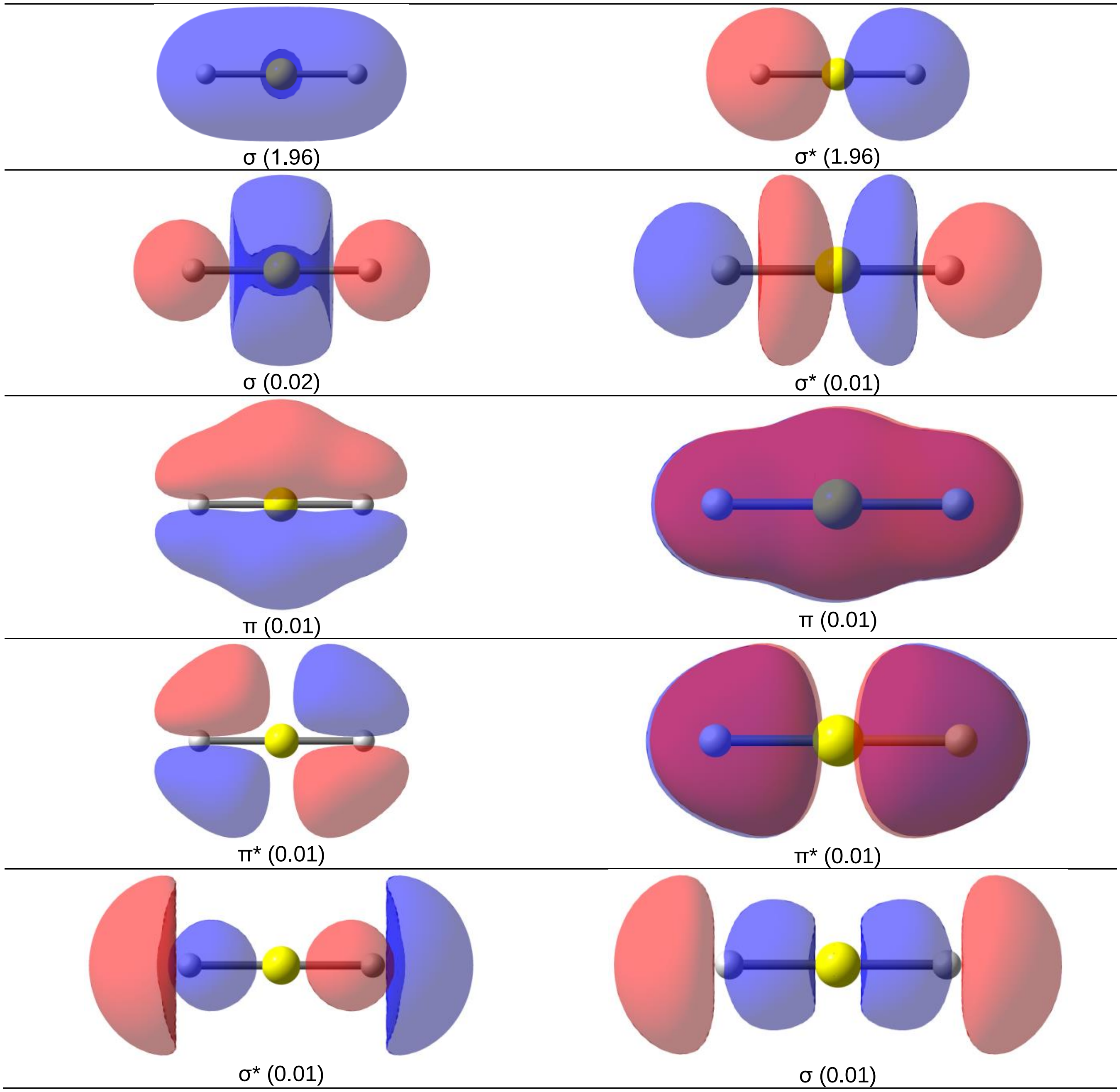

Figure S21. Active-space natural orbitals with its occupancies (in e) for the singlet spin-state **Be-H_2** obtained at the CASSCF(4,10)/cc-pVDZ//B3LYP-D3(BJ)/def2-SVP level of theory. Isocontour value of 0.05 a.u.

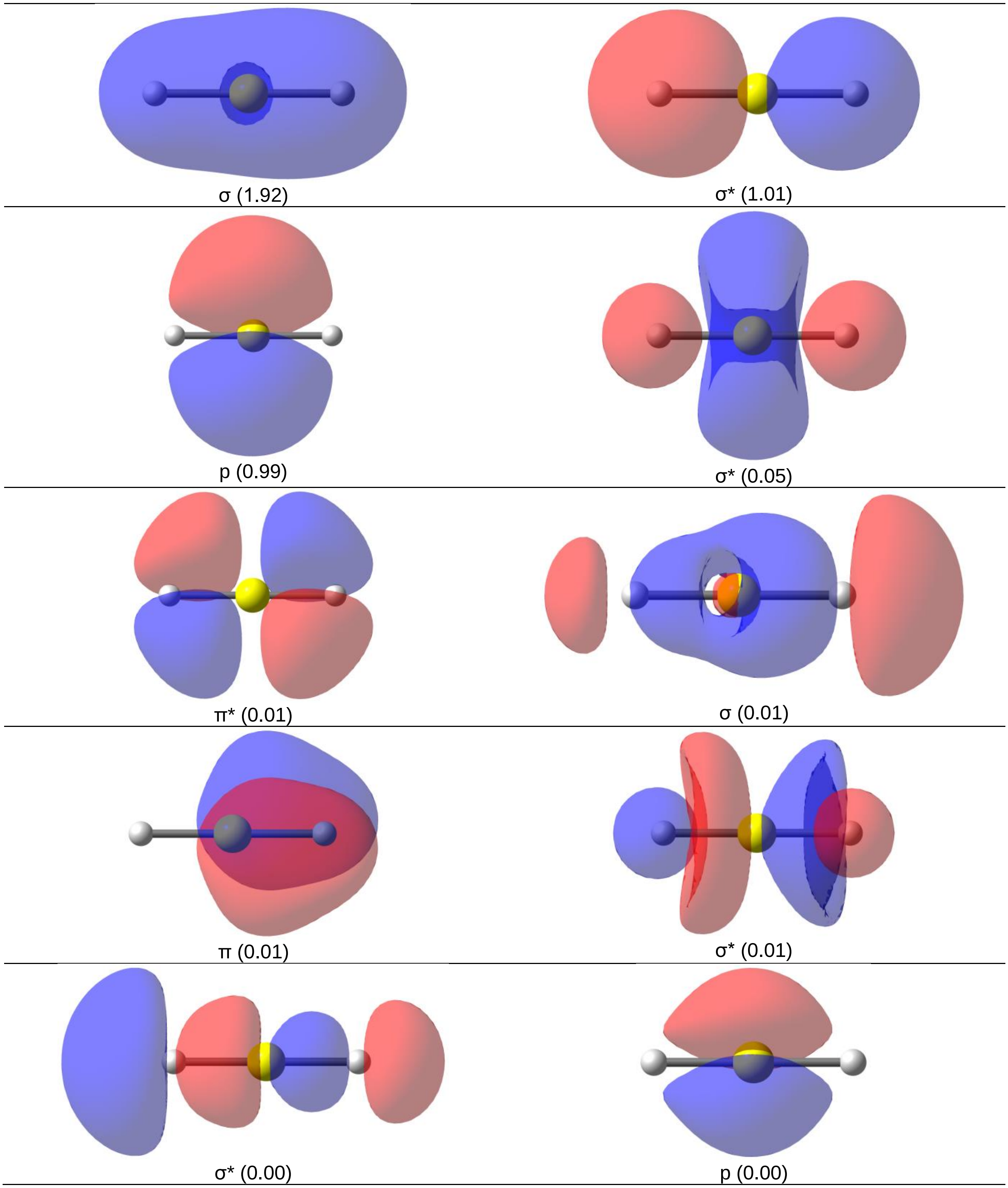

Figure S22. Active-space natural orbitals with its occupancies (in e) for the triplet spin-state **Be-H_2** obtained at the CASSCF(4,10)/cc-pVDZ//B3LYP-D3(BJ)/def2-SVP level of theory. Isocontour value of 0.05 a.u.

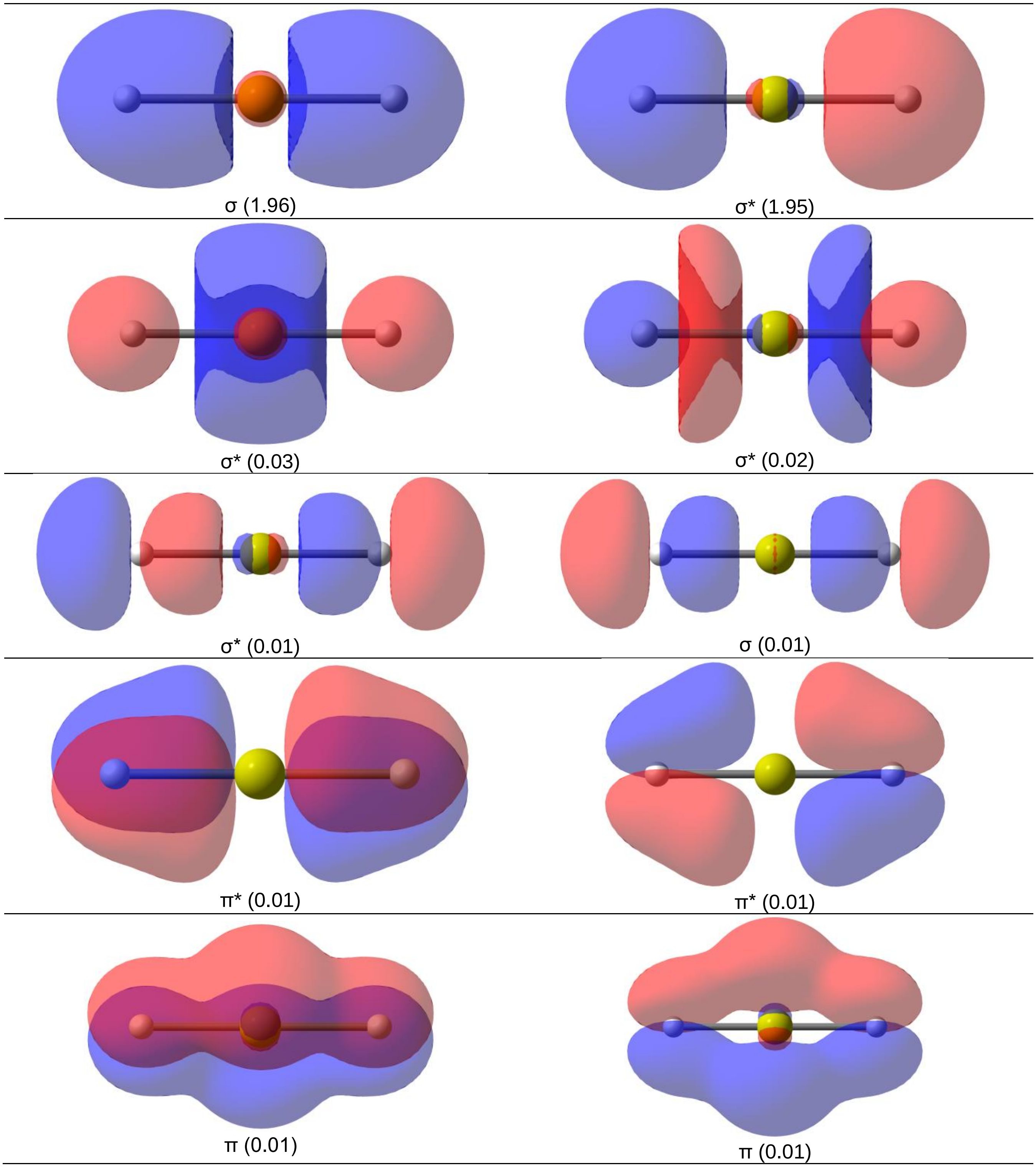

Figure S23. Active-space natural orbitals with its occupancies (in e) for the singlet spin-state **Mg-H$_2$** obtained at the CASSCF(4,10)/cc-pVDZ//B3LYP-D3(BJ)/def2-SVP level of theory. Isocontour value of 0.05 a.u.

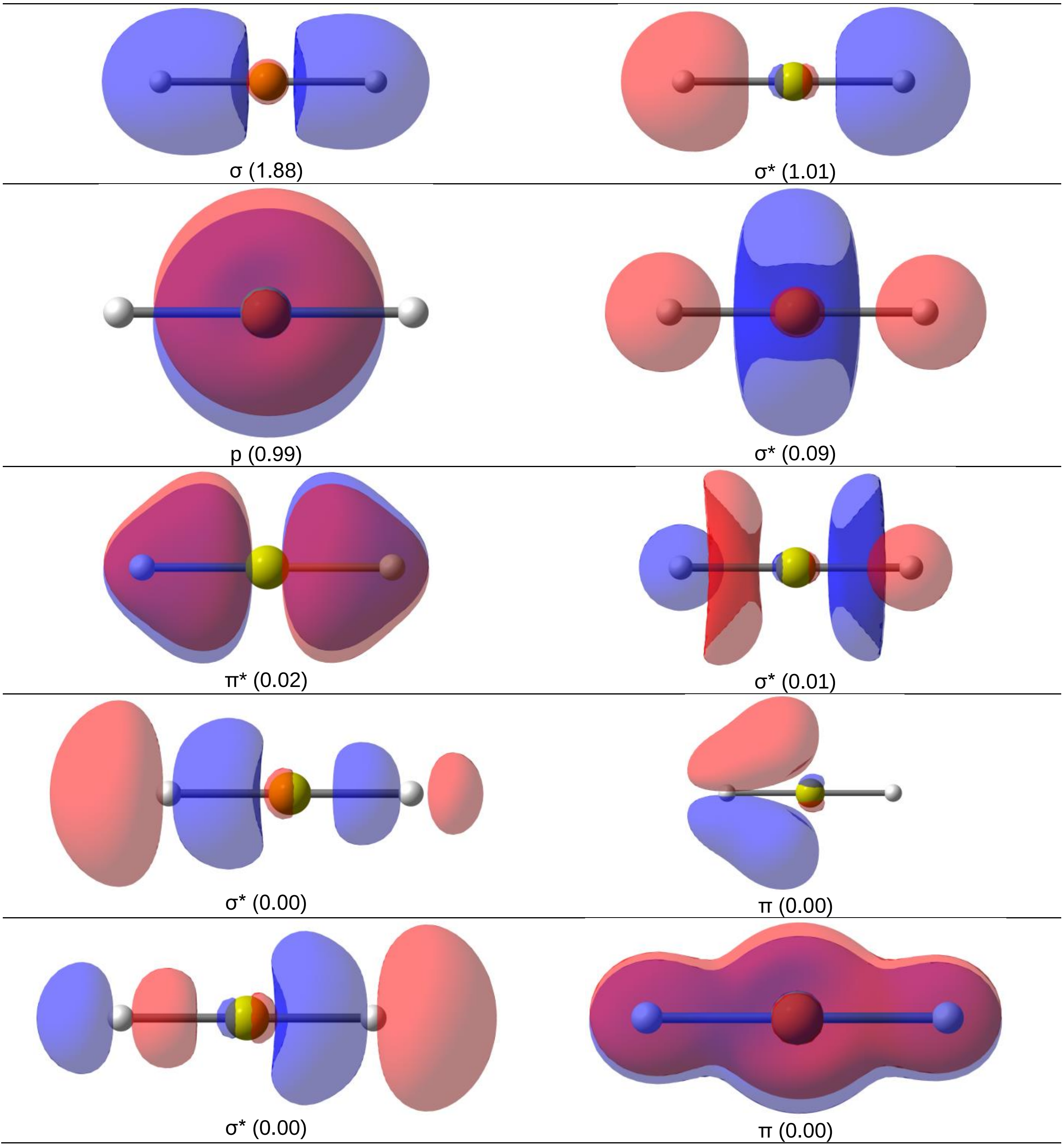

Figure S24. Active-space natural orbitals with its occupancies (in e) for the triplet spin-state **Mg-H_2** obtained at the CASSCF(4,10)/cc-pVDZ//B3LYP-D3(BJ)/def2-SVP level of theory. Isocontour value of 0.05 a.u.

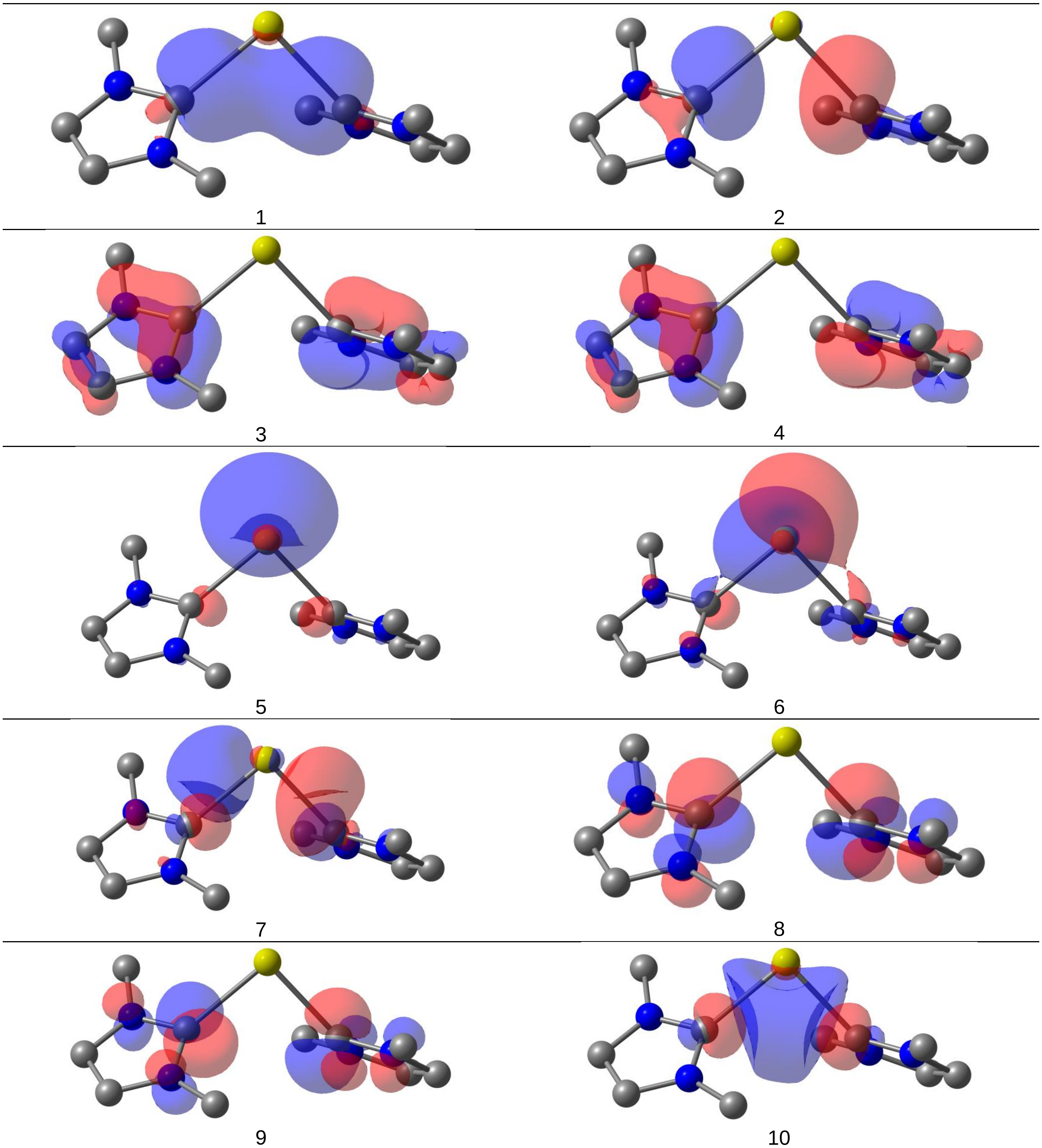

CI Coefficients: (2222200000) = -0.9354, (2222020000) = 0.2355

Figure S25. Active-space molecular orbitals and CI coefficients for the singlet spin-state **Mg-NHC^{Me}** obtained at the CASSCF(10,10)/cc-pVDZ//B3LYP-D3(BJ)/def2-SVP level of theory. Isocontour value of 0.05 a.u. Hydrogen atoms were omitted for clarity.

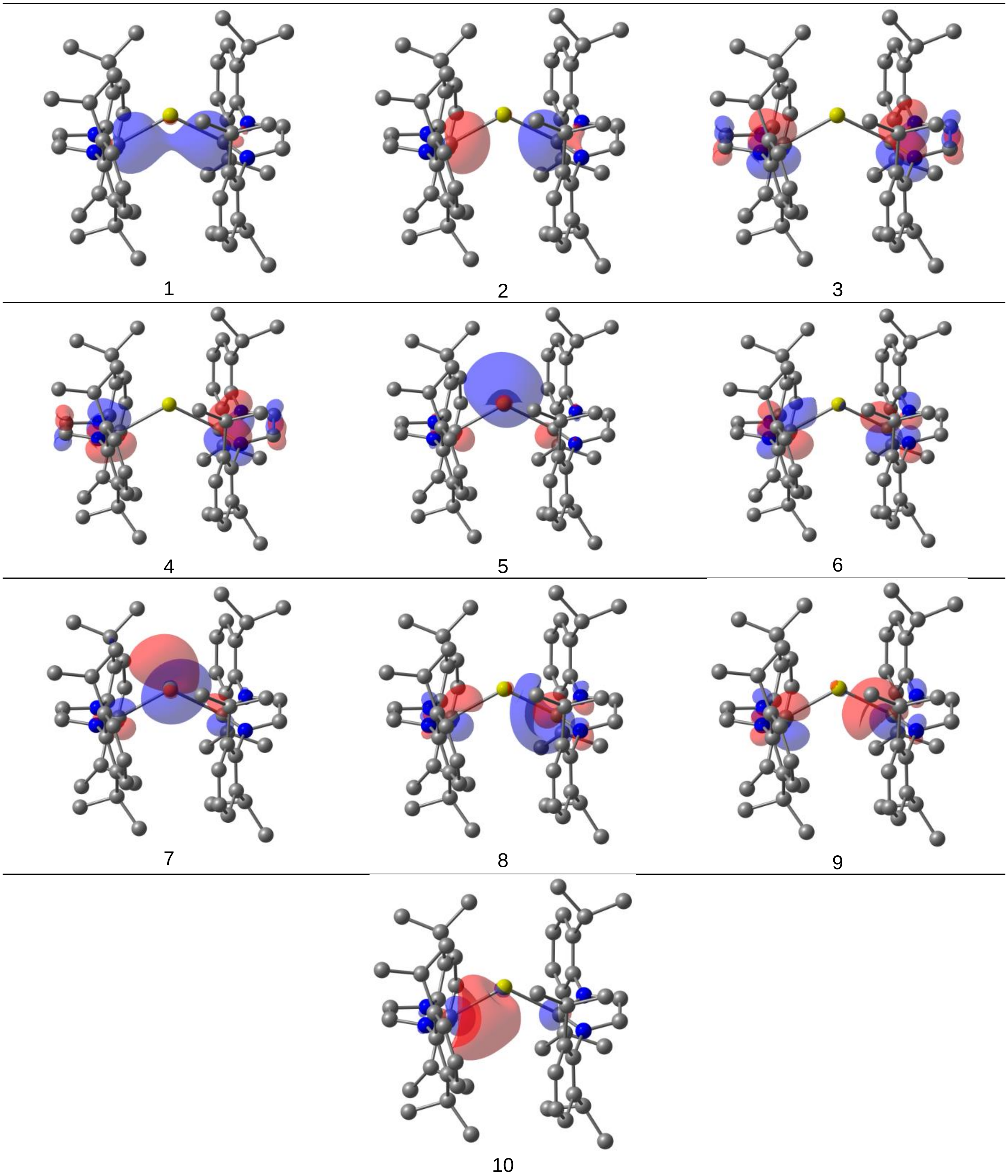

CI Coefficients: (2222200000) = -0.9444, (2222002000) = 0.1636

Figure S26. Active-space molecular orbitals and CI coefficients for the singlet spin-state **Mg-NHC^{Dip}** obtained at the CASSCF(10,10)/cc-pVDZ//B3LYP-D3(BJ)/def2-SVP level of theory. Isocontour value of 0.05 a.u. Hydrogen atoms were omitted for clarity.

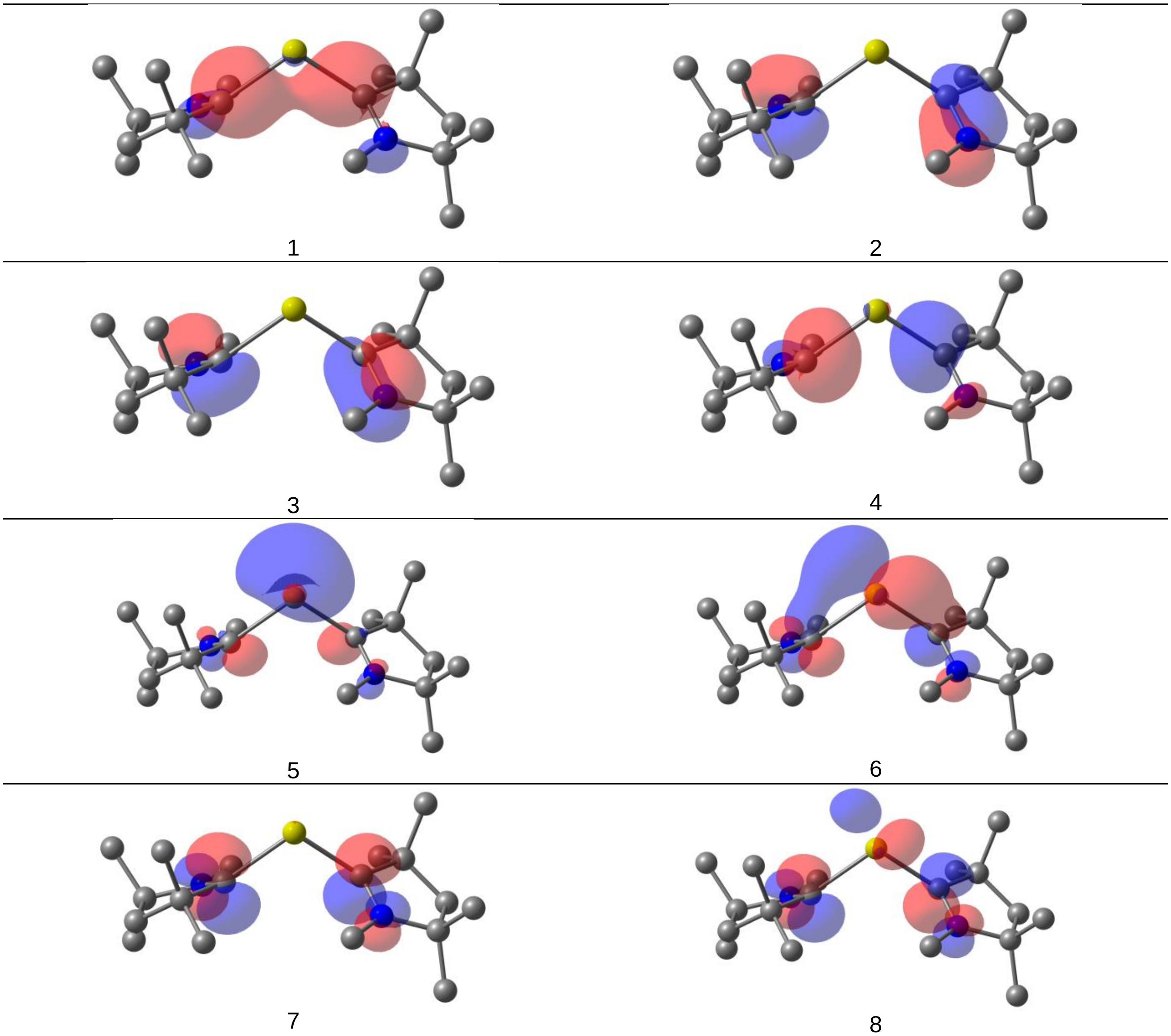

CI Coefficients: (2222200000) = 0.9273, (2222020000) = -0.2400

Figure S27. Active-space molecular orbitals and CI coefficients for the singlet spin-state **Mg-cAAC^{Me}** obtained at the CASSCF(10,8)/cc-pVDZ//B3LYP-D3(BJ)/def2-SVP level of theory. Isocontour value of 0.05 a.u. Hydrogen atoms were omitted for clarity.

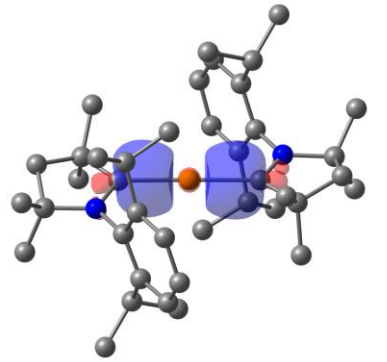

1

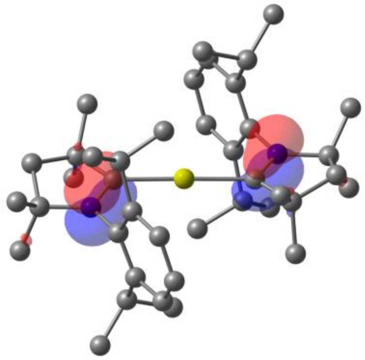

2

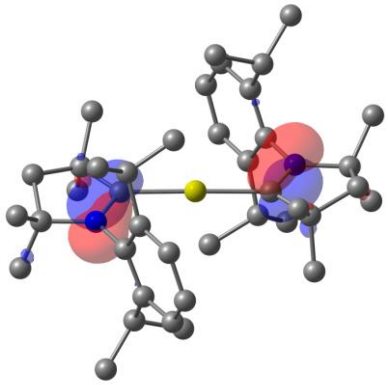

3

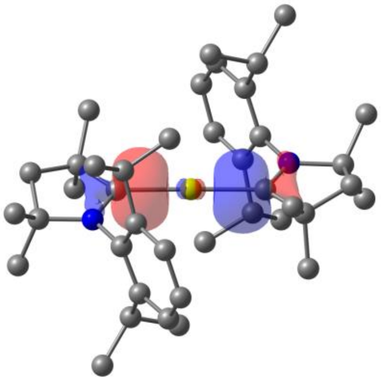

4

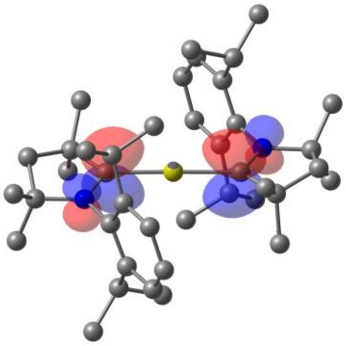

5

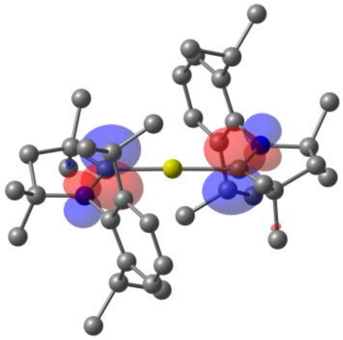

6

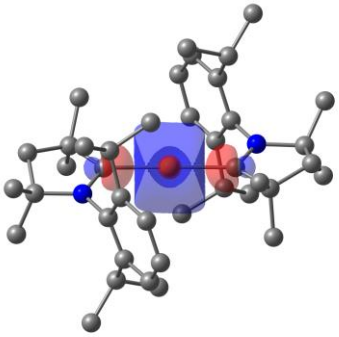

7

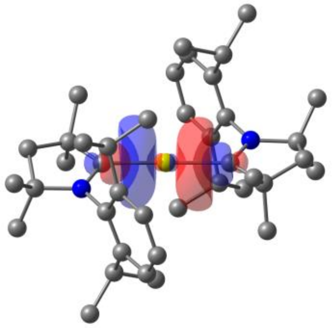

8

CI Coefficients: (2222200000) = -0.7667, (2222020000) = 0.6174

Figure S28. Active-space molecular orbitals and CI coefficients for the singlet spin-state **Mg-cAAC^{Dip}** obtained at the CASSCF(10,8)/cc-pVDZ//B3LYP-D3(BJ)/def2-SVP level of theory. Isocontour value of 0.05 a.u. Hydrogen atoms were omitted for clarity.

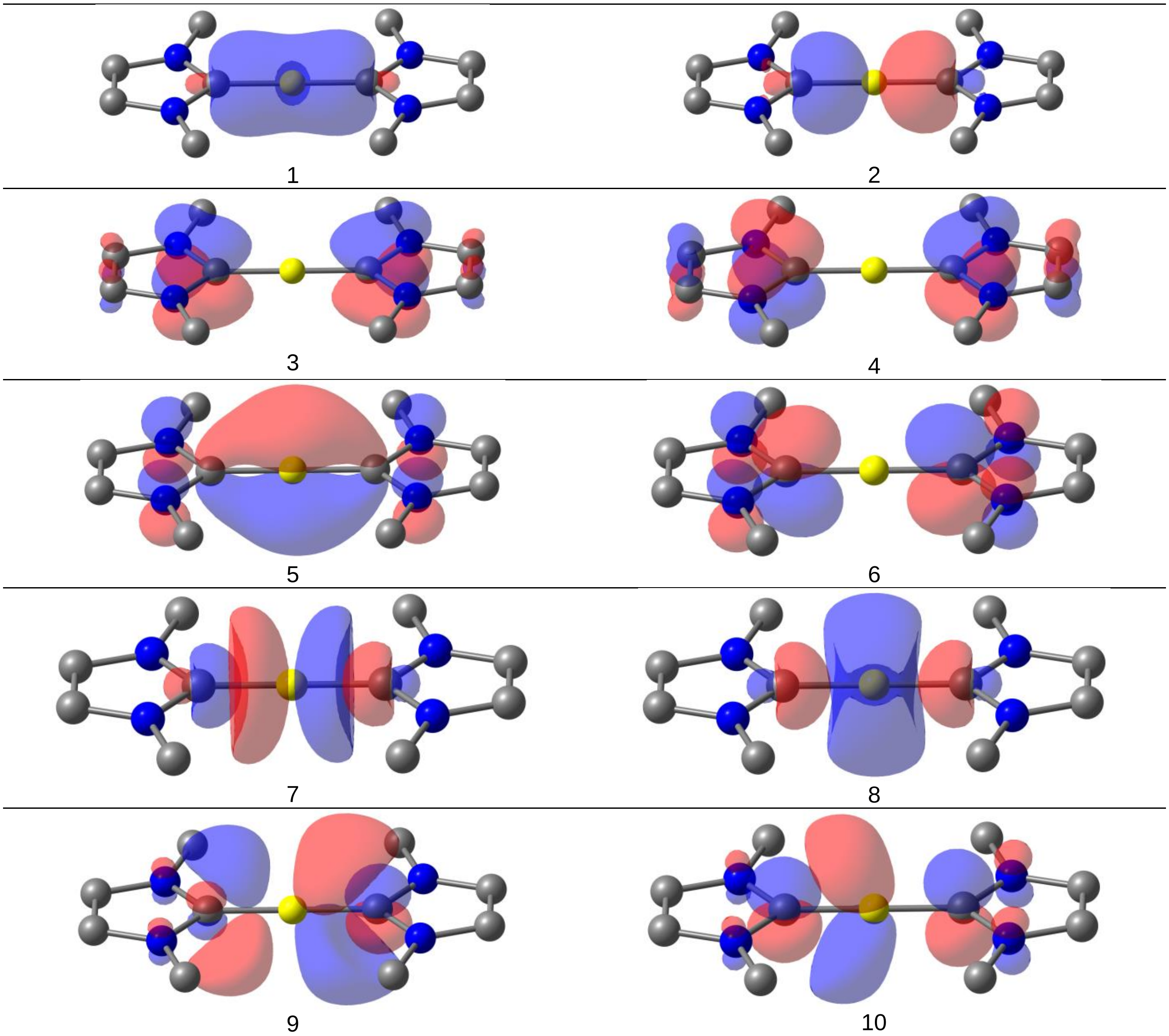

CI Coefficients: (2222200000) = -0.9406, (2222020000) = 0.2083

Figure S29. Active-space molecular orbitals and CI coefficients for the singlet spin-state **Be-NHC^{Me}** obtained at the CASSCF(10,10)/cc-pVDZ//B3LYP-D3(BJ)/def2-SVP level of theory. Isocontour value of 0.05 a.u. Hydrogen atoms were omitted for clarity.

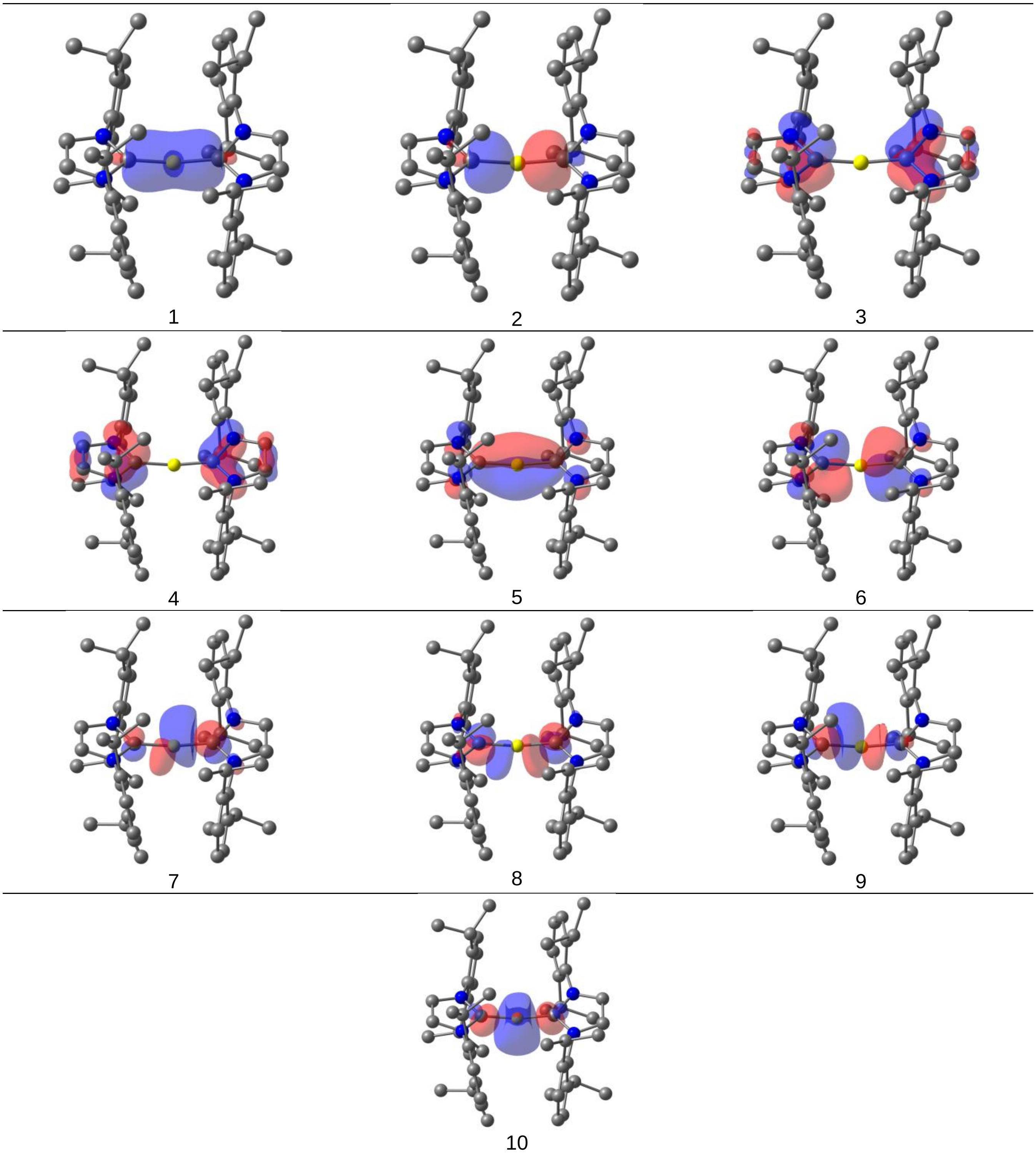

CI Coefficients: (2222200000) = 0.9283, (2222020000) = -0.2105,
(2222020000/2222000200) = -0.1058, (2222000200/2222020000) = -0.1058,

Figure S30. Active-space molecular orbitals and CI coefficients for the singlet spin-state **Be-NHC^{Dip}** obtained at the CASSCF(10,10)/cc-pVDZ//B3LYP-D3(BJ)/def2-SVP level of theory. Isocontour value of 0.05 a.u. Hydrogen atoms were omitted for clarity.

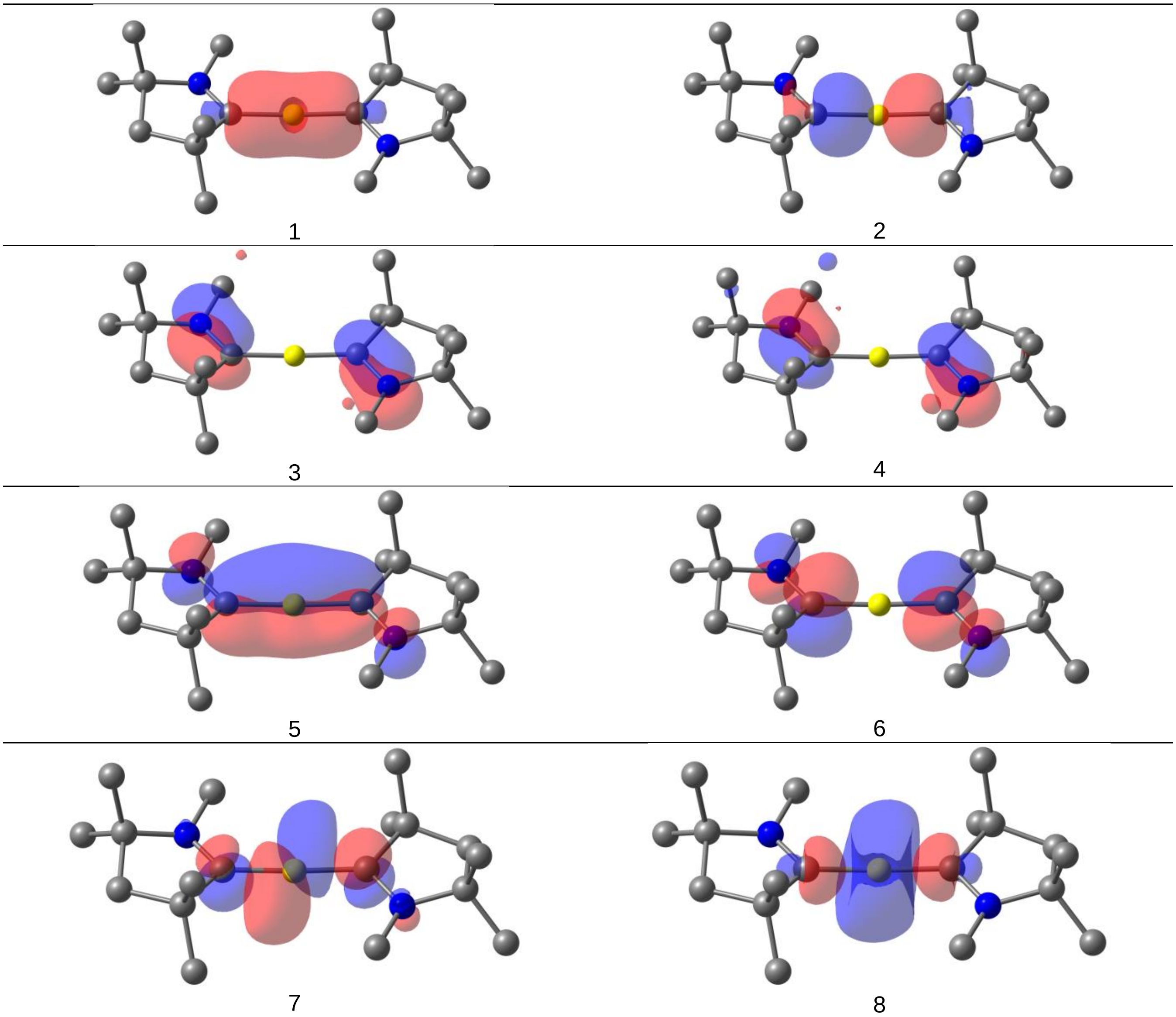

CI Coefficients: (2222200000) = 0.9189, (2222020000) = -0.3409

Figure S31. Active-space molecular orbitals and CI coefficients for the singlet spin-state **Be-cAAC^{Me}** obtained at the CASSCF(10,8)/cc-pVDZ//B3LYP-D3(BJ)/def2-SVP level of theory. Isocontour value of 0.05 a.u. Hydrogen atoms were omitted for clarity.

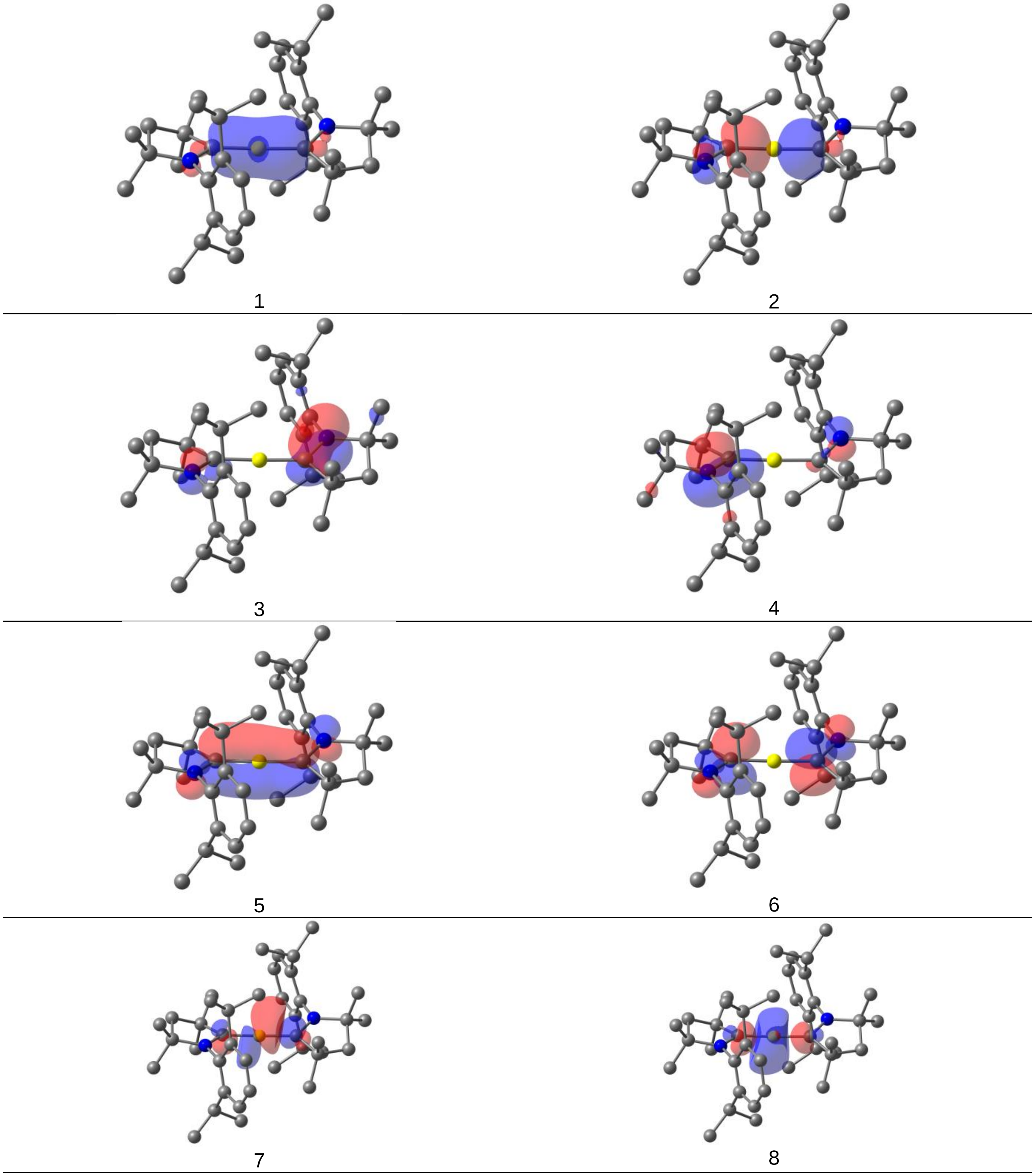

CI Coefficients: (2222200000) = -0.8960, (2222020000) = 0.4094

Figure S32. Active-space molecular orbitals and CI coefficients for the singlet spin-state **Be-cAAC^{Dip}** obtained at the CASSCF(10,8)/cc-pVDZ//B3LYP-D3(BJ)/def2-SVP level of theory. Isocontour value of 0.05 a.u. Hydrogen atoms were omitted for clarity.

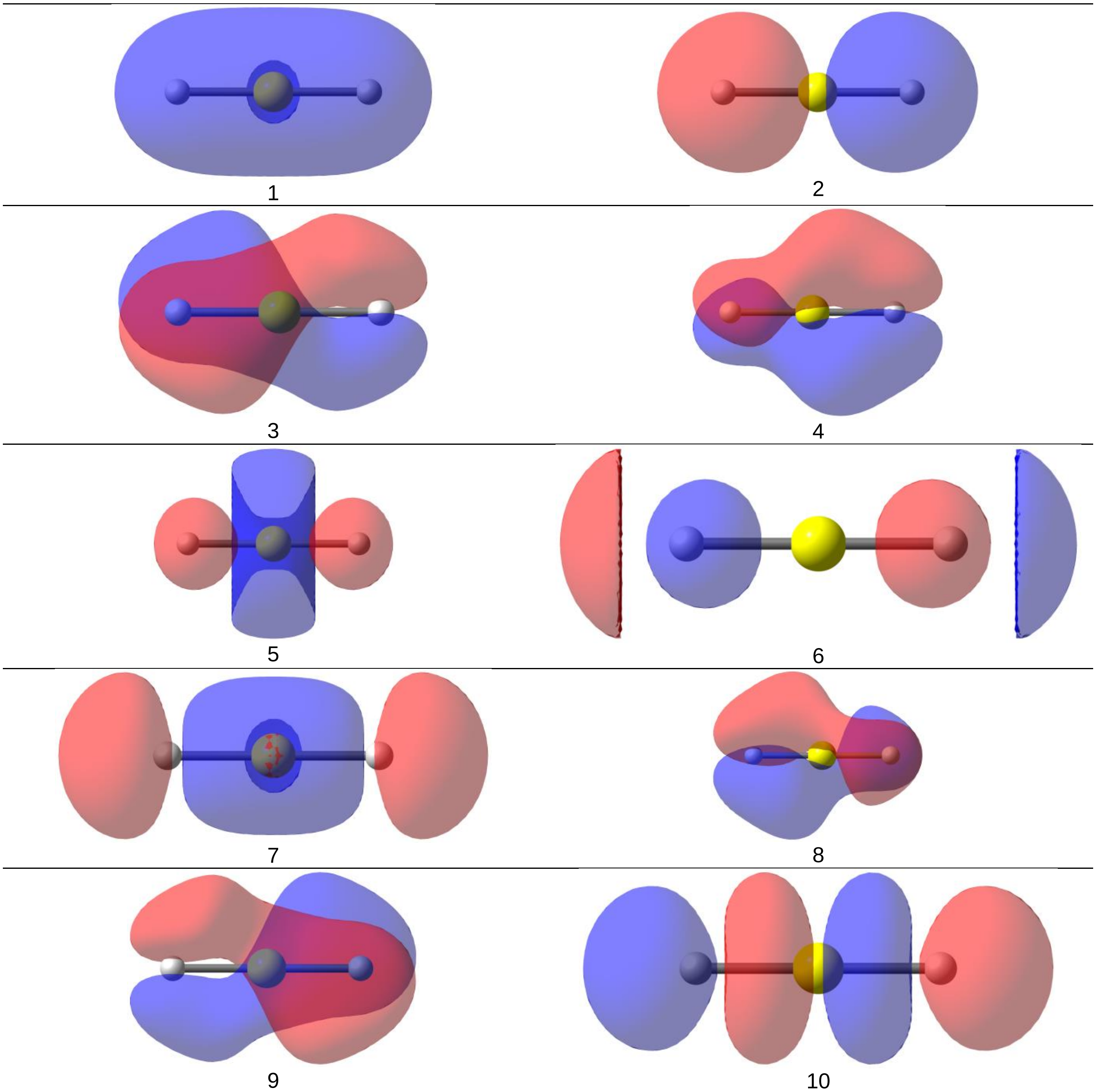

CI Coefficients: (2200000000) = 0.9799

Figure S33. Active-space molecular orbitals and CI coefficients for the singlet spin-state **Be-H_2** obtained at the CASSCF(4,10)/cc-pVDZ//B3LYP-D3(BJ)/def2-SVP level of theory. Isocontour value of 0.05 a.u.

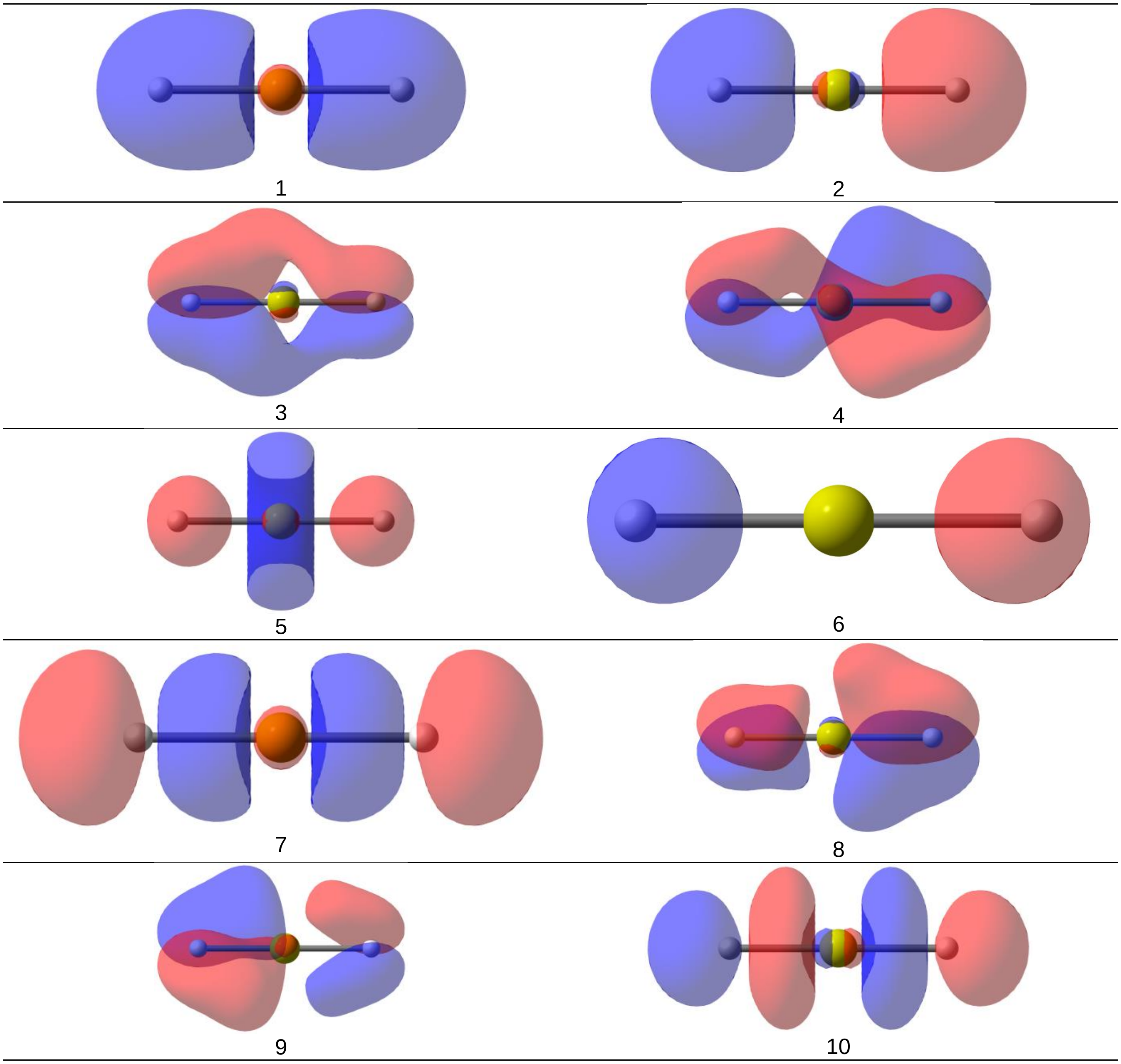

CI Coefficients: (2200000000) = 0.9774

Figure S34. Active-space molecular orbitals and CI coefficients for the singlet spin-state **Mg-H_2** obtained at the CASSCF(4,10)/cc-pVDZ//B3LYP-D3(BJ)/def2-SVP level of theory. Isocontour value of 0.05 a.u.

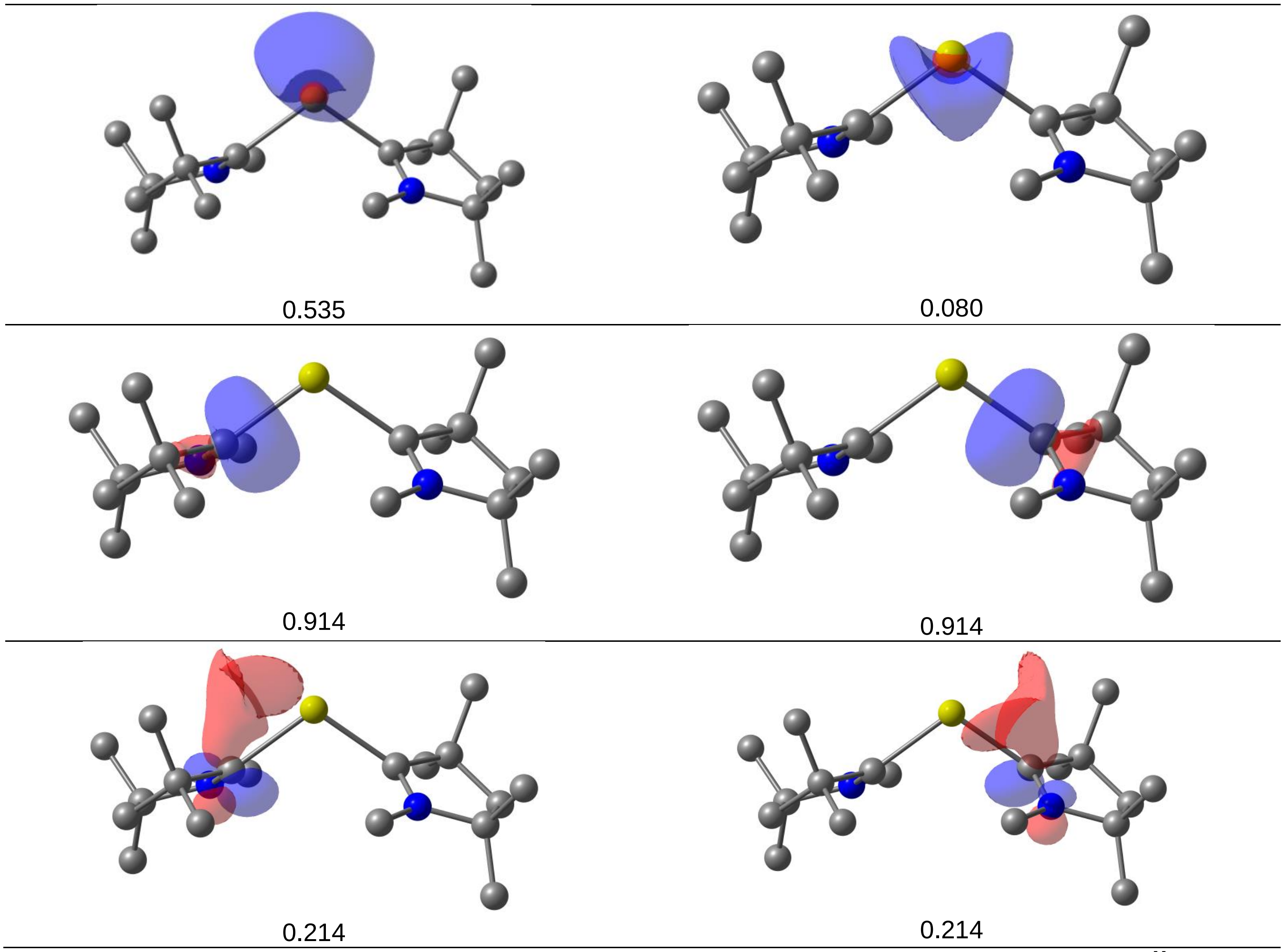

Figure S35. Relevant effective fragment orbitals (occupied in bold) for the singlet spin-state **Mg-cAAC^{Me}** obtained at the CASSCF(10,8)/cc-pVDZ//B3LYP-D3(BJ)/def2-SVP level of theory. Isocontour value of 0.075 a.u. Hydrogen atoms were omitted for clarity.

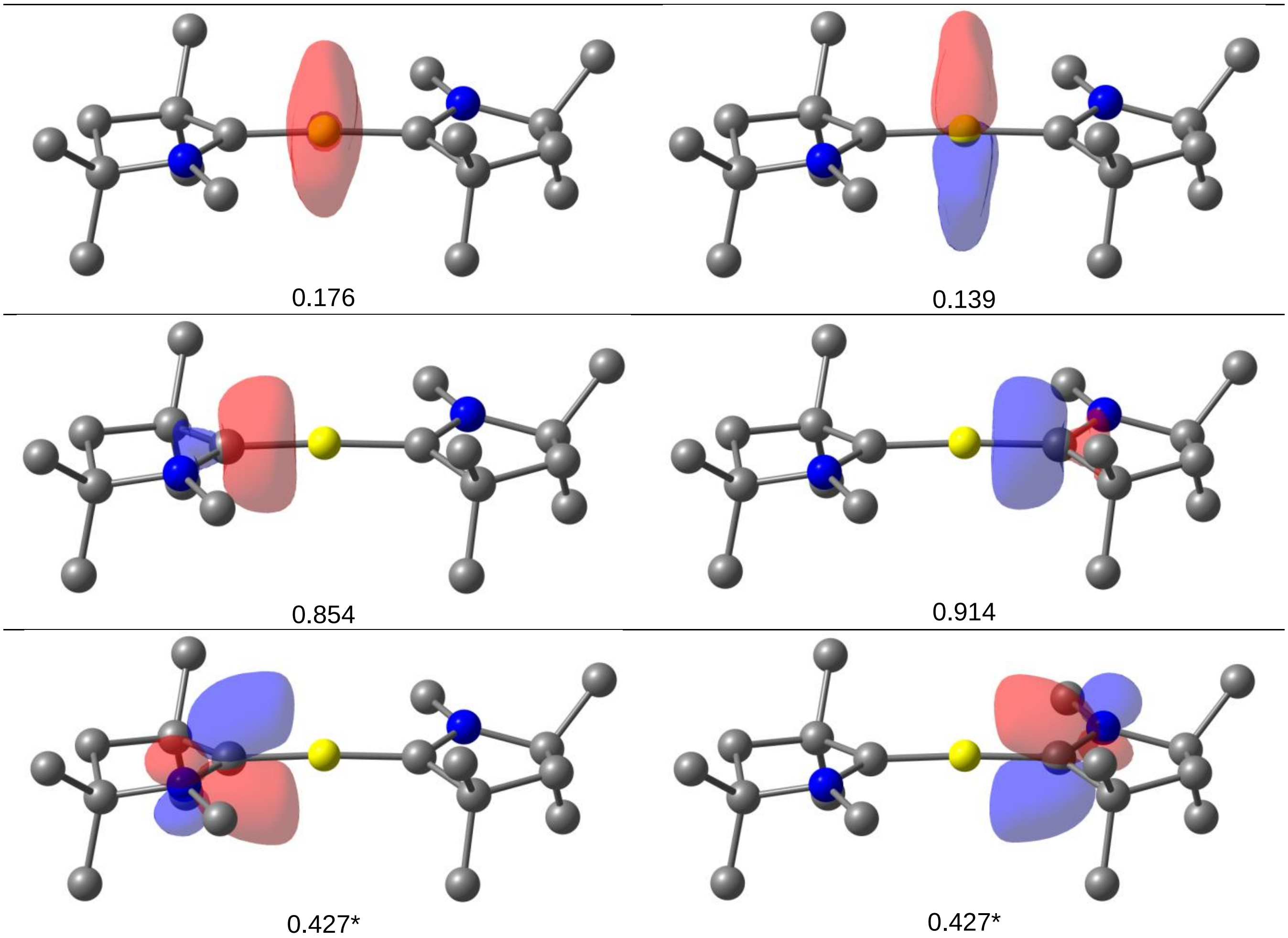

Figure S36. Relevant effective fragment orbitals (occupied in bold) for the singlet spin-state **Be-cAAC^{Me}** obtained at the CASSCF(10,8)/cc-pVDZ//B3LYP-D3(BJ)/def2-SVP level of theory. Isocontour value of 0.075 a.u. Hydrogen atoms were omitted for clarity. * Electron pair split between two pseudodegenerate (in occ.) EFOs.

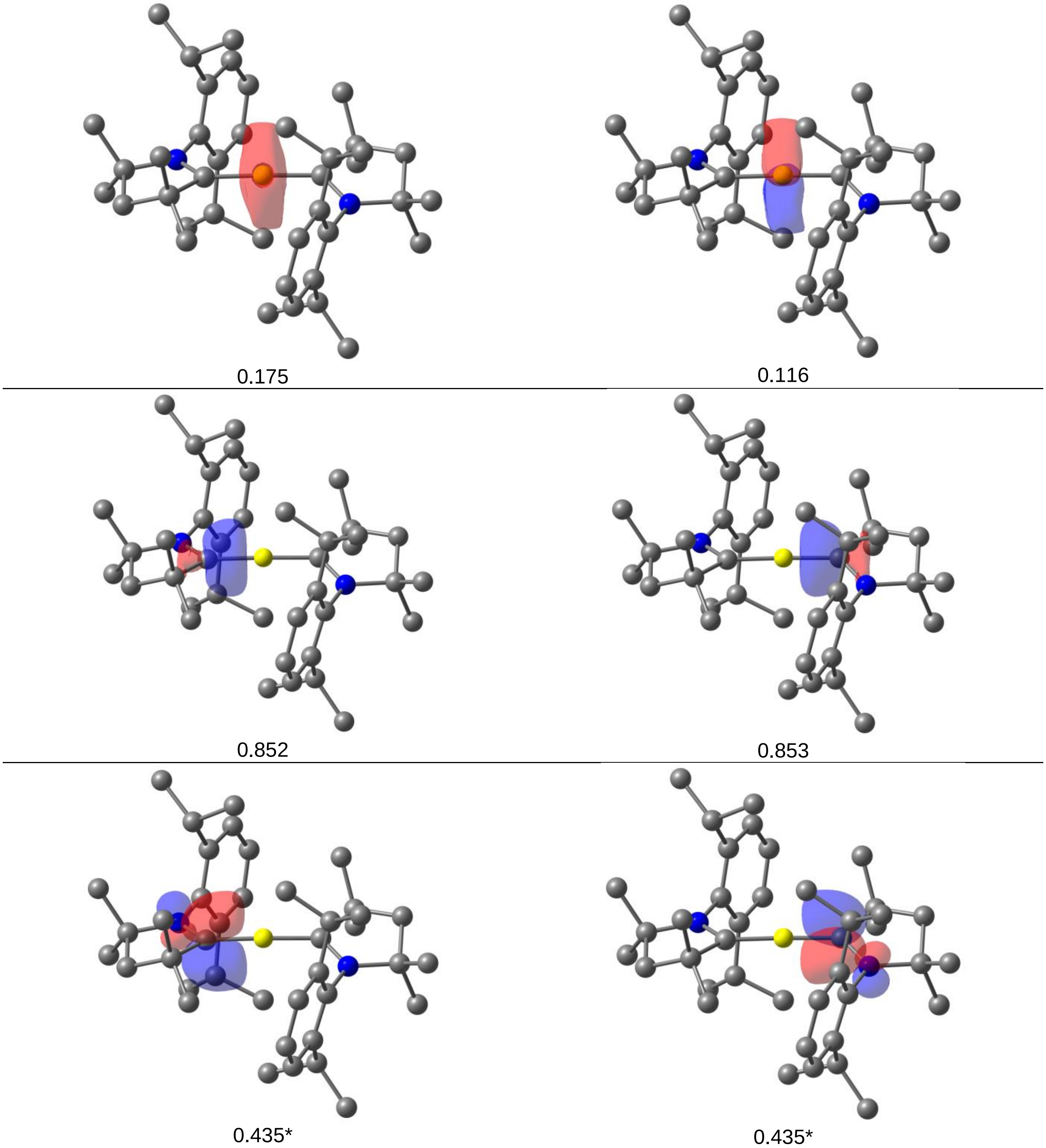

Figure S37. Relevant effective fragment orbitals (occupied in bold) for the singlet spin-state **Be-cAAC$^{\mathbf{Dip}}$** obtained at the CASSCF(10,8)/cc-pVDZ//B3LYP-D3(BJ)/def2-SVP level of theory. Isocontour value of 0.075 a.u. Hydrogen atoms were omitted for clarity. * Electron pair split between two pseudodegenerate (in occ.) EFOs.

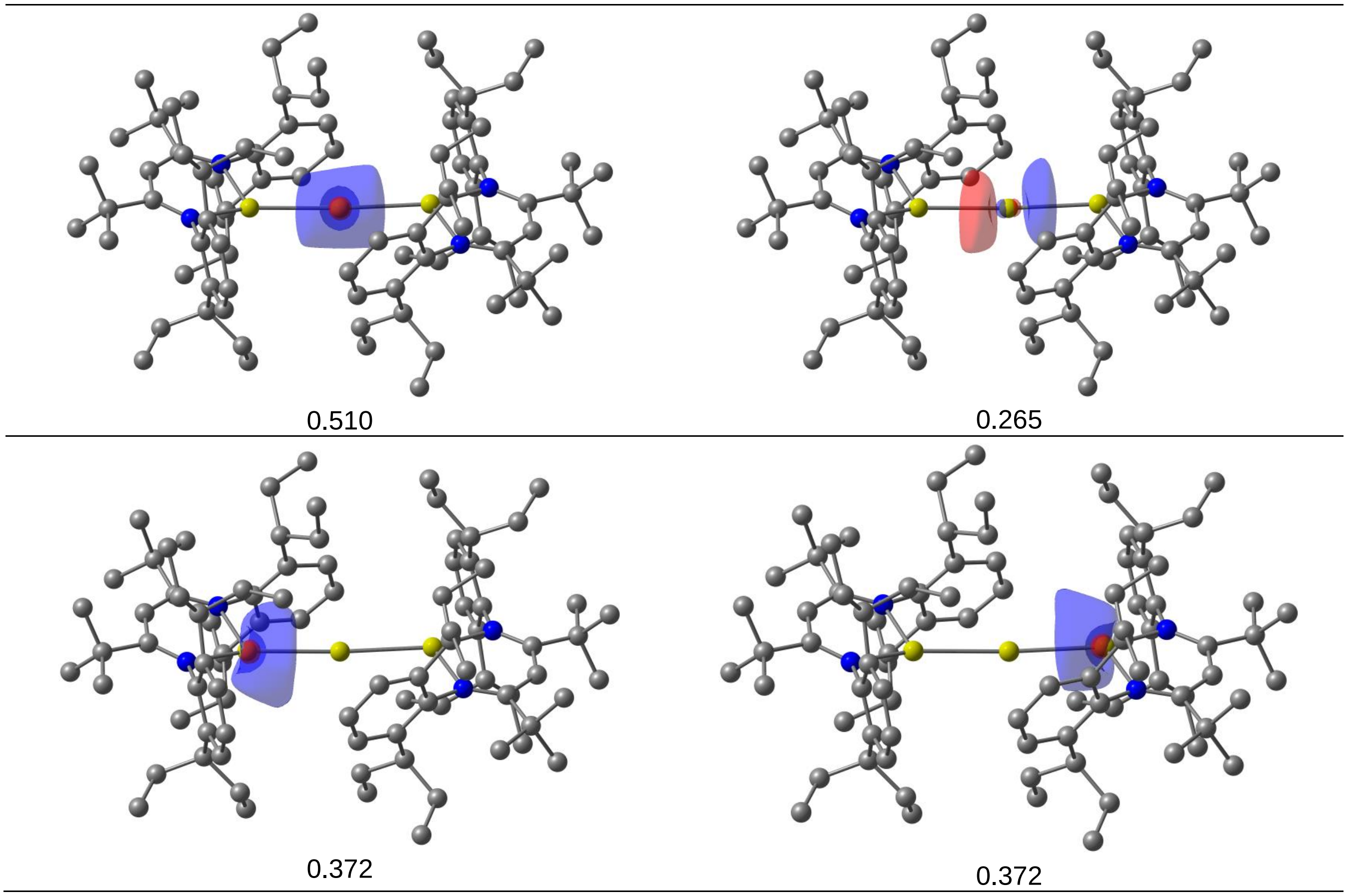

Figure S38. Relevant effective fragment orbitals (occupied in bold) for the singlet spin-state **Mg-BDI*** obtained at the B3LYP-D3(BJ)/def2-SVP level of theory. Isocontour value of 0.075 a.u. Hydrogen atoms were omitted for clarity. * Electron pair split between two pseudodegenerate (in occ.) EFOs.

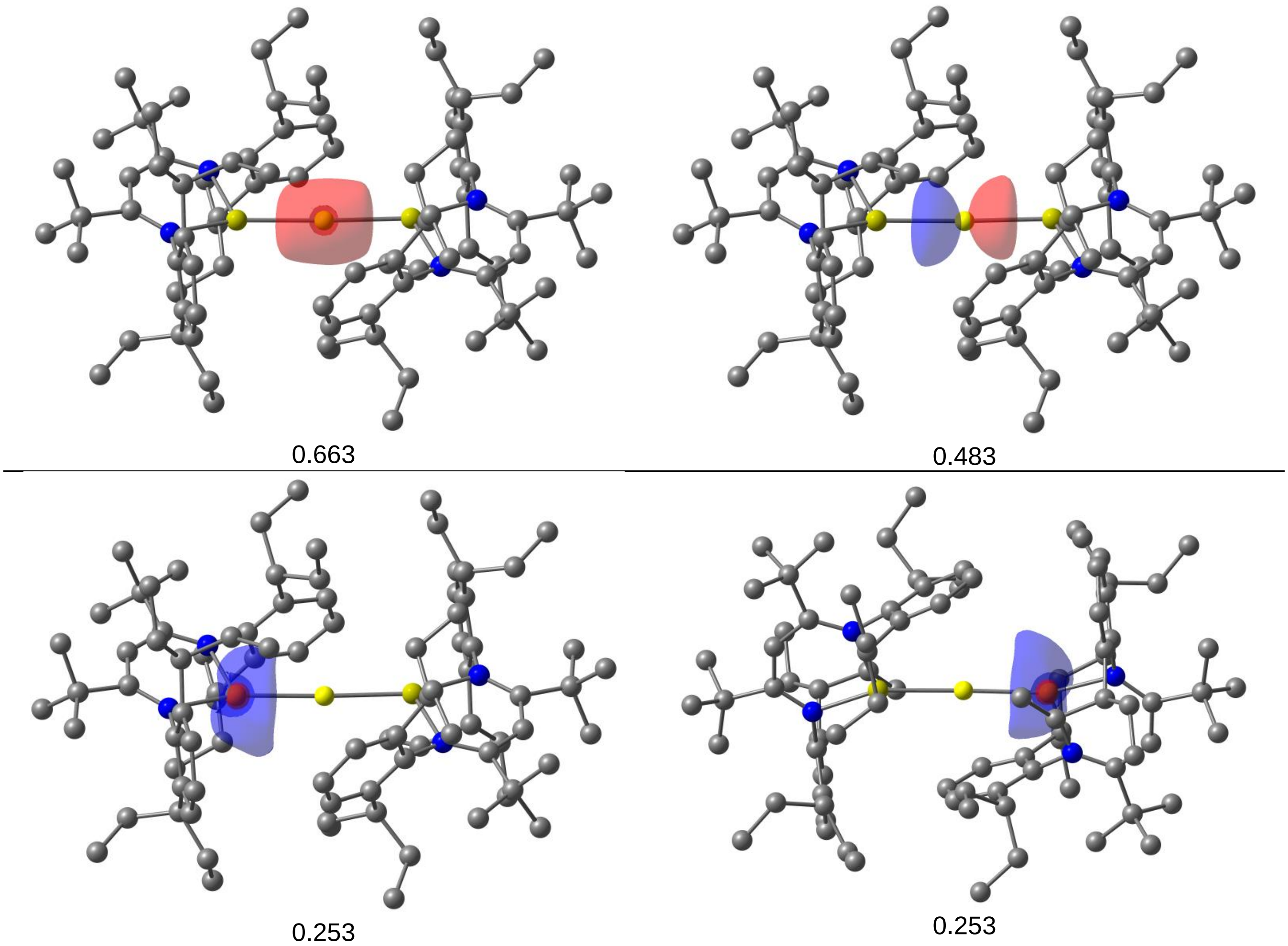

Figure S39. Relevant effective fragment orbitals (occupied in bold) for the singlet spin-state **Be-BDI*** obtained at the B3LYP-D3(BJ)/def2-SVP level of theory. Isocontour value of 0.075 a.u. Hydrogen atoms were omitted for clarity.

Table S8. EDA-NOCV of E-cAAC^{Dip} (E = Be and Mg) at the B3LYP-D3(BJ)/TZ2P level of theory. The lowest $\Delta E_{orb-corr}$ is highlighted in bold. Energy values are given in kcal/mol.

	Be-cAAC^{Dip}		Mg-cAAC^{Dip}	
E-L2 system	Be^0(^{1}D, 2s^{0}2p^2); $(cAAC)_2$ (CSS)	Be^{2+}(^{1}S, 2s^{0}2p^0); $(cAAC)_2^{2-}$ (OSS)	Mg^0 (^{1}D, 3s^{0}3p^2); $(cAAC)_2$ (CSS)	Mg^{2+} (^{1}S, 3s^{0}3p^0); $(cAAC)^{2-}$ (OSS)
ΔE_{int}	-287.1	-847.9	-222.6	-647.2
ΔE_{Pauli}	157.4	105.8	197.1	98.4
ΔE_{disp}[a]	-10.5 (2.4%)	-10.5 (1.1%)	-16.1 (3.8%)	-16.1 (2.1%)
ΔE_{elstat}[a]	-202.6 (45.6%)	-449.4 (52.4%)	-210.7 (50.2%)	-466.8 (60.4%)
ΔE_{orb}	**-231.4**	-401.4	**-193.0**	-241.6
ΔE_{orb-HF}	0.0	-42.4	-0.1	-48.2
$\Delta E_{orb-corr}$[a]	**-231.4** (52.1%)	-443.8 (46.5%)	**-193.0** (46.0%))	-289.8 (37.5%)
$\Delta E_{orb-\sigma(+,+)}$[b]	-18.3 (7.9%)	-45.7 (10.3%)	-13.8 (9.8%)	-41.9 (14.4%)
$\Delta E_{orb-\sigma(+,-)}$[b]	-51.9 (22.4%)	-90.3 (20.3%)	-18.6 (9.7%)	-28.4 (9.8%)
$\Delta E_{orb-\pi}$[b]	-150.7 (65.1%)	-211.6 (47.7%)	-152.0 (78.7%)	-119.5 (41.2%)
$\Delta E_{orb-rest}$[b]	-10.4 (4.5%)	-53.9 (12.1%)	-8.6 (4.4%)	-51.8 (17.9%)
$\langle S^2 \rangle$	0.571	0.571	0.942	0.942

[a] The value in parenthesis gives the percentage contribution to the total attractive interactions ΔE_{elstat} + ΔE_{orb} + $\Delta E_{disp.}$ [b] The values in parenthesis gives the percentage contribution to the total orbital interaction $\Delta E_{orb-corr.}$

	$Be^0(^1D)$	$(cAAC)_2$ (CSS)
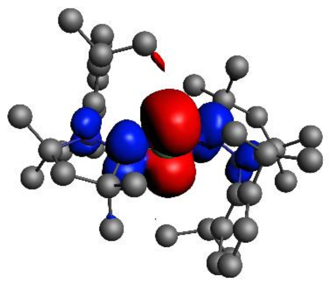	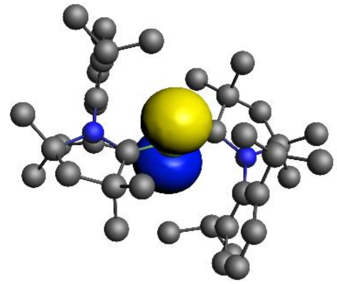	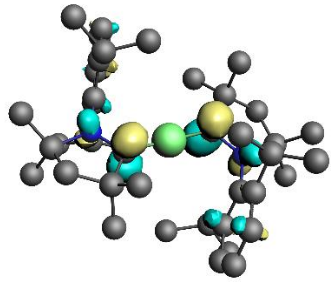
$\Delta E_{orb\text{-}\pi}$ = -150.7 ; $\lvert\upsilon_{\alpha\beta}\rvert$= 1.5	HOMO = -3.31 eV<br>υ_{α}= -0.75; υ_{β}= -0.74	LUMO = -0.64 eV<br>υ_{α}= +0.49 ; υ_{β}= +0.44
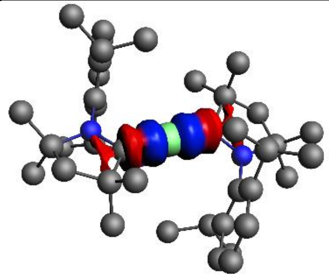	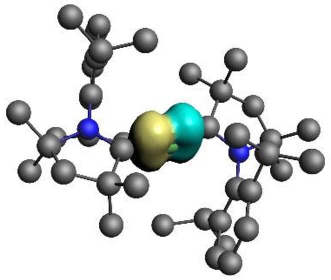	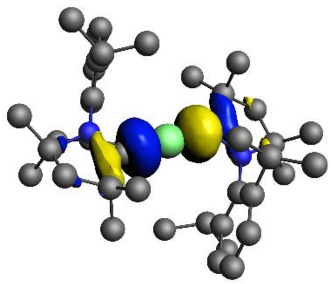
$\Delta E_{orb\text{-}\sigma(+,-)}$ = -51,9; $\lvert\upsilon_{\alpha\beta}\rvert$=0.5	LUMO+5 =36.11 eV<br>υ_{α}= +0.16; υ_{β}= +0.17	HOMO=-4.10 eV<br>υ_{α}= -0.20; υ_{β}= -0.20
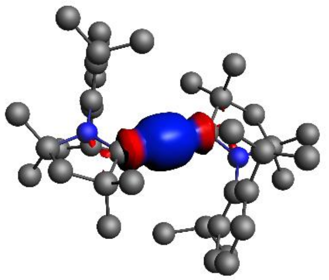	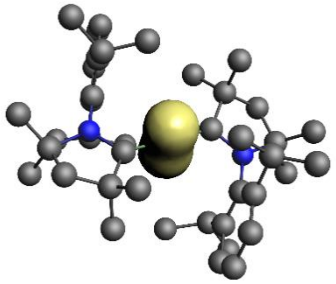	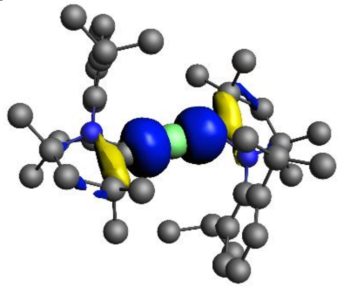
$\Delta E_{orb\text{-}\sigma(+,+)}$ = -18.3 ; $\lvert\upsilon_{\alpha\beta}\rvert$=0.3	LUMO+1=13.22 eV<br>υ_{α}= +0.10; υ_{β}= +0.10	HOMO-1=-5.29 eV<br>υ_{α}= -0.18; υ_{β}= -0.18

Figure S40. Plot of deformation densities $\Delta\rho$ (isovalue = 0.003) of the pairwise orbital interactions between $Be^0(^1D, 2s^02p^2)$ and $cAAC^{Dip}$ within Be-$cAAC^{Dip}$, associated energies ΔE (in kcal/mol) and eigenvalues v (in a.u.). The red colour shows the charge outflow, whereas blue shows charge density accumulation. Shape of the most important interacting occupied and vacant orbitals (isovalue = 0.05) of the fragments. The fragment molecular orbitals the occupied orbitals are shown in blue and yellow, while virtual orbitals are in cyan and pale yellow. Hydrogen atoms are omitted for clarity.

	$Be^{+2}(^1S)$	$(cAAC)_2^{-2}$ (OSS)
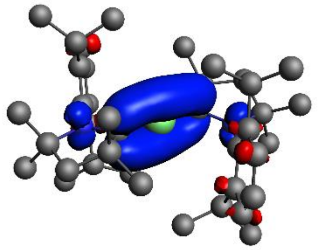	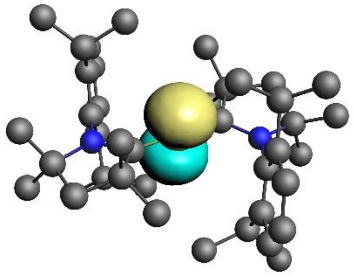	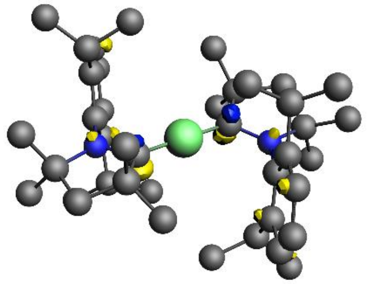
$\Delta E_{orb\text{-}\pi}$ *=-221.6 ; $\lvert\upsilon_{\alpha\beta}\rvert$= 1.8	LUMO = -6.89 eV<br>υ_α= +0.20; υ_β= +0.22	HOMO = 3.97 eV<br>υ_α= -0.84; υ_β= -0.84
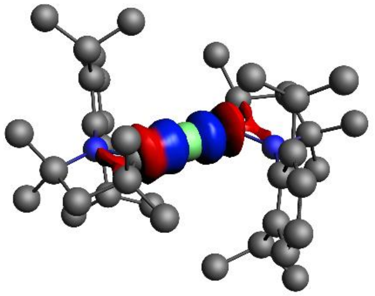	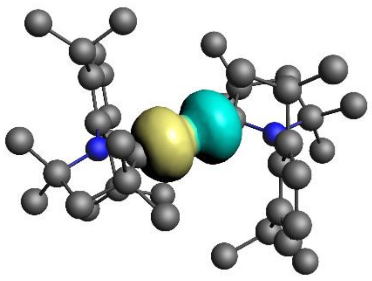	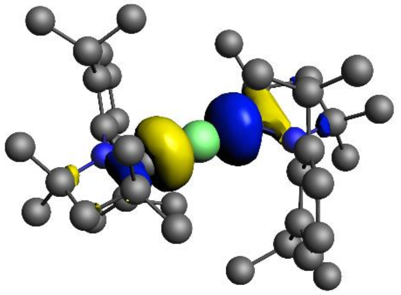
$\Delta E_{orb\text{-}\sigma(+,-)}$ = -90.3; $\lvert\upsilon_{\alpha\beta}\rvert$=0.5	LUMO = -6.89 eV<br>υ_α= +0.21; υ_β= +0.21	HOMO-1 = 1.37 eV<br>υ_α= -0.21; υ_β= -0.21
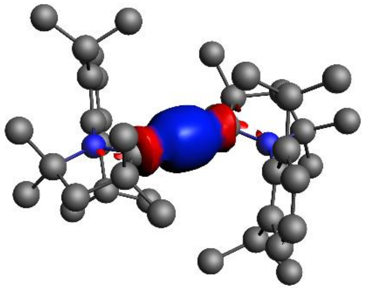	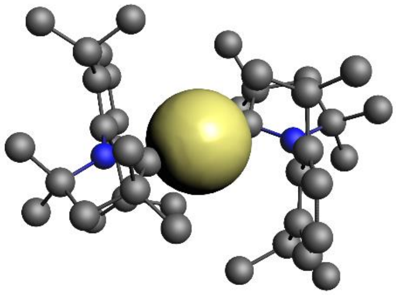	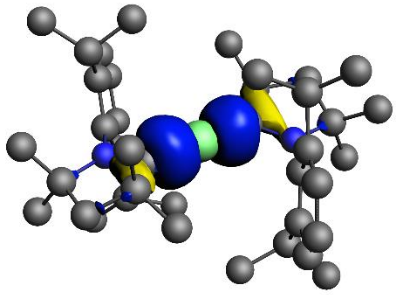
$\Delta E_{orb\text{-}\sigma(+,+)}$ =-45.7; $\lvert\upsilon_{\alpha\beta}\rvert$=0.4	LUMO = -7.64 eV<br>υ_α= +0.19; υ_β= +0.19	HOMO-2 = 0.17 eV<br>υ_α=-0.19; υ_β=-0.19

Figure S41. Plot of deformation densities $\Delta\rho$ (isovalue = 0.003) of the pairwise orbital interactions between Be^{+2}(^{1}S, $2s^02p^0$) and $(cAAC^{Dip})_2^{2-}$ within Be-cAAC^{Dip}, associated energies ΔE (in kcal/mol) and eigenvalues v (in a.u.). The red colour shows the charge outflow, whereas blue shows charge density accumulation. Shape of the most important interacting occupied and vacant orbitals (isovalue = 0.05) of the fragments. The fragment molecular orbitals the occupied orbitals are shown in blue and yellow, while virtual orbitals are in cyan and pale yellow. Hydrogen atoms are omitted for clarity.* $\Delta E_{orb\text{-}\pi}$ has high intrafragment polarization contributions.

	$Mg^0(^1D)$	$(cAAC)_2$ (CSS)
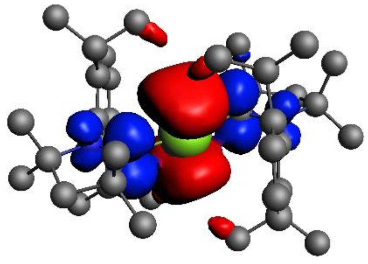	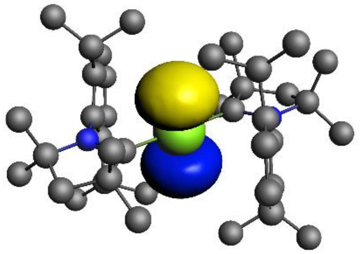	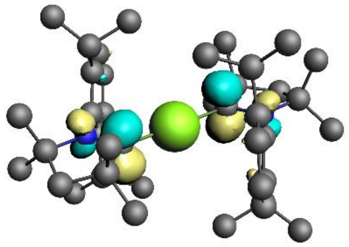
$\Delta E_{orb\text{-}\pi}$ = -152.0; $\lvert\upsilon_{\alpha\beta}\rvert$=1.7	HOMO = -2.44 eV<br>υ_α= -0.86; υ_β= -0.86	LUMO = -0.67eV<br>υ_α= +0.46; υ_β=+0.47
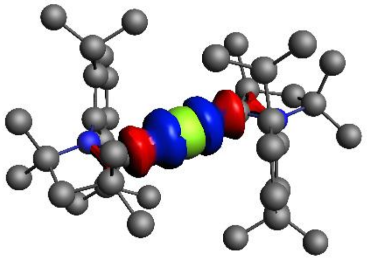	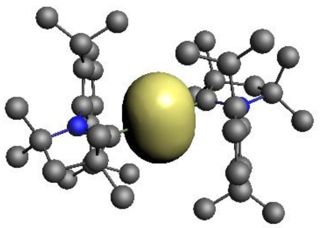	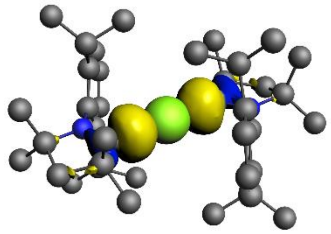
$\Delta E_{orb\text{-}\sigma(+,+)}$ = -18.6; $\lvert\upsilon_{\alpha\beta}\rvert$=0.4	LUMO = -0.60 eV<br>υ_α= +0.19; υ_β= +0.19	HOMO-1 =-5.15 eV<br>υ_α= -0.23; υ_β= -0.23
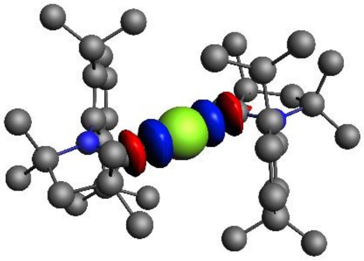	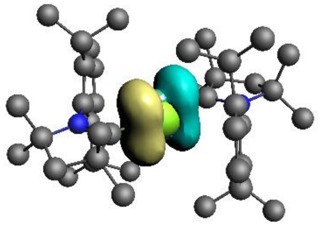	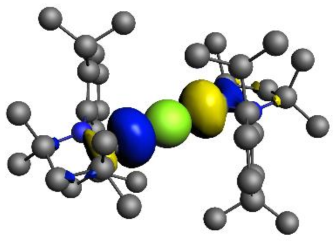
$\Delta E_{orb\text{-}\sigma(+,-)}$ = -13.8; $\lvert\upsilon_{\alpha\beta}\rvert$=0.3	LUMO +2= 7.42 eV<br>υ_α= +0.12; υ_β= +0.12	HOMO = -4.75 eV<br>υ_α= -0.16; υ_β= -0.16

Figure S42. Plot of deformation densities $\Delta\rho$ (isovalue = 0.003) of the pairwise orbital interactions between Mg^0 (^1D, $3s^04p^2$) and $(cAAC^{Dip})_2$ within Mg-cAAC^{Dip}, associated energies ΔE (in kcal/mol) and eigenvalues ν (in a.u.). The red colour shows the charge outflow, whereas blue shows charge density accumulation. Shape of the most important interacting occupied and vacant orbitals (isovalue = 0.05) of the fragments. The fragment molecular orbitals the occupied orbitals are shown in blue and yellow, while virtual orbitals are in cyan and pale yellow. Hydrogen atoms are omitted for clarity.

	$Mg^{+2}(^1S)$	$(cAAC)_2^{-2}$ (OSS)
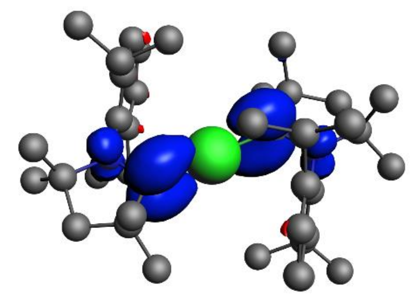	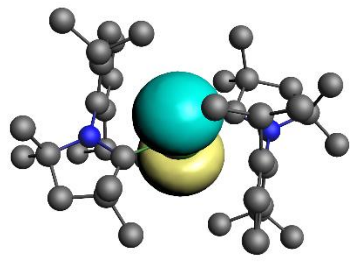	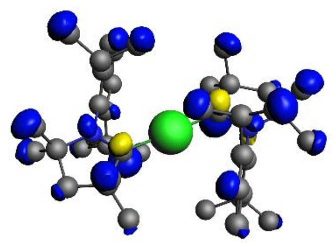
$\Delta E_{orb\text{-}\pi}$ = -119.5 ; $\lvert\upsilon_{\alpha\beta}\rvert$= 1.8	LUMO +1 = -0.47 eV<br>υ_α= +0.11; υ_β= +0.11	HOMO = 0.15 eV<br>υ_α=-0.83; υ_β=-0.83
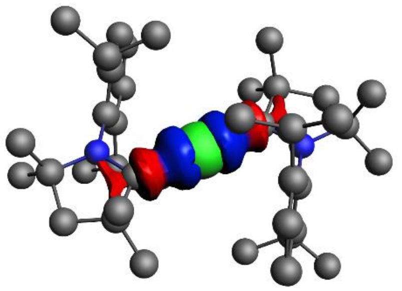	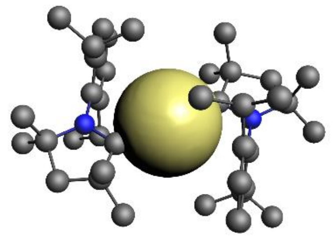	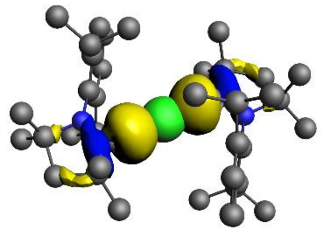
$\Delta E_{orb\text{-}\sigma(+,+)}$ = -41.9; $\lvert\upsilon_{\alpha\beta}\rvert$=0.4	LUMO = -0.67 eV<br>υ_α= +0.25; υ_β= +0.25	HOMO-2 = 0.01 eV<br>υ_α=-0.23; υ_β=-0.23
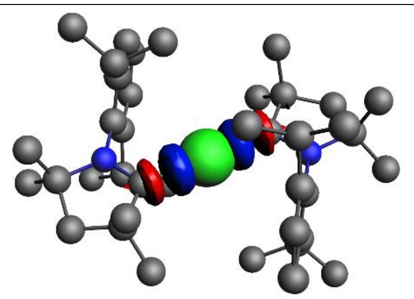	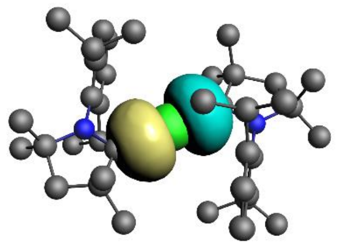	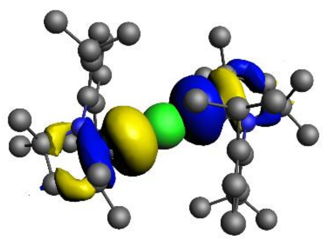
$\Delta E_{orb\text{-}\sigma(+,-)}$ = -28.4; $\lvert\upsilon_{\alpha\beta}\rvert$=0.3	LUMO+1 = -0.47 eV<br>υ_α= +0.18; υ_β= +0.18	HOMO-1 = 0.03 eV<br>υ_α=-0.16; υ_β=-0.16

Figure S43. Plot of deformation densities $\Delta\rho$ (isovalue = 0.003) of the pairwise orbital interactions between $Mg^{+2}(^1S, 3s^0 3p^2)$ and $(cAAC^{Dip})_2^{-2}$ within Mg-cAAC^{Dip}, associated energies ΔE (in kcal/mol) and eigenvalues v (in a.u.). The red colour shows the charge outflow, whereas blue shows charge density accumulation. Shape of the most important interacting occupied and vacant orbitals (isovalue = 0.05) of the fragments. The fragment molecular orbitals the occupied orbitals are shown in blue and yellow, while virtual orbitals are in cyan and pale yellow. Hydrogen atoms are omitted for clarity. * $\Delta E_{orb\text{-}\pi}$ has high intrafragment polarization contributions.

Table S9. Central element (E) atomic charges and EOS results for a selected variety of Hilbert- and real-space atomic definitions. Wavefunctions evaluated (singlet spin-state) at the CASSCF/cc-pVDZ//B3LYP-D3(BJ)/def2-SVP level of theory.

**Be-NHC^{Me}**	**E Charge**	**E EFO occ.**	**L EFO occ.**	**E OS**	**L OS**	**R (%)**
LOWDIN	-0.670	0.455	0.276	0	0	67.8
NAO	1.193	0.195	0.414	2	-1	71.9
QTAIM	1.496	0.119	0.437	2	-1	81.8
TFVC	1.099	0.141	0.382	2	-1	74.0
**Mg-NHC^{Me}**	**E Charge**	**E EFO occ.**	**L EFO occ.**	**E OS**	**L OS**	**R (%)**
LOWDIN	-0.743	0.828	0.061	0	0	100.0
NAO	0.141	0.853	0.059	0	0	100.0
QTAIM	0.196	0.760	0.093	0	0	100.0
TFVC	0.252	0.631	0.112	0	0	100.0
**Be-cAAC^{Me}**	**E Charge**	**E EFO occ.**	**L EFO occ.**	**E OS**	**L OS**	**R (%)**
LOWDIN	-0.470	0.369	0.334	0	0	53.6
NAO	1.350	0.205	0.455	2	-1	75.0
QTAIM	1.585	0.088	0.456	2	-1	86.9
TFVC	1.167	0.133	0.411	2	-1	77.8
**Mg-cAAC^{Me}**	**E Charge**	**E EFO occ.**	**L EFO occ.**	**E OS**	**L OS**	**R (%)**
LOWDIN	-0.753	0.749	0.106	0	0	99.6
NAO	0.359	0.751	0.120	0	0	100.0
QTAIM	0.391	0.655	0.153	0	0	100.0
TFVC	0.602	0.489	0.188	0	0	80.0

Table S10. Central element (E) atomic charges and EOS results for a selected variety of Hilbert- and real-space atomic definitions. Wavefunctions evaluated at the B3LYP/def2-TZVPP//B3LYP-D3(BJ)/def2-SVP level of theory. SCS = Singlet closed-shell, OSS = Singlet open-shell

**Be-NHC^{Me} (SCS)**	**E Charge**	**E EFO occ.**	**L EFO occ.**	**E OS**	**L OS**	**R (%)**
LOWDIN	-0.224	0.383	0.316	0	0	56.7
NAO	1.178	0.203	0.403	2	-1	70.1
QTAIM	1.483	0.123	0.442	2	-1	81.9
TFVC	1.118	0.138	0.387	2	-1	74.9
**Mg-NHC^{Me} (SCS)**	**E Charge**	**E EFO occ.**	**L EFO occ.**	**E OS**	**L OS**	**R (%)**
LOWDIN	-0.333	0.731	0.137	0	0	100.0
NAO	0.351	0.813	0.110	0	0	100.0
QTAIM	0.394	0.712	0.151	0	0	100.0
TFVC	0.510	0.591	0.165	0	0	92.6
**Be-cAAC^{Me} (SOS)**	**E Charge**	**E EFO occ.**	**L EFO occ.**	**E OS**	**L OS**	**R (%)**
LOWDIN	-0.069	0.292	0.598	2	-1	80.6
NAO	1.403	0.196	0.748	2	-1	100.0
QTAIM	1.567	0.087	0.736	2	-1	100.0
TFVC	1.157	0.130	0.678	2	-1	100.0
**Mg-cAAC^{Me} (SOS)**	**E Charge**	**E EFO occ.**	**L EFO occ.**	**E OS**	**L OS**	**R (%)**
LOWDIN	-0.128	0.528	0.346	0	0	68.2
NAO	0.854	0.559	0.360	0	0	69.9
QTAIM	0.825	0.482	0.387	0	0	59.5
TFVC	0.851	0.392	0.391	0	0	50.1

Table S11. Geometrical parameters and relative energies (ΔE, in kcal/mol) of the studied compounds at SS-CASPT2/cc-pVDZ level in its ground state multiplicity and triplet state (in parenthesis).

System	$d_{E\text{-}L}$ [Å]	$BA_{L\text{-}E\text{-}L}$ [°]	ΔE
Mg-NHC^{Me}	2.465<br>(2.234)	86.4<br>(99.0)	0.0<br>(8.7)
Be-NHC^{Me}	1.664<br>(1.664)	179.9<br>(180.0)	0.0<br>(-1.3)
Be-cAAC^{Me}	1.662<br>(179.7)	1.666<br>(175.9)	0.0<br>(5.8)

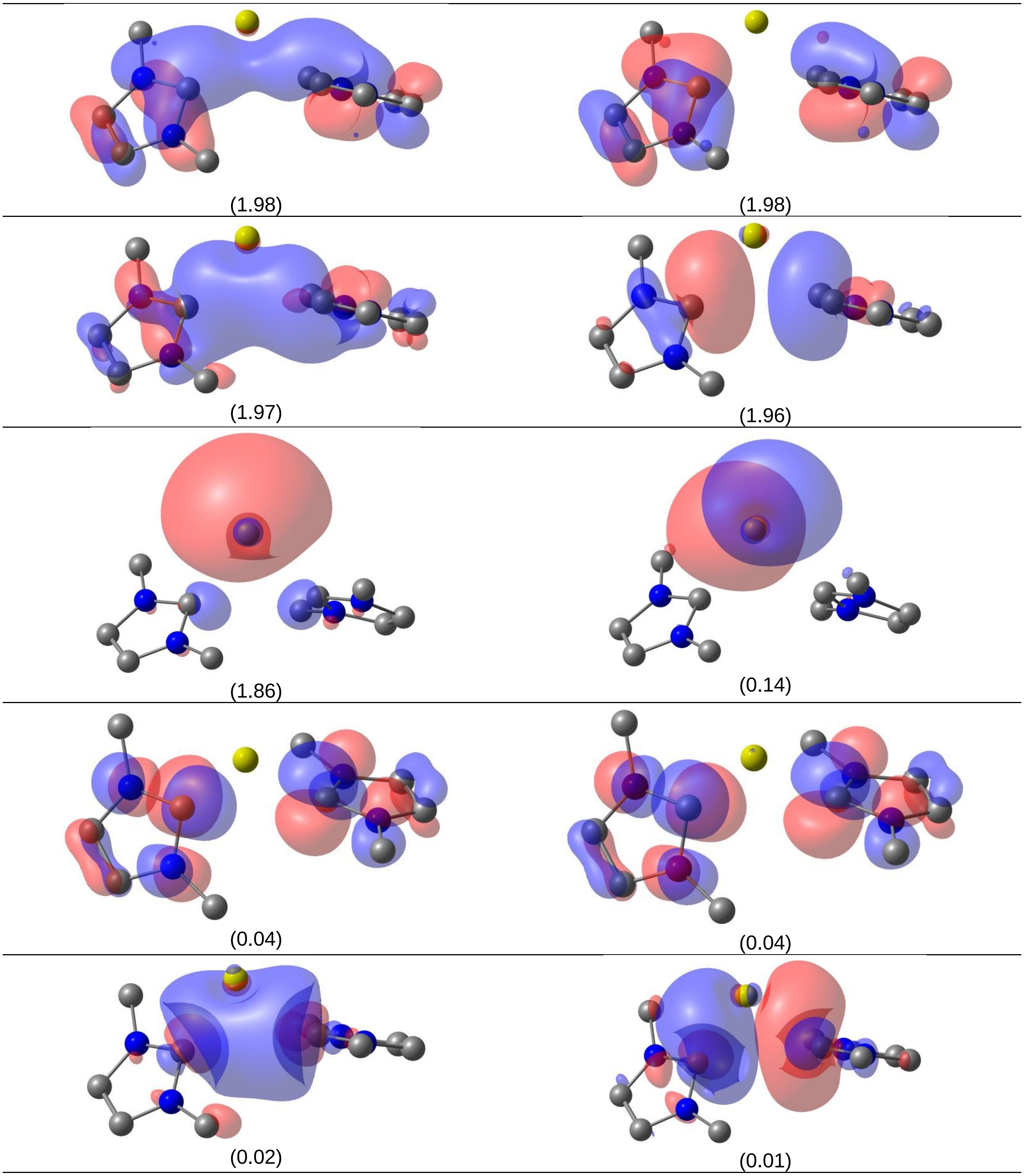

CI Coefficients: (2222200000) = 0.9376, (2222020000) = -0.2536

Figure S44. Active-space molecular orbitals with their occupancies (in e) and CI coefficients for the singlet spin-state **Mg-NHC^{Me}** obtained at the CASSCF(10,10)/cc-pVDZ//SS-CASPT2(10,10)/cc-pVDZ level of theory. Isocontour value of 0.05 a.u. Hydrogen atoms were omitted for clarity.

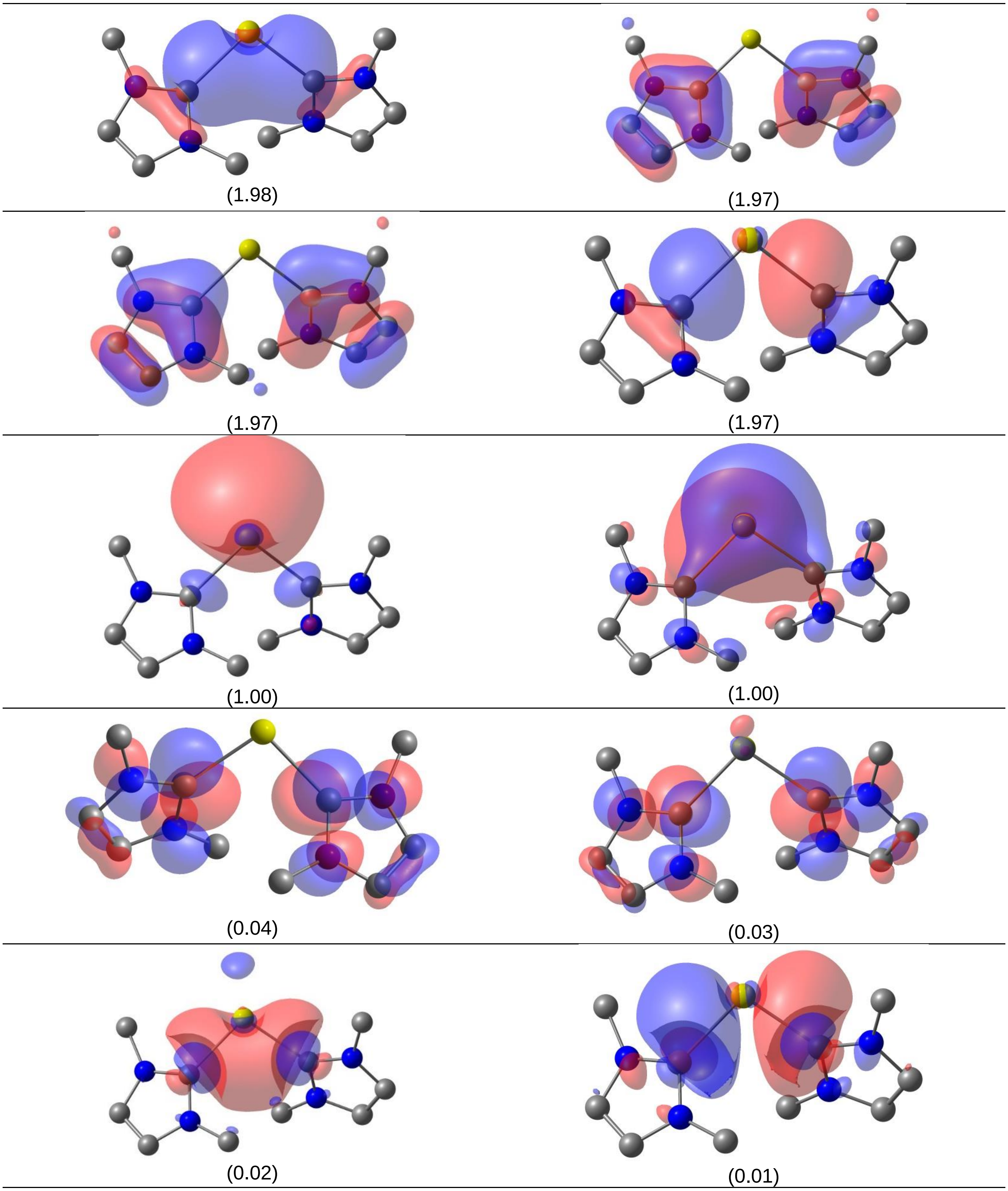

CI Coefficients: (2222110000) = 0.9714, (2202112000) = -0.054

Figure S45. Active-space molecular orbitals with their occupancies (in e) and CI coefficients for the triplet spin-state **Mg-NHC**$^{\mathbf{Me}}$ obtained at the CASSCF(10,10)/cc-pVDZ//SS-CASPT2(10,10)/cc-pVDZ level of theory. Isocontour value of 0.05 a.u. Hydrogen atoms were omitted for clarity.

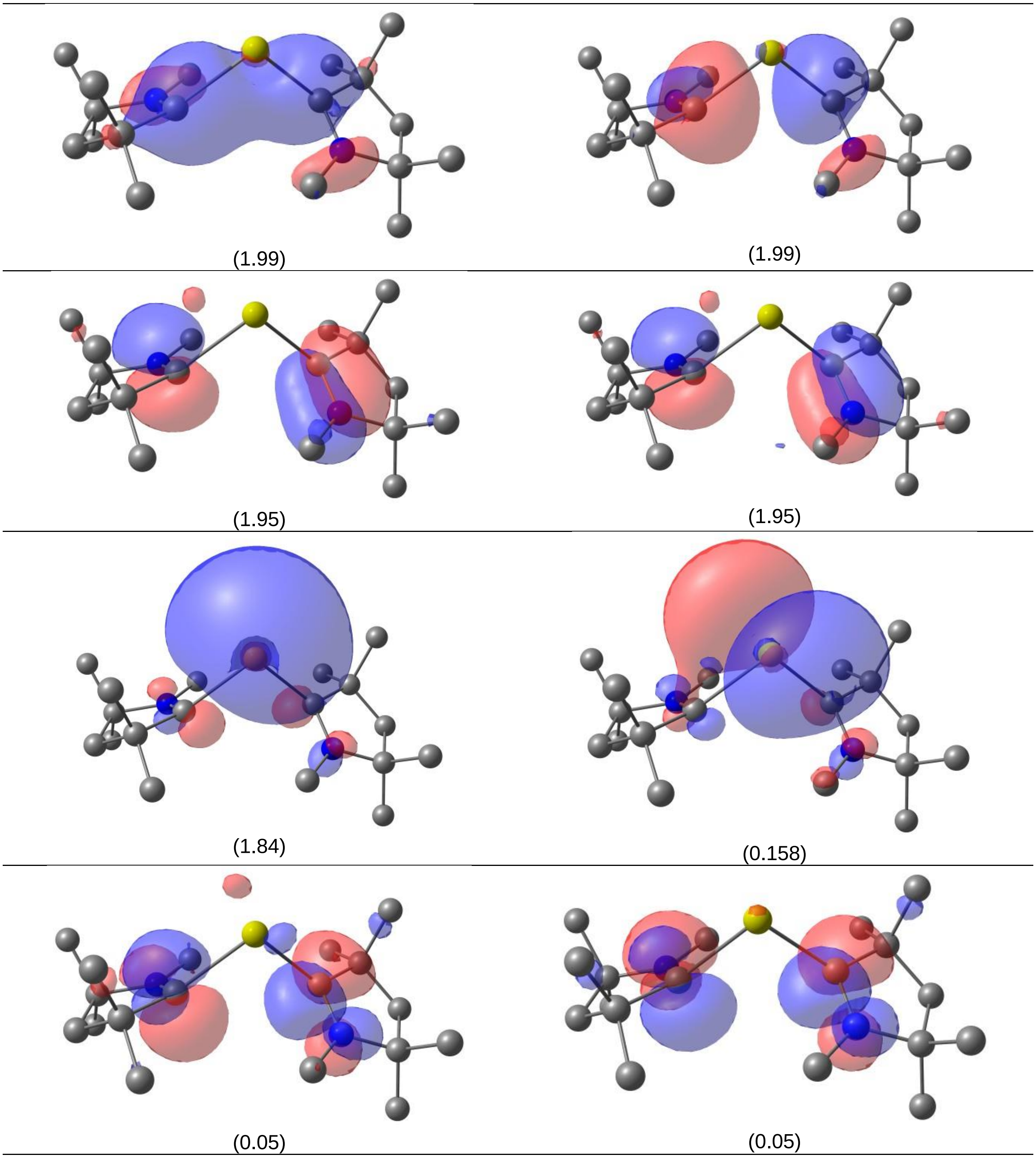

CI Coefficients: (22222000) = -0.9356, (22220200) = 0.267

Figure S46. Active-space molecular orbitals with their occupancies (in e) and CI coefficients for the singlet spin-state **Mg-cAAC^{Me}** obtained at the CASSCF(10,8)/cc-pVDZ//SS-CASPT2(10,8)/cc-pVDZ level of theory. Isocontour value of 0.05 a.u. Hydrogen atoms were omitted for clarity.

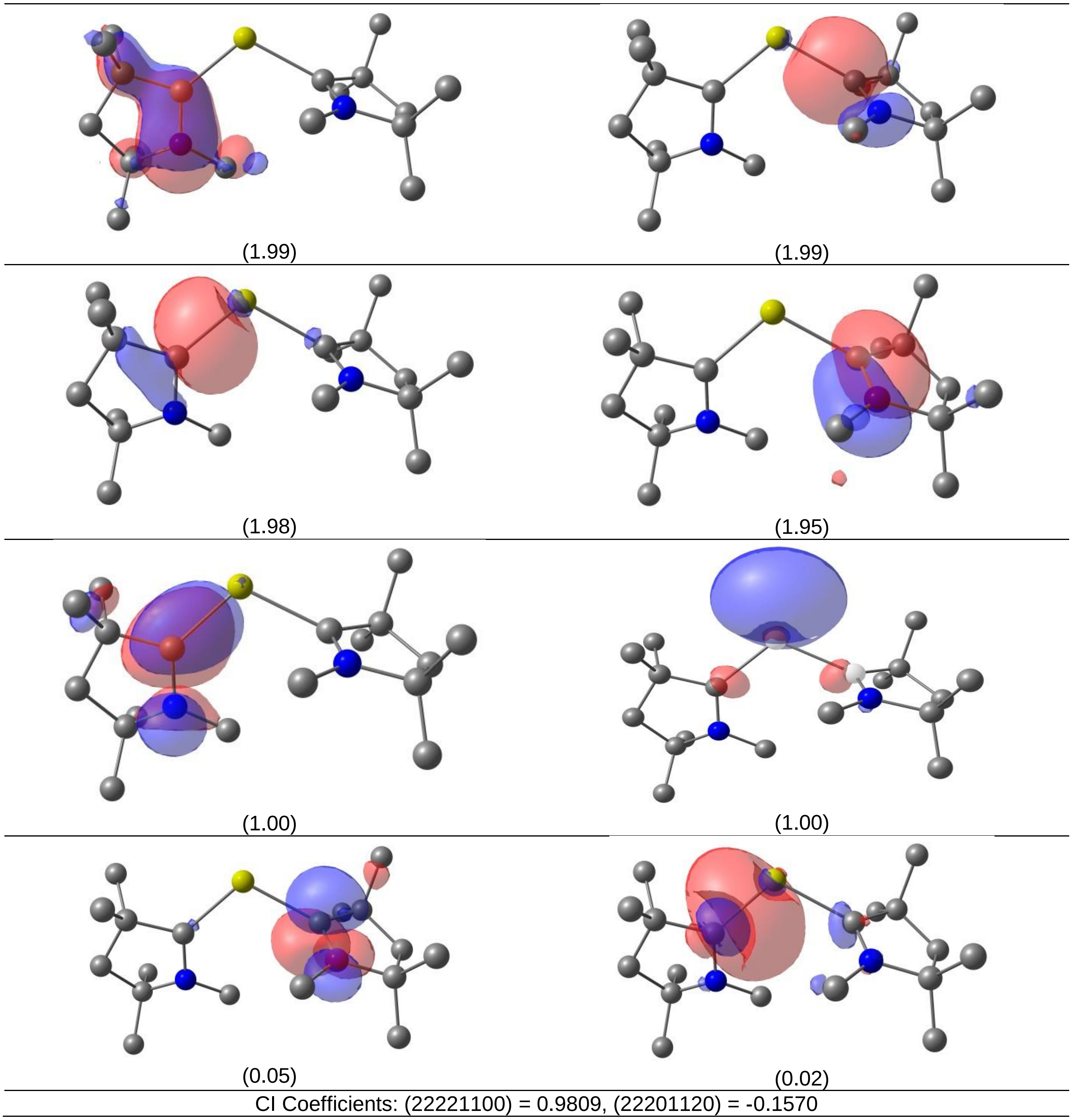

Figure S47. Active-space molecular orbitals with their occupancies (in e) and CI coefficients for the triplet spin-state **Mg-cAAC^Me^** obtained at the CASSCF(10,8)/cc-pVDZ//SS-CASPT2(10,8)/cc-pVDZ level of theory. Isocontour value of 0.05 a.u. Hydrogen atoms were omitted for clarity.

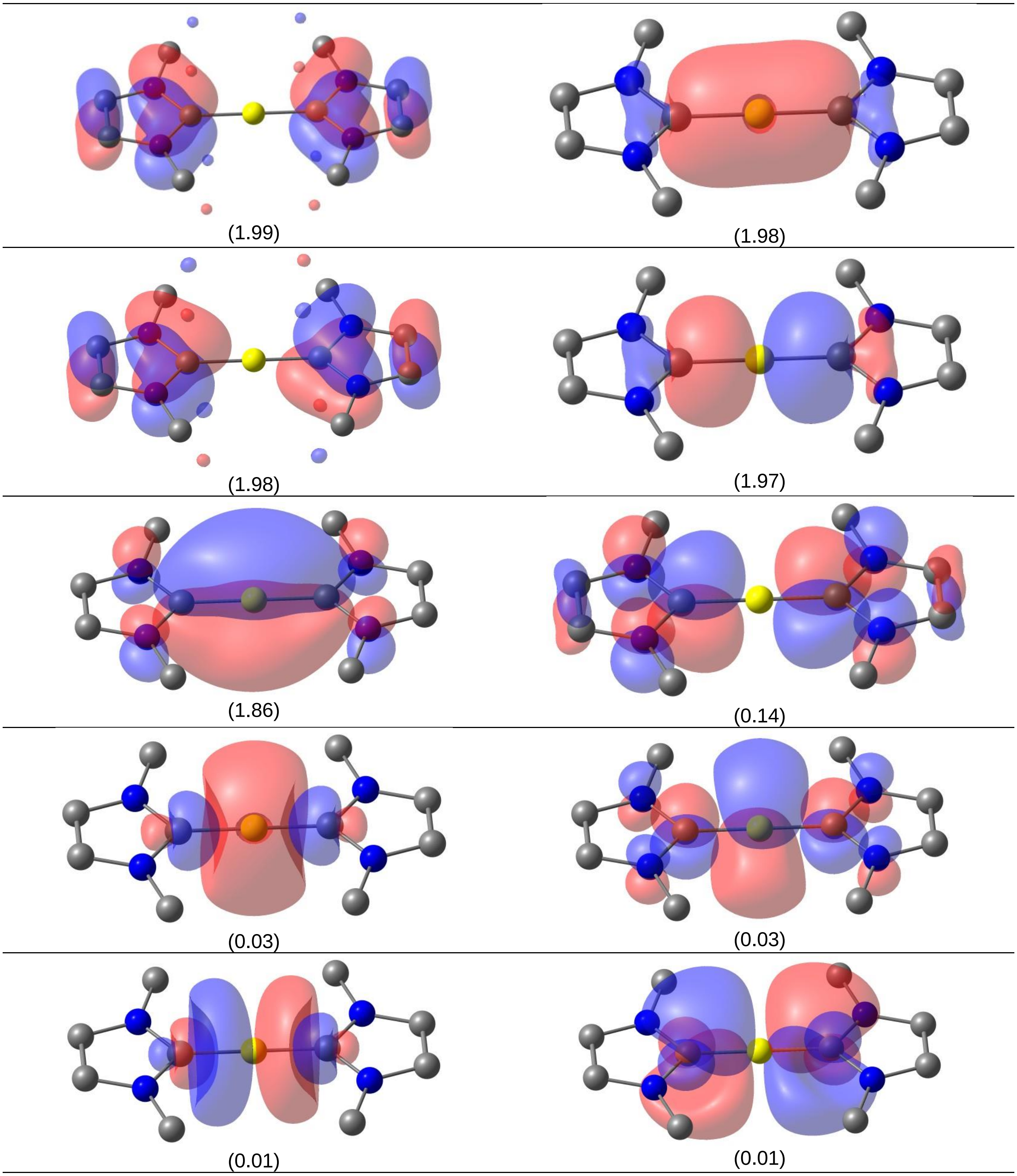

CI Coefficients: (2222200000) = -0.9457, (2222020000) = 0.2252, (2202220000) = 0.0544

Figure S48. Active-space molecular orbitals with their occupancies (in e) and CI coefficients for the singlet spin-state **Be-NHC^{Me}** obtained at the CASSCF(10,10)/cc-pVDZ//SS-CASPT2(10,10)/cc-pVDZ level of theory. Isocontour value of 0.05 a.u. Hydrogen atoms were omitted for clarity.

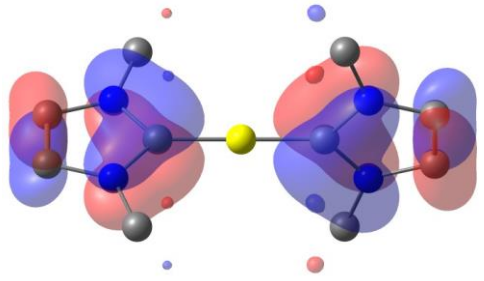
(1.98)

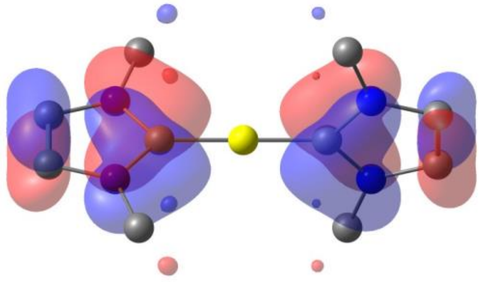
(1.98)

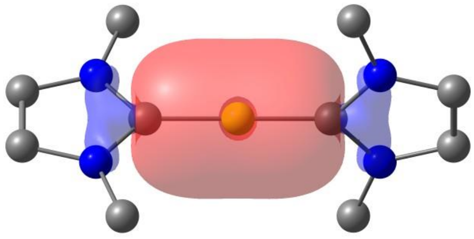
(1.98)

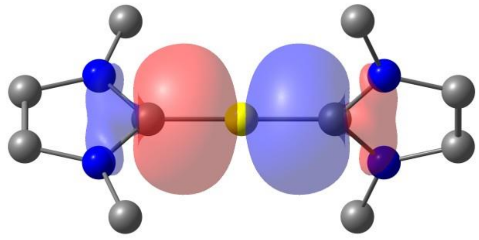
(1.97)

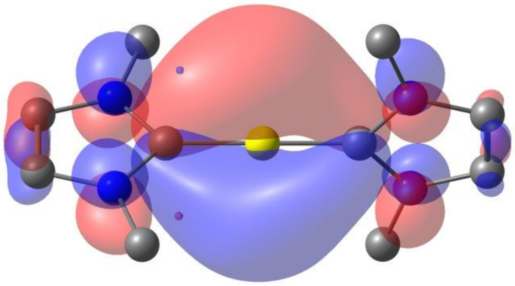
(1.00)

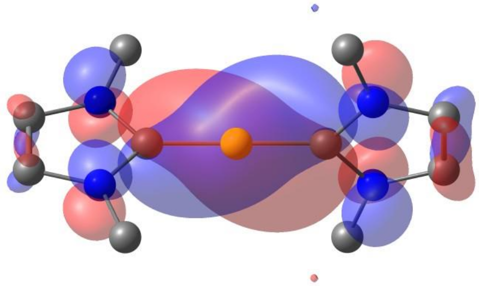
(1.00)

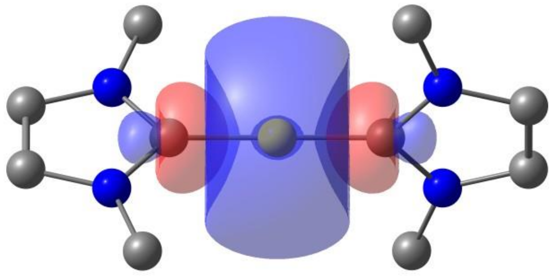
(0.03)

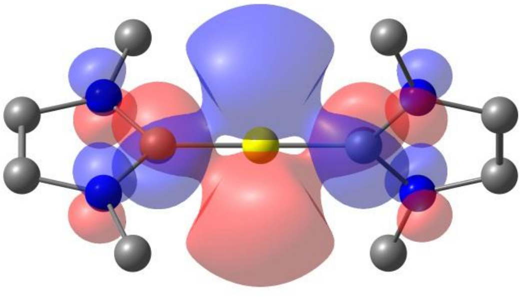
(0.02)

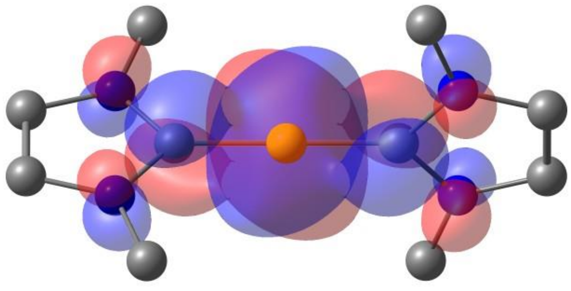
(0.02)

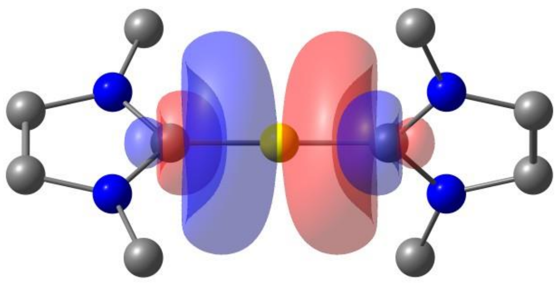
(0.01)

CI Coefficients: (2222110000) = -0.9742

Figure S49. Active-space molecular orbitals with their occupancies (in e) and CI coefficients for the triplet spin-state **Be-NHC^{Me}** obtained at the CASSCF(10,10)/cc-pVDZ//SS-CASPT2(10,10)/cc-pVDZ level of theory. Isocontour value of 0.05 a.u. Hydrogen atoms were omitted for clarity.

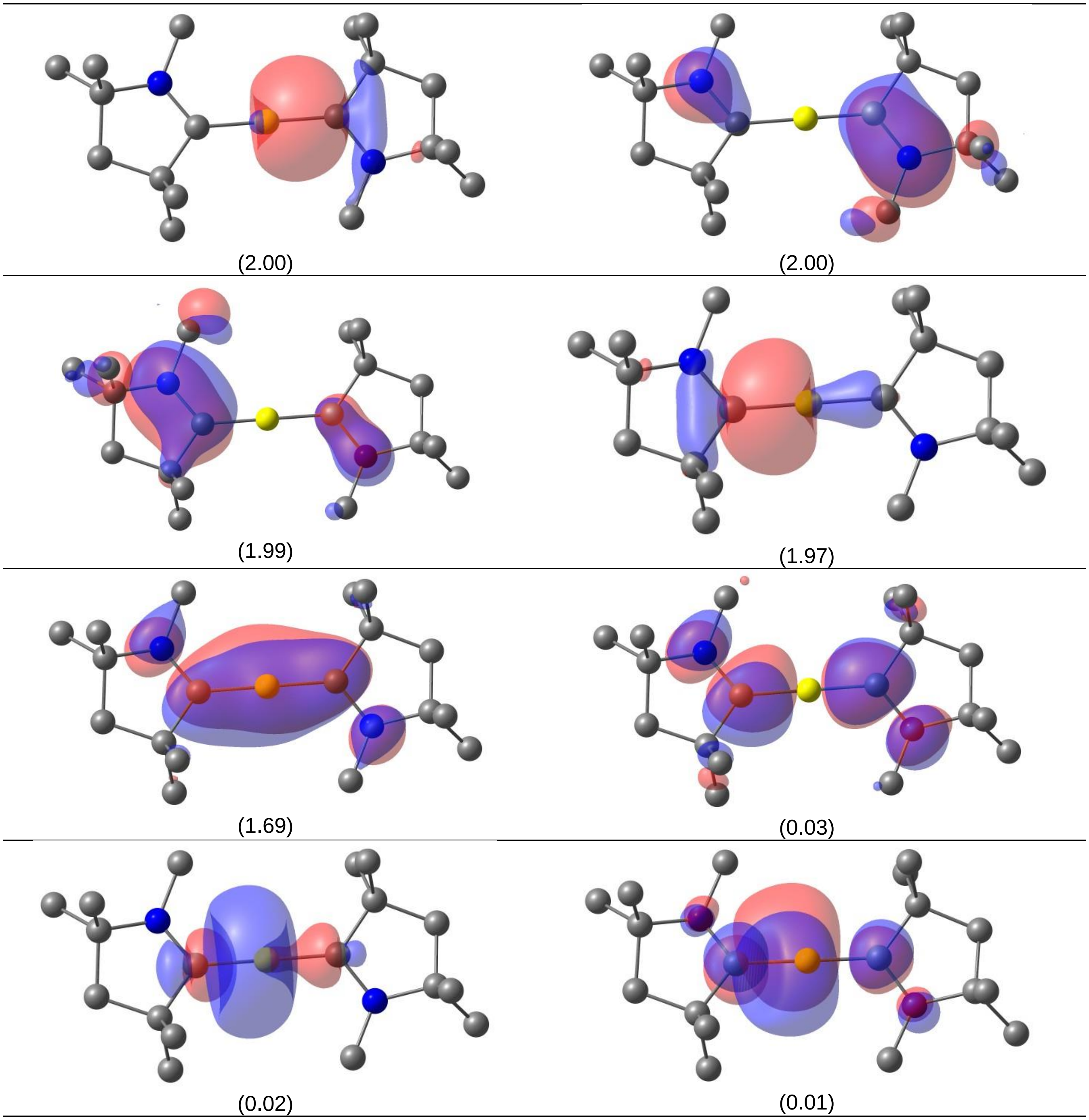

CI Coefficients: (22222000) = -0.9106, (2222020000) = 0.3780, (22202020) = 0.0659

Figure S50. Active-space molecular orbitals with their occupancies (in e) and CI coefficients for the singlet spin-state **Be-cAAC^{Me}** obtained at the CASSCF(10,8)/cc-pVDZ//SS-CASPT2(10,8)/cc-pVDZ level of theory. Isocontour value of 0.05 a.u. Hydrogen atoms were omitted for clarity.

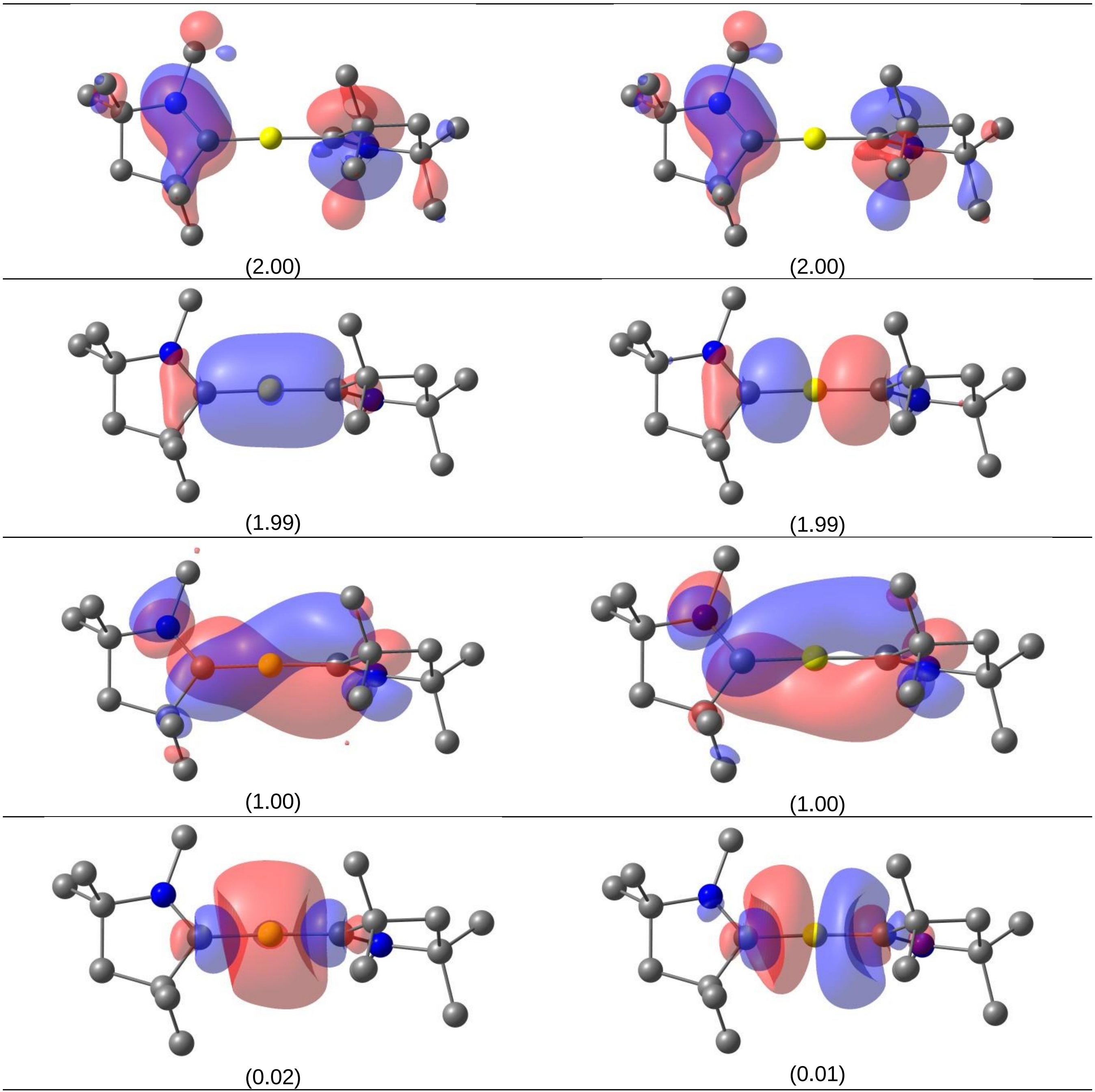

CI Coefficients: (22221100) = 0.9927, (22201120) = -0.0554

Figure S51. Active-space molecular orbitals with their occupancies (in e) and CI coefficients for the triplet spin-state **Be-cAAC^{Me}** obtained at the CASSCF(10,8)/cc-pVDZ//SS-CASPT2(10,8)/cc-pVDZ level of theory. Isocontour value of 0.05 a.u. Hydrogen atoms were omitted for clarity.

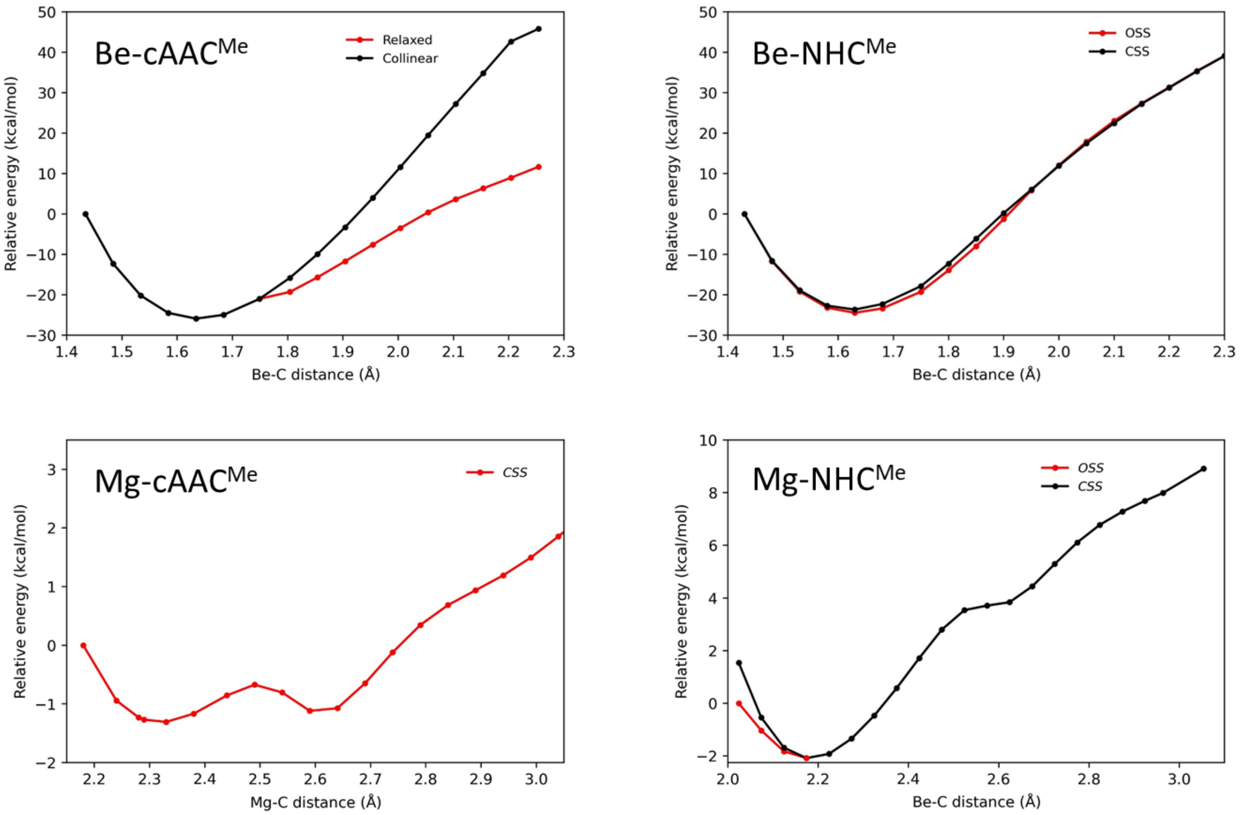

Figure S52. Potential energy surfaces (dissociation of the E-C bond) for **Be-cAAC^{Me}**, **Be-NHC^{Me}**, **Mg-cAAC^{Me}** and **Mg-NHC^{Me}** at the B3LYP-D3(BJ)/def2-TZVPP//B3LYP-D3(BJ)/def2-SVP level of theory.

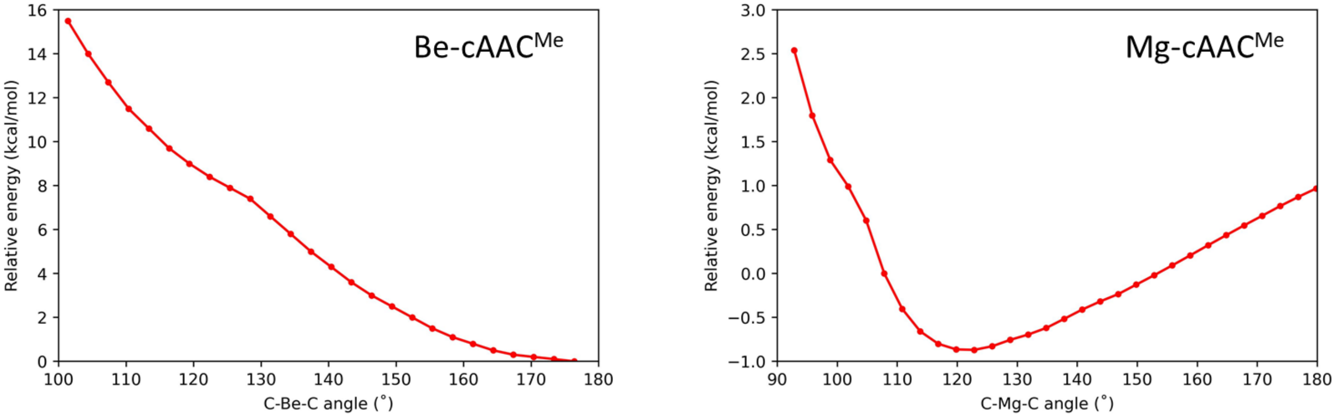

Figure S53. Potential energy surfaces (C-E-C bending) for **Be-cAAC^{Me}** and **Mg-cAAC^{Me}** at the B3LYP-D3(BJ)/def2-TZVPP//B3LYP-D3(BJ)/def2-SVP level of theory.

References

[1] (a) A. D. Becke, *Phys. Rev. A* **1988**, *38*, 3098-3100; (b) J. P. Perdew, *Phys. Rev. B* **1986**, *33*, 8822-8824.

[2] (a) A. D. Becke, *J. Chem. Phys.* **1993**, *98*, 5648-5652; (b) C. Lee, W. Yang, R. G. Parr, *Phys. Rev. B* **1988**, *37*, 785-789.

[3] (a) C. Adamo, V. Barone, *J. Chem. Phys.* **1999**, *110*, 6158-6170; (b) M. Ernzerhof, G. E. Scuseria, *J. Chem. Phys.* **1999**, *110*, 5029-5036.

[4] Y. Zhao, D. G. Truhlar, *Theo. Chem. Acc.* **2008**, *120*, 215-241.

[5] J.-D. Chai, M. Head-Gordon, *J. Chem. Phys.* **2008**, *128*, 084106.

[6] F. Weigend, R. Ahlrichs, *Phys. Chem. Chem. Phys.* **2005**, *7*, 3297-3305.

[7] S. Grimme, J. Antony, S. Ehrlich, H. Krieg, *J. Chem. Phys.* **2010**, *132*.

[8] S. Grimme, S. Ehrlich, L. Goerigk, *J. Comp. Chem.* **2011**, *32*, 1456-1465.

[9] C. Y. Peng, P. Y. Ayala, H. B. Schlegel, M. J. Frisch, *J. Comp. Chem.* **1996**, *17*, 49-56.

[10] J. W. McIver, Komornic.A, *J. Am. Chem. Soc.* **1972**, *94*, 2625-2633.

[11] K. Yamaguchi, F. Jensen, A. Dorigo, K. N. Houk, *Chem. Phys. Lett.* **1988**, *149*, 537-542.

[12] M. J. Frisch, G. W. Trucks, H. B. Schlegel, G. E. Scuseria, M. A. Robb, J. R. Cheeseman, G. Scalmani, V. Barone, G. A. Petersson, H. Nakatsuji, X. Li, M. Caricato, A. V. Marenich, J. Bloino, B. G. Janesko, R. Gomperts, B. Mennucci, H. P. Hratchian, J. V. Ortiz, A. F. Izmaylov, J. L. Sonnenberg, D. Williams-Young, F. Ding, F. Lipparini, F. Egidi, J. Goings, B. Peng, A. Petrone, T. Henderson, D. Ranasinghe, V. G. Zakrzewski, J. Gao, N. Rega, G. Zheng, W. Liang, M. Hada, M. Ehara, K. Toyota, R. Fukuda, J. Hasegawa, M. Ishida, T. Nakajima, Y. Honda, O. Kitao, H. Nakai, T. Vreven, K. Throssell, J. J. A. Montgomery, J. E. Peralta, F. Ogliaro, M. J. Bearpark, J. J. Heyd, E. N. Brothers, K. N. Kudin, V. N. Staroverov, T. A. Keith, R. Kobayashi, J. Normand, K. Raghavachari, A. P. Rendell, J. C. Burant, S. S. Iyengar, J. Tomasi, M. Cossi, J. M. Millam, M. Klene, C. Adamo, R. Cammi, J. W. Ochterski, R. L. Martin, K. Morokuma, O. Farkas, J. B. Foresman, D. J. Fox, Gaussian 16, Revision C.01. Gaussian, Inc., Wallingford CT, **2019**.

[13] (a) T. H. Dunning Jr, *J. Chem. Phys.* **1989**, *90*, 1007-1023; (b) R. A. Kendall, T. H. Dunning, R. J. Harrison, *J. Chem. Phys.* **1992**, *96*, 6796-6806; (c) D. E. Woon, T. H. Dunning Jr, *J. Chem. Phys.* **1993**, *98*, 1358-1371; (d) D. E. Woon, T. H. Dunning Jr, *J. Chem. Phys.* **1994**, *100*, 2975-2988.

[14] Q. Sun, T. C. Berkelbach, N. S. Blunt, G. H. Booth, S. Guo, Z. Li, J. Liu, J. D. McClain, E. R. Sayfutyarova, S. Sharma, S. Wouters, G. K.-L. Chan, *WIREs Computational Molecular Science* **2018**, *8*, e1340.

[15] P. Salvador, E. Ramos-Cordoba, Universitat de Girona, Girona, Spain, **2012**.

[16] G. te Velde, F. M. Bickelhaupt, E. J. Baerends, C. Fonseca Guerra, S. J. A. van Gisbergen, J. G. Snijders, T. Ziegler, *J. Comp. Chem.* **2001**, *22*, 931-967.

[17] J. Krijn, E. J. Baerends, *Fit Functions in the HFS-Method* **1984**.

[18] E. Van Lenthe, E. J. Baerends, J. G. Snijders, *J. Chem. Phys.* **1993**, 99, 4597-4610.

[19] T. Shiozaki, W. Gyroffy, P. Celani, H. J. Werner, *J. Chem. Phys.* **2011**, 135, 081106.

[20] H.J. Werner, W. Győrffy, T. Shiozakia, G. Knizia, *J. Chem. Phys.* **2013**, 138, 104104.

[21] T. Shiozaki, *WIREs Comput. Mol. Sci.* **2018**, 8.
